# Lignans and sesquiterpenoids from the stems of *Schisandra bicolor* var. *tuberculata*

**DOI:** 10.1007/s13659-022-00342-3

**Published:** 2022-05-13

**Authors:** Shui-Mei Zhang, Kun Hu, Xiao-Nian Li, Han-Dong Sun, Pema-Tenzin Puno

**Affiliations:** 1 State Key Laboratory of Phytochemistry and Plant Resources in West China, Yunnan Key Laboratory of Natural Medicinal Chemistry, Kunming Institute of Botany, Chinese Academy of Sciences, Kunming, 650201 People’s Republic of China; 2 University of Chinese Academy of Sciences, Beijing, 10039 People’s Republic of China

**Keywords:** *Schisandra bicolor* var. *tuberculata*, Cadinane-type sesquiterpenoids, Lignans, Quantum chemical calculation

## Abstract

A pair of new tetrahydrofuran lignan enantiomers, (±)-schibiculatin A [(±)-**1**], a new enedione lignan, schibiculatin B (**2**), two new cadinane-type sesquiterpenoids, schibiculatins C (**3**) and D (**4**), along with two known *seco*-cadinane-type sesquiterpenoids (**5** and **6**) and seven known miscellaneous lignans (**7**–**13**) were isolated from the stems of *Schisandra bicolor* var. *tuberculata*. The structures of **1**–**4** were elucidated by comprehensive analysis of their spectroscopic data, quantum chemical calculations, as well as single-crystal X-ray diffraction. A few isolated compounds were tested for their protective activities against corticosterone-induced apoptosis in PC12 cells. Among them, compounds **5** and **6** showed moderate activities.

**Graphical Abstract:**

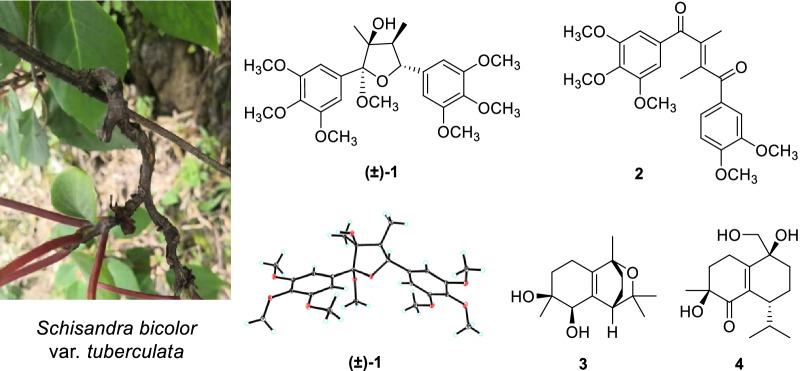

**Supplementary Information:**

The online version contains supplementary material available at 10.1007/s13659-022-00342-3.

## Introduction

The family Schisandraceae, including genera *Schisandra* and *Kadsura*, is a medicinally important plant taxon widely distributed in Southeastern Asia and Southeastern America. Previous phytochemical investigations on Schisandraceae species have led to the discovery of an array of structurally diverse natural products, including lignans [1, 2], triterpenoids [3–6], sesquiterpenoids [7, 8], etc. These compounds were found to exhibit various beneficial biological functions, including anti-HIV activity [9], hepatoprotective activity [1, 10], anti-hepatitis activity [11], neuroprotective activity [6, 12], inhibitory activity on proliferation of rheumatoid arthritis-fibroblastoid synovial cells [13], and so on.

**Fig. 1:**
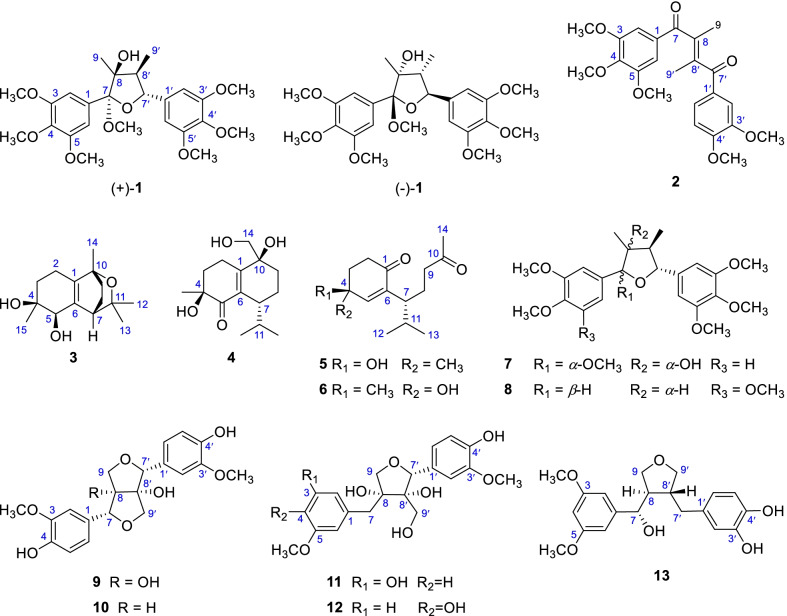
Chemical structures of compounds **1**–**13**

*Schisandra bicolor* var. *tuberculata* is a climbing plant widely distributed in Guizhou, Hunan, and Jiangxi Provinces of China. Several studies have been undertaken to explore the chemical constituents and their bioactivities of *S. bicolor* var. *tuberculata* and its original variant, *S. bicolor*. For example, two new 3,4-*seco*-cycloartane triterpenoids from the stems of *S. bicolor* were reported by Qin et al. [14]; seven described cadinane-type sesquiterpenoids were isolated by Huang et al. from the stems of *S. bicolor*, and most of them were considered as chemotaxonomic markers of this species [7]; tetrahydrofuran-type [15], dibenzylbutane-type [16], and dibenzocyclooctane-type lignans have also been obtained from *S. bicolor* var. *tuberculata* or *S*. *bicolor*, and some of them were found to exhibit statistically significant neuroprotective activities [12]. Considering that *S. bicolor* var. *tuberculata* has rarely been phytochemically investigated, in the current research, *S.* var. *tuberculata* collected from Guizhou Province, China was subjected to phytochemical study to search for structurally and biologically fascinating molecules. As a result, a pair of new tetrahydrofuran lignan enantiomers, (±)-schibiculatin A [(±)-**1**], a new enedione lignan, schibiculatin B (**2**), two new cadinane-type sesquiterpenoids, schibiculatins C (**3)** and D (**4**), along with two known *seco*-cadinane-type sesquiterpenoids (**5** and **6**) and seven known miscellaneous lignans (**7**–**13**) were obtained (Fig. 1). The structures of new compounds were elucidated by comprehensive analysis of their spectroscopic data, quantum chemical calculations, as well as single-crystal X-ray diffraction. This paper deals with the isolation, structural characterization, and bioactivity evaluation of these compounds.

## Results and discussion

**Fig. 2:**
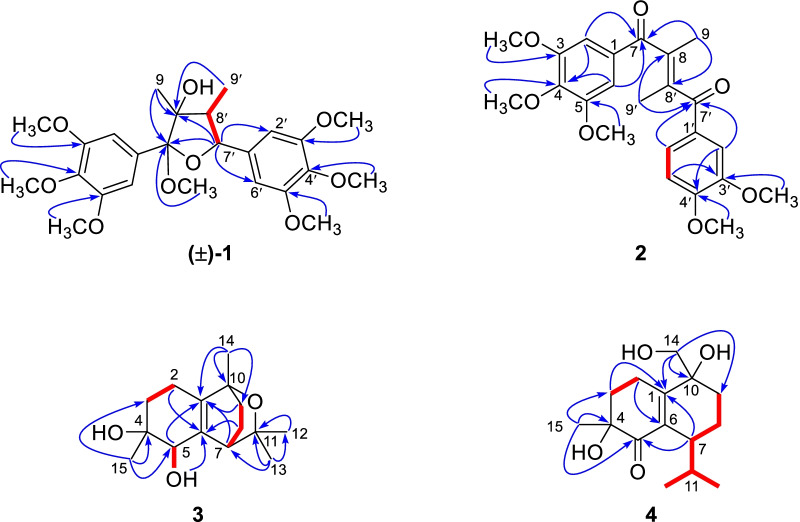
^1^H–^1^H COSY (red lines) and selected HMBC (blue arrows H → C) of compounds **1**–**4**

**Fig. 3:**
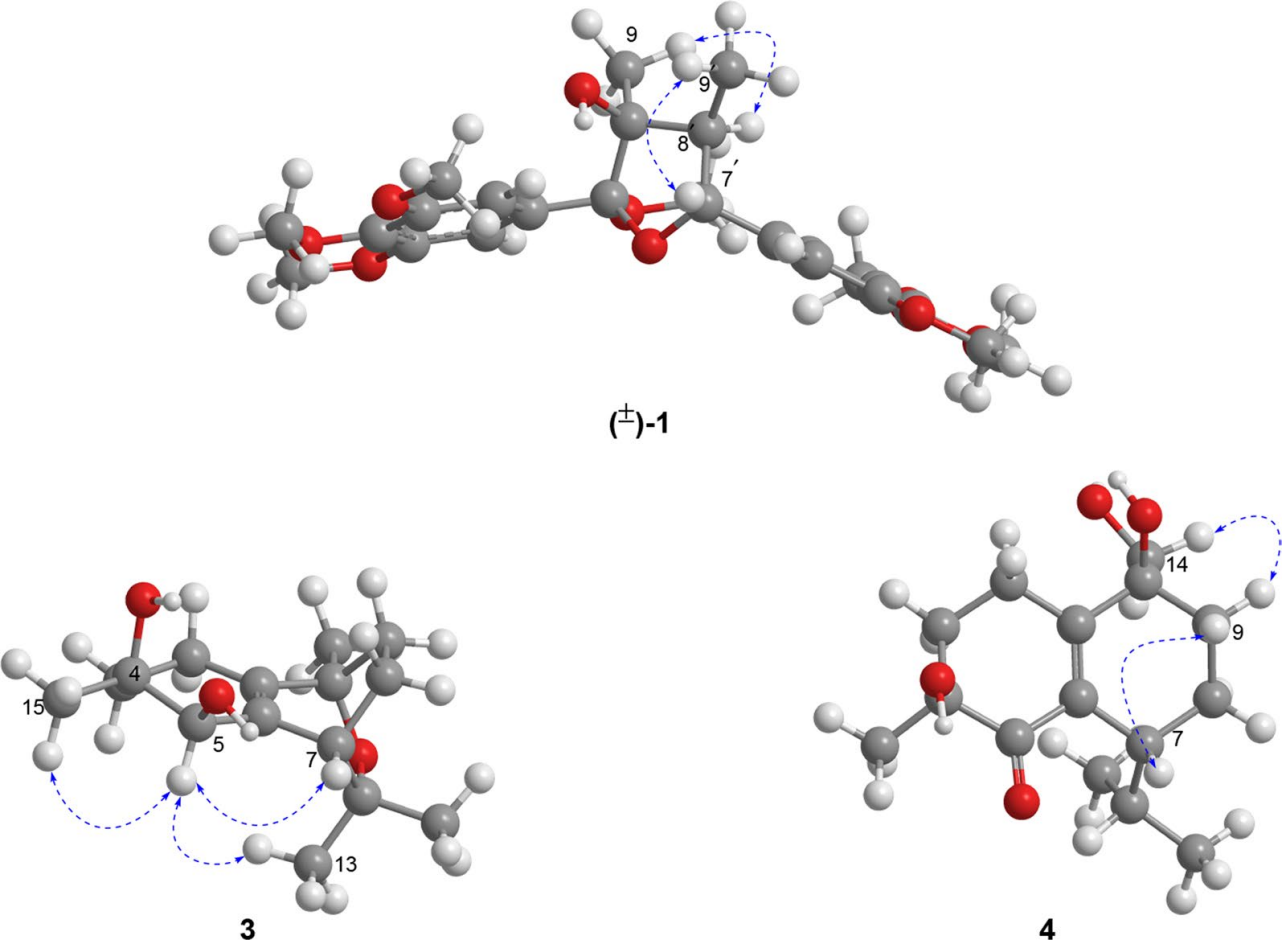
Key ROESY correlations (blue dashed double-headed arrow) of (±)-**1**, **3**, and **4**

**Fig. 4:**
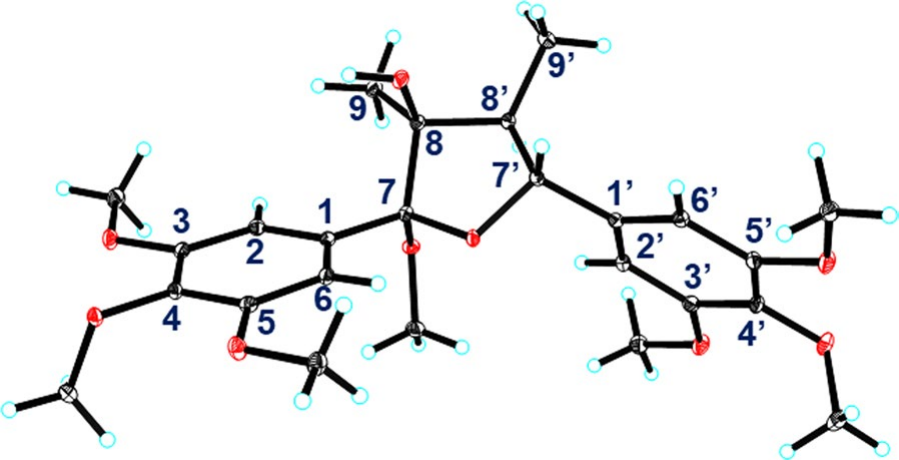
X-ray structure of (±)-**1**

**Fig. 5:**
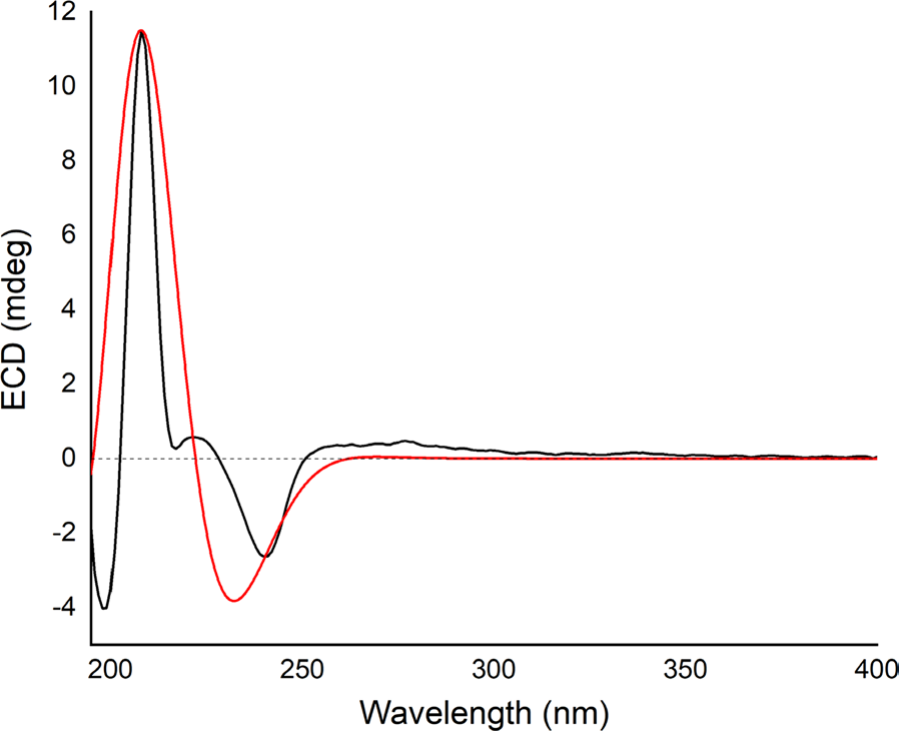
Experimental ECD spectrum of (−)**-1** (black); calculated ECD spectrum of (7*S*,8*S*,7′*S*,8′*R*)-**1** (shift = 1 nm, red)

Compound **1** (schibiculatin A) was obtained as colorless crystals. It was assigned the molecular formula of C_25_H_34_O_9_ according to the ion peak at *m/z* 501.2102 ([M+Na]^+^, calcd for 501.2095) in the HR-ESI-MS analysis and its ^13^C NMR spectrum, suggesting nine degrees of unsaturation. The IR spectrum showed characteristic absorption peaks for hydroxyl group (3436 cm^−1^) and aromatic moieties (1630, 1594, 1507, 1463 cm^−1^). The ^1^H NMR spectrum showed two methyl groups (*δ*_H_ 0.94 and 1.34); two methine protons (*δ*_H_ 2.44 and 4.81); four aromatic protons [*δ*_H_ 6.75 (2H), 6.83 (2H)]; and seven methoxy groups [*δ*_H_ 3.23 (3H), 3.77 (6H), 3.86 (12H)]. Besides, the ^13^C and DEPT NMR spectra showed signals for two methyls (*δ*_C_ 8.8 and 19.4), two methines (*δ*_C_ 50.4 and 89.0), two non-protonated carbons (*δ*_C_ 83.6 and 113.4), as well as eight resonances for aromatic carbons (*δ*_C_ 105.8, 107.2, 134.1, 138.7, 138.9, 139.0, 154.0, and 154.5). The above information (Table 1), in combination with the HMBC spectrum, suggested that compound **1** is a tetrahydrofuran lignan with two 3,4,5-trimethoxyphenyl groups. A comprehensive analysis of the ^1^H–^1^H COSY and HMBC spectra (Fig. 2) indicated that the structure of **1** is closely related to that of kalongirin A [17], and the main difference was that the latter possessed only one symmetric trimethoxyphenyl ring. The HMBC correlations from H-7′ to C-7 and C-8, from 7-OCH_3_ (*δ*_H_ 3.23) to C-7, from H_3_-9 to C-7 and C-8, in combination with the H-7′/H-8′/H-9′ spin system revealed by the ^1^H–^1^H COSY spectrum, confirmed the tetrahydrofuran moiety in **1**. The HMBC correlations from six methoxy groups to C-3, C-4, C-5, C-3′, C-4′, and C-5, respectively, along with the HMBC correlations from H-7′ to C-2′ and C-6′, confirmed the existence of two 3,4,5-trimethoxyphenyl groups and their attachment to C-7 and C-7′, respectively. The key correlations between H-9 and H-8′, H-9 and CH_3_O-7, H-7′ and H-9′ in the ROESY spectrum (Fig. 3), along with the ^3^*J*_H-7′/H-8′_ (10.2 Hz) revealed the relative configuration of **1** to be 7S*, 8S*, 7′S*, and 8′R*. Thus, **1** was determined as (7S*,8S*,7′S*,8′R*)-8-hydroxy-3,4,5,7,3′,4′,5′-heptamethoxy-7,7′-epoxylignan. Crystals of **1** were obtained and subjected to X-ray diffraction analysis (Fig. 4), which not only proved the established structure, but also revealed that it was a racemic mixture. Chiral resolution of **1** afforded (+)-**1** and (−)-**1**, and their absolute configurations were determined through TDDFT ECD calculation. According to the computational result (Fig. 5), the absolute configuration of (+)-**1** was determined as 7*R*, 8*R*, 7′*R*, and 8′S, and that of (−)-**1** was determined as 7*S*, 8*S*, 7′*S*, and 8′*R*.

**Table 1:** ^1^H and ^13^C NMR spectroscopic data for compounds** 1** and **2** (*δ* in ppm, *J* in Hz)

No.	(±)-**1**		**2**	
	*δ*_C_^a^^,c^	*δ*_H_^a^^,d^, (mult *J*)	*δ*_C_^b^^,c^	*δ*_H_^b^^,d^, (mult *J*)
1	138.7, C	–	132.3, C	–
2/6	107.2, CH	6.83 (s)	106.6, CH	6.71 (s)
3/5	134.1, C	–	152.7, C	–
4	154.0, C	–	142.3, C	–
7	113.4, C	–	197.9, C	–
8	83.6, C	–	139.4, C	–
9	19.4, CH_3_	1.34 (s)	17.5, CH_3_	2.17 (overlap)
1′	138.9, C	–	130.4, C	–
2′	105.8, CH	6.75 (s)	110.2, CH	6.93 (s)
3′	139.0, C	–	148.9, C	–
4′	154.5, C	–	153.3, C	–
5′	139.0, C	–	109.5, CH	6.74 (d, 8.3)
6′	105.8, CH	6.75 (s)	124.9, CH	7.18 (d, 8.3)
7′	89.0, CH	4.81 (d, 10.2)	197.7, C	–
8′	50.4, CH	2.44 (dq, 10.2, 6.8)	138.0, C	–
9′	8.8, CH_3_	0.94 (d, 6.8)	17.3, CH_3_	2.17 (overlap)
7-OCH_3_	50.7, CH_3_	3.23 (s)	–	–
3/5-OCH_3_	56.6, CH_3_	3.86 (s)	56.1, CH_3_	3.78 (s)
4-OCH_3_	61.0, CH_3_	3.77 (s)	60.9, CH_3_	3.87 (s)
3′-OMe	56.6, CH_3_	3.86 (s)	55.8, CH_3_	3.77 (s)
4′-OMe	61.1, CH_3_	3.77 (s)	56.0, CH_3_	3.90 (s)
5′-OMe	56.6, CH_3_	3.86 (s)	–	–

^a^Recorded in methanol-*d*_4_

^b^Recorded in chloroform-*d*

^c^Recorded at 150 MHz

^d^Recorded at 600 MHz

Compound **2** (schibiculatin B) was obtained as a colorless oil. Its molecular formula was assigned as C_23_H_26_O_7_ according to the sodium adduct ion at *m/z* 437.1566 ([M+Na]^+^, calcd for 437.1571) in the (+)-HR-ESI-MS spectrum, indicating eleven degrees of unsaturation. The IR spectrum showed characteristic absorption peaks for aromatic moieties (1649, 1584, 1511, 1463, and 1416 cm^−1^), and carboxyl groups (1737 and 1722 cm^−1^). Its ^1^H NMR spectrum (Table 1) showed two methyl groups [*δ*_H_ 2.17 (6H)] and five aromatic protons [*δ*_H_ 6.71 (2H), 6.74, 6.93, 7.18], and five methoxy groups (*δ*_H_ 3.77, 3.78 (6H), 3.87, 3.90). The ^13^C NMR, DEPT, and HSQC spectra exhibited 23 carbons (Table 1), including two methyl groups (*δ*_C_ 17.3 and 17.5), fourteen olefinic carbons [*δ*_C_ 106.6 (2C), 109.5, 110.2, 124.9, 130.4, 132.3, 138.0, 139.4, 142.3, 148.9, 152.7 (2C), 153.3], two carboxyl groups (*δ*_C_ 197.7 and 197.9), and five methoxy groups [*δ*_C_ 55.8, 56.0, 56.1 (2C), 60.9]. The ^1^H and ^13^C NMR spectra of **2** were similar to those of *threo*-2-methyl-3-oxo-1-(3′,4′,5′-trimethoxyphenyl) butyl-3″,4″-dimethoxybenzoate [18], and the main difference was the substitution patterns of two aromatic rings. Careful analysis of the above information (Table 1) suggested the presence of a 3,4,5-trimethoxyphenyl group and a 3,4-dimethoxyphenyl group in **2**. The HMBC correlations from H-2, H-6 to C-7 (*δ*_C_ 197.9), and from H-2′, H-6′ to C-7′ (*δ*_C_ 197.7) assigned the locations of two carboxyl groups. In addition, the HMBC correlations from H-9 to C-7 and C-8′, from H-9′ to C-8 and C-7′ established the C-8/C-8′ tetrasubstituted double bond. However, the geometry of C-8/C-8′ double bond can’t be determined through ROESY correlations due to the overlap of H-9 and H-9′ in ^1^H NMR spectrum. Thus, two geometric isomers of **2**, 8*E*-**2** (**2a**) and 8*Z*-**2** (**2b**) were subjected to quantum chemical calculation of NMR chemical shifts. According to the computational results, including R^2^, MAE, CMAE [19], and DP4+ probability [20] (Table 3), the C-8/C-8′ double bond was determined to adopt *E* geometry. Thus, **2** was determined as (*E*)-1-(3′,4′,5′-trimethoxyphenyl)-4-(3″,4″-dimethoxyphenyl)-2,3-dimethylbut-2-ene-1,4-dione.

Compound **3** (schibiculatin C) was obtained as a colorless oil. Its molecular formula was established as C_15_H_24_O_3_ by analysis of its (+)-HR-ESI-MS data ([M+Na]^+^ *m/z* 275.1621, calcd for 275.1618), suggesting four degrees of unsaturation. The IR spectrum of **3** showed a characteristic absorption peak (3428 cm^−1^) for a hydroxy group. The ^1^H spectrum showed four singlet methyl groups (*δ*_H_ 1.17, 1.32, 1.36, and 1.64); two methine protons (*δ*_H_ 2.76 and 4.44), two hydroxyl protons (*δ*_H_ 5.72 and 6.37). The ^13^C NMR and DEPT spectra, in combination with the HSQC spectrum showed fifteen carbon resonances, including four methyls (*δ*_C_ 22.2, 24.5, 28.4, and 29.4), four methylenes (*δ*_C_ 21.3, 22.2, 32.8, and 33.8), two methines (*δ*_C_ 41.5 and 76.4), and five non-protonated carbons (including three oxygenated methines at *δ*_C_ 71.8, 73.1, and 74.1; two olefinic carbons at *δ*_C_ 135.6 and 140.2). The aforementioned NMR data of **3** (Table 2) indicated it is a cadinane-type sesquiterpene structurally similar to (4*R*,5*R*,10*R*)-10-methoxymuurol-1(6)-ene-4,5-diol [21]. The ^1^H–^1^H COSY spectrum of **3** revealed the presence of three spin systems (Fig. 2) through H-2a (*δ*_H_ 2.53)/H-3b (*δ*_H_ 1.97), HO-5 (*δ*_H_ 6.37)/H-5 (*δ*_H_ 4.44), and H-7 (*δ*_H_ 2.76)/H-8b (*δ*_H_ 1.52)/H-9a (*δ*_H_ 1.76) correlations. In the HMBC spectrum of **3** (Fig. 2), correlations from CH_3_-13 (*δ*_H_ 1.17) to C-7 (*δ*_C_ 41.5), C-11 (*δ*_C_ 74.1), and C-12 (*δ*_C_ 28.4); from CH_3_-14 (*δ*_H_ 1.36) to C-1 (*δ*_C_ 135.6), C-9 (*δ*_C_ 32.8), and C-10 (*δ*_C_ 73.1); from CH_3_-15 (*δ*_H_ 1.64) to C-3 (*δ*_C_ 33.8), C-4 (*δ*_C_ 71.8), and C-5 (*δ*_C_ 76.4) indicated that two methyls and one isopropyl group were attached to C-4, C-10, and C-7, respectively. Besides, the HMBC correlations (Fig. 2) from H-3a, H-3b, H-5, H-7, H-9a and H-9b to C-1; and from H-2, H-8, and HO-5 to C-6 (*δ*_C_ 140.2), suggested the presence of the C-1/C-6 double bond. The remaining one degree of unsaturation implied the existence of an epoxy ring, which was deduced to form between C-10 and C-11 according to molecular modeling. Assigning H-5 as *α*-oriented, the H-7/H-5*α*, CH_3_-13/H-5*α*, CH_3_-15/H-5*α* correlations (Fig. 3) in the ROESY spectrum indicated that both CH_3_-15 and the epoxy ring adopt *α*-orientation. Subsequently, quantum chemical calculation of NMR chemical shifts succeeded in confirming the established the structure of **3** (Table 3). However, TDDFT calculation failed to produce the experimental ECD curve of **3**, despite that several independent methods have been tried. Thus, the absolute configuration of **3** was left undermined. The structure of **3** was established as 10,11-epoxy-cadin-1(6)-ene-4,5-diol.

**Table 2:** ^1^H and ^13^C NMR spectroscopic data for compounds **3** and **4** (*δ* in ppm, *J* in Hz)

No.	**3**		**4**	
	*δ*_C_^a^^,c^	*δ*_H_^a^^,d^, (mult *J*)	*δ*_C_^b^^,c^	*δ*_H_^b^^,d^, (mult *J*)
1	135.6, C	–	159.6, C	–
2	22.2, CH_2_	2.53 (m) 2.07 (overlap)	28.3, CH_2_	2.58 (overlap)
3	33.8, CH_2_	2.07 (overlap) 1.97 (m)	36.3 CH_2_	1.99 (m) 1.92 (m)
4	71.8, C	–	73.4, C	–
5	76.4, CH	4.44 (s)	202.7, C	–
6	140.2	–	137.2, C	–
7	41.5, CH	2.76 (t, 2.7)	38.7, CH	2.58 (overlap)
8	21.3, CH_2_	2.07 (overlap) 1.52 (m)	20.2, CH_2_	1.62 (m)
9	32.8 CH_2_	1.76 (m) 1.33 (overlap)	31.3, CH_2_	2.22 (m) 1.41 (m)
10	73.1, C	–	74.1, C	–
11	74.1, C	–	30.7, CH	2.10 (dq, 13.6, 6.8)
12	28.4, CH_3_	1.32 (overlap)	21.4, CH_3_	0.85 (d, 6.8)
13	29.4, CH_3_	1.17 (s)	19.1, CH_3_	0.79 (d, 6.8)
14	22.2, CH_3_	1.36 (s)	67.1, CH_2_	3.66 (d, 11.2) 3.49 (d, 11.2)
15	24.5, CH_3_	1.64 (s)	24.1, CH_3_	1.24 (s)
4-OH	–	5.72 (s)	–	–
5-OH	–	6.37 (d, 5.7)	–	–

^a^Recorded in pyridine-*d*_5_

^b^Recorded in methanol-*d*_4_

^c^Recorded at 150 MHz

^d^Recorded at 600 MHz

**Table 3:** The results for NMR computation of compounds **2**–**4**

Parameters	^13^C data			^1^H data			DP4+ probability (all data)^b^
	R^2a^	MAE	CMAE	R^2^	MAE	CMAE	
**2a**	0.9988	2.2	1.5	0.9959	0.45	0.09	100.00%
**2b**	0.9972	2.6	1.9	0.9914	0.20	0.12	0.00
**3**	0.9993	1.0	0.8	0.9506	0.33	0.15	–
**4a**	0.9992	1.7	1.1	0.9704	0.15	0.10	80.93%
**4b**	0.9988	1.8	1.6	0.9810	0.10	0.09	6.51%
**4c**	0.9977	2.1	2.1	0.9722	0.17	0.10	12.52%
**4d**	0.9989	1.7	1.5	0.9563	0.14	0.11	0.03%

*MAE: * Mean Absolute Error, *CMAE*: Corrected Mean Absolute Error

^a^R^2^: squared Pearson correlation coefficient between experimental and calculated NMR chemical shifts

^b^DP4+ probability (all data): DP4+ probability based on analyzing ^1^H or ^13^C data together. If ^1^H chemical shifts of CH_2_, which were prone to error when assigning, are removed from analysis, the DP4+ probability can be more distinguishing (Figures S49 and S50)

Compound **4** (schibiculatin D) was isolated as a colorless oil. Its molecular formula was assigned as C_15_H_24_O_4_ according to the sodium adduct ion peak at *m/z* 291.1566 ([M+Na]^+^, calcd 291.1567) in the (+)-HR-ESI-MS spectrum, indicating four degrees of unsaturation. The IR spectrum showed characteristic absorption peaks for hydroxyl group (3427 cm^−1^), double bond (1462, 1611 cm^−1^), and carboxyl group (1663 cm^−1^). Its ^1^H NMR spectrum showed three methyl groups (*δ*_H_ 0.79, 0.85, and 1.24) and two oxygenated protons (*δ*_H_ 3.49 and 3.66). The ^13^C NMR, DEPT, and HSQC spectra exhibited 15 carbon resonances, including three methyl groups (*δ*_C_ 19.1, 21.4, and 24.1), five methylenes (*δ*_C_ 20.2, 23.8, 31.3, 36.3, and 67.1), two methines (*δ*_C_ 30.7 and 38.7), five non-protonated carbons (including two oxygenated methines at *δ*_C_ 73.4 and 74.1; two olefinic carbons at *δ*_C_ 137.2 and 159.6; one carboxyl carbon at *δ*_C_ 202.7). The aforementioned NMR data of **4** (Table 2) revealed that it was also a cadinane-type sesquiterpene. The structure of **4** was found to be closely related to that of (4*α*,10*β*)-4,10-dihydroxycadin-1(6)-ene-5-one [22], with the main difference being that the CH_3_-14 in the latter was replaced by a hydroxy group-substituted methylene in **4**. The above observation could be further confirmed by key HMBC correlations from H-14a (*δ*_H_ 3.66) and H-14b (*δ*_H_ 3.49) to C-1 (*δ*_C_ 159.6), C-9 (*δ*_C_ 31.3), and C-10 (*δ*_C_ 74.1). However, due to signal overlap in the ^1^H spectrum (H-7 and H-2; H-8a and H-8b, for example), it was difficult to elucidate the relative configuration of **4** through ROESY correlations. Then, four possible diastereoisomers of **4**, (4S*,7R*,10S*)-**4** (**4a**), (4S*,7R*,10R*)-**4** (**4b**), (4S*,7S*, 10R*)-**4** (**4c**), and (4S*,7S*,10S*)-**4** (**4d**) were subjected to quantum chemical calculation of NMR chemical shifts. According to the computational results (Table 3), the relative configuration of **4** was determined as 4S*, 7R*, and 10S*. However, as the situation in **3**, TDDFT calculation failed to produce the experimental ECD curve of **4**. Thus, the absolute configuration of **4** was left undermined. Thus, the structure of **4** was determined as 4,10,14-trihydroxycadin-1(6)-en-5-one.

The structures of those known compounds, including (4*R*)-4-hydroxy-1,10-*seco*-muurol-5-ene-1,10-dione (**5**) [21], (4*S*)-4-hydroxy-1,10-*seco*-muurol-5-ene-1,10-dione (**6**) [21], kadlongirin A (**7**) [17], rel-(2*R*,3*R*,4*S*,5*S*)-tetrahydro-3,4-dimethyl-2,5-bis(3,4,5-trimethoxyphenyl) furan (**8**) [23], prinsepiol (**9**) [24], 8*α*-hydroxypinoresinol (**10**) [25], ginkgool (**11**) [26], massoiresinol (**12**) [27], acanthosessilin A (**13**) [28] were identified by comparing their spectroscopic data with those reported in literature.

Additionally, compounds (−)-**1**, (+)-**1**, **2**, **3**, **5**, and **6** were evaluated for their protective effects against damage of PC12 cells induced by corticosterone. As a result, compounds **5** and **6** showed moderate activities (Table 4), which indicated their potential neuroprotective activities.

**Table 4:** Protective activities of selected compounds against corticosterone-induced apoptosis in PC12 Cells

Compound	Survival rate (%)	Standard deviation (%)	*P*
Blank	100.00	0.58	***
Negative control	59.71	0.52	
Desipramine	89.17	0.26	***
(−)-**1**	59.02	0.24	
(+)-**1**	59.71	0.43	
**2**	59.57	0.44	
**3**	59.73	0.28	
**5**	62.49	0.29	**
**6**	61.90	0.74	*

Data are analyzed by T test and F test, and presented as means ± SD (*p < 0.05, **p < 0.01, ***p < 0.001)

## Experimental section

### General

HRESIMS data were acquired on an Agilent 6540 QSTAR TOF time-of-flight mass spectrometer (Agilent Corp., America). A Tensor 27 spectrophotometer (Bruker Corp., Switzerland) was used for scanning IR spectroscopy with KBr pellets. UV spectra were obtained using a Shimadzu UV-2401 PC spectrophotometer (Shimadzu Corp., Japan). Experimental ECD spectra were measured on a Chirascan V100 instrument (Applied Photophysics Limited, Britain). Optical rotations were measured with a JASCO P-1020 polarimeter (Jasco Corp., Japan). Crystallographic data were obtained on a Bruker APEX DUO (Bruker Corp., Switzerland). 1D and 2D NMR spectra were recorded on Bruker AV III 500 MHz, Bruker DRX-600, or Bruker Ascend 800 MHz spectrometers (Bruker Corp., Switzerland) with TMS internal standard. Chemical shifts (*δ*) are expressed in ppm relative to the solvent signals. Semi-preparative HPLC was performed on an Agilent 1260 liquid chromatography (Agilent Corp., America) with a Zorbax SB-C18 (4.6 mm × 250 mm) column and a CHIRALPAK IC (4.6 mm × 250 mm) column. Column chromatography (CC) was performed with silica gel (80–100, 100–200 and 200–300 mesh; Qingdao Marine Chemical, Inc., Qingdao, People’s Republic of China). MCI gel (75–150 μm, Mitsubishi Chemical Corporation, Tokyo, Japan), Lichroprep RP-18 gel (40–63 μm, Merck, Darmstadt, Germany), Sephadex LH-20 ((Pharmacia, Uppsala, Sweden) and SEPAFlash column (Spherical C-18, 20–45 μm, 100 Åm). Fractions were monitored by thin layer chromatography and spots were detected with 10% H_2_SO_4_ in EtOH.

### Plant material

The stems of *Schisandra bicolor* var. *tuberculata* were collected in Tongren region, Guizhou Province, China, in April 2020, and identified by Prof. Heng Li, Kunming Institute of Botany. A voucher specimen has been deposited in the Herbarium of the Kunming Institute of Botany, Chinese Academy of Sciences.

### Bioactivity evaluation

Poorly differentiated PC12 cells were maintained in DMEM medium supplemented with 10% fetal bovine serum (FBS), penicillin (100 U/mL), streptomycin (100 μg/mL), and incubated at 5% CO_2_ and 37 ℃. Poorly differentiated PC12 cells were divided into the following groups: untreated, CORT (150 μmol/L), CORT (150 mol/l) plus DIM (10 μmol/L), CORT (150 μmol/L) plus test compounds (20 μmol/L). Briefly, poorly differentiated PC12 cells were seeded into 96-well culture plates at a density of 1 × 10^4^ cells/well. After 24 h culturing, the wells were added compounds as previously described groups. 48 h later, MTS solution was added to each well. The absorbance was measured at 492 nm using a Thermo Multiskan FC [29].

### Extraction and isolation

Air-dried and powdered stems (10.5 kg) of *S. bicolor* var. *tuberculata* were extracted with 70% aqueous Me_2_CO (3 × 40 l) to yield an extract at room temperature to give a crude extract (1081 g), which was extracted with EtOAc. The EtOAc part (380 g) was separated by a silica gel column and eluted with a CHCl_3_/Me_2_CO gradient system (1:0 to 0:1) to afford five fractions A–E. Fractions B (45 g) and C (51 g) were successively decolorized on MCI gel with MeOH/H_2_O (90:10) to obtain B1 and C1. Fraction B1 (42 g) was subjected to ODS chromatography and eluted with MeOH/H_2_O (50:50 to 100:0) to give six fractions (B1a–B1f). Then B1a (18 g) was repeatedly subjected to silica gel column to obtain **8** (300 mg), other fractions further purified by preparative HPLC to afford **5** (30 mg) and **6** (20.0 mg). Fraction B1b (14 g) was repeatedly subjected to silica gel column, then purified by semi-preparative HPLC to give **1** (13.5 mg), **2** (3.0 mg) and **7** (2.9 mg). Fraction C1 (44 g) was subjected to ODS chromatography by eluting with MeOH/H_2_O (30:70 to 100:0) to give seven fractions. C1b (4.52 g) was chromatographed on flash column by eluting with MeOH/H_2_O (20:80 to 60:40) to yield 12 fractions bF1–bF12, and bF2 (850 mg) was then subjected to Sephadex LH-20 (CHCl_3_/MeOH, 1:1), and further purified by semi-preparative HPLC to give **3** (3.49 mg), and **4** (4.35 mg), **9** (10 mg), **10** (2.62 mg), **11** (3.71 mg). Fraction bF4 (420 mg) was subjected to Sephadex LH-20 (CHCl_3_-MeOH, 1:1), and further purified by semi-preparative HPLC to give **12** (5.40 mg) and **13** (3.71 mg).

### Physical constants and spectroscopic data of compounds

(−)-Schibiculatin A [(−)-(**1**)]: colorless crystals, [*α*] − 37.45, (*c* 0.110, MeOH), UV (MeOH) *λ*_max_ (log *ε*) 206 (4.96), 258 (3.24), 272 (3.40) nm. CD (CH_3_OH, *c* 0.05): 198 nm (Δ*ε* =  − 11.19), 208 nm (Δ*ε* = 31.90), 217 nm (Δ*ε* = 0.73), 221 nm (Δ*ε* = 1.60), 240 nm (Δ*ε* =  − 7.31). IR (KBr) *ν*_max_ 3436, 2958, 2935, 1630, 1594, 1507, 1463, 1128 cm^−1^. ESIMS *m*/*z* 252 [M+Na]^+^, HRESIMS *m*/*z* 501.2102 [M+Na]^+^ (calcd for C_25_H_34_O_9_Na 501.2095). ^1^H NMR (methanol-*d*_4_, 600 MHz) and ^13^C NMR (methanol-*d*_4_, 150 MHz), see Table 1.

(+)-Schibiculatin A [(+)-(**1**)]: colorless crystals, [*α*] + 31.38 (*c* 0.087, MeOH), UV (MeOH) *λ*_max_ (log *ε*) 206 (4.93), 259 (3.57), 268 (3.62) nm. ECD (CH_3_OH, *c* 0.05): 198 nm (Δ*ε* = 17.07), 208 nm (Δ*ε* =  − 22.21), 217 nm (Δ*ε* = 4.14), 221 nm (Δ*ε* = 9.23), 240 nm (Δ*ε* = 3.03). IR (KBr) *ν*_max_ 3436, 2958, 2935, 1630, 1594, 1507, 1463, 1128 cm^−1^. ESIMS *m*/*z* 478 [M+Na]^+^, HRESIMS *m*/*z* 501.2102 [M+Na]^+^ (calcd for C_25_H_34_O_9_Na 501.2095). ^1^H NMR (methanol-*d*_4_, 600 MHz) and ^13^C NMR (methanol-*d*_4_, 150 MHz), see Table 1.

Schibiculatin B (**2**): colorless oil, [*α*] + 15.91 (*c* 0.093, MeOH), UV (MeOH) *λ*_max_ (log *ε*) 202 (4.60), 256 (3.71), 281 (3.91) nm. IR (KBr) *ν*_max_ 2938, 2922, 1737, 1721, 1649, 1584, 1511, 1463, 1416, 1128 cm^−1^. ESIMS *m*/*z* 437 [M+Na]^+^, HRESIMS *m/z* 437.1566 [M+Na]^+^ (calcd for C_23_H_28_O_8_Na 437.1571). ^1^H NMR (chloroform-*d*, 600 MHz) and ^13^C NMR (chloroform-*d*, 150 MHz), see Table 1.

Schibiculatin C (**3**): colorless oil, [*α*] + 25.2 (*c* 0.090, MeOH), UV (MeOH) *λ*_max_ (log *ε*) 195 (3.74) nm. ECD (CH_3_OH, *c* 0.36): 199 nm (Δ*ε* = 3.03), 214 nm (Δ*ε* =  − 2.01). IR (KBr) *ν*_max_ 3428, 2966, 2921, 2859, 1631, 1560, 1384, 802 cm^−1^. ESIMS *m*/*z* 275 [M+Na]^+^, HRESIMS *m*/*z* 275.1621 [M+Na]^+^ (calcd for C_15_H_24_O_3_Na 275.1618). ^1^H NMR (pyridine-*d*_5_, 600 MHz) and ^13^C NMR (pyridine-*d*_5_, 150 MHz), see Table 2.

Schibiculatin D (**4**): colorless oil, [*α*] − 11.2 (*c* 0.088, MeOH), UV (MeOH) *λ*_max_ (log *ε*) 216 (3.53) nm, 248 (3.88) nm. ECD (CH_3_OH, *c* 0.26): 206 nm (Δ*ε* = 2.69), 248 nm (Δ*ε* =  − 7.81), 338 nm (Δ*ε* = 1.95). IR (KBr) *ν*_max_ 3427, 2958, 2930, 2859, 1663, 1611, 1463, 1383, 1060 cm^−1^. ESIMS *m*/*z* 268 [M+Na]^+^, HRESIMS *m*/*z* 291.1566 [M+Na]^+^ (calcd for C_15_H_24_O_4_Na 291.1567). ^1^H NMR (methanol-*d*_4_, 600 MHz) and ^13^C NMR (methanol-*d*_4_, 150 MHz), see Table 2.

Crystal data for (±)-schibiculatin A [(±)-**1**]: C_25_H_34_O_9_, *M* = 478.52, *a* = 7.9385(2) Å, *b* = 9.5977(3) Å, *c* = 17.1002(5) Å, *α* = 86.9070(10)°, *β* = 84.8510(10)°, *γ* = 66.3110(10)°, *V* = 1188.06(6) Å^3^, *T* = 100.(2) K, space group *P*-1, *Z* = 2, *μ*(Cu Kα) = 0.843 mm^−1^, 39,839 reflections measured, 4665 independent reflections (*R*_*int*_ = 0.0409). The final *R*_1_ values were 0.0343 (*I* > 2*σ*(*I*)). The final *wR*(*F*^2^) values were 0.0865 (*I* > 2*σ*(*I*)). The final *R*_1_ values were 0.0368 (all data). The final *wR*(*F*^2^) values were 0.0882 (all data). The goodness of fit on *F*^2^ was 1.069. Crystallographic data for the structure of (±)-schibiculatin A [(±)-**1**] have been deposited in the Cambridge Crystallographic Data Centre (deposition number CCDC 2145390). Copies of the data can be obtained free of charge from the CCDC via www.ccdc.cam.ac.uk.

## Supplementary Information

**Additional file 1.** It includes 1D NMR, 2D NMR, HRESIMS, UV, ECD, IR, OR, and computational data of compounds **1**–**4**, and methods for quantum chemical calculations are available.
