# Supplementary material for: Lignans and sesquiterpenoids from the stems of Schisandra bicolor var. tuberculata
Source: Nat Prod Bioprospect. 2022 May 13;12(1):19. doi: 10.1007/s13659-022-00342-3 (PMC9098724; doi:10.1007/s13659-022-00342-3)
Supplement: Supplementary file 1 — Additional file 1. It includes 1D NMR, 2D NMR, HRESIMS, UV, ECD, IR, OR, and computational data of compounds 1–4, and methods for quantum chemical calculations are available. [file 13659_2022_342_MOESM1_ESM.docx]

**Supplementary Material for**

**Lignans and sesquiterpenoids from the stems of *Schisandra* *bicolor* var. *tuberculata***

Shui-Mei Zhang^1,2^, Kun Hu^1^**^*^**, Xiao-Nian Li^1^, Han-Dong Sun^1^, **Pema-Tenzin Puno^1*^**

^1^State Key Laboratory of Phytochemistry and Plant Resources in West China, Kunming Institute of Botany, Chinese Academy of Sciences, Yunnan Key Laboratory of Natural Medicinal Chemistry, Kunming, 650201, People’s Republic of China.

^2^University of Chinese Academy of Sciences, Beijing, 10039, People’s Republic of China

^*^Corresponding author Tel.: (86) 871-65223616

E-mail: [punopematenzin@mail.kib.ac.cn](mailto:punopematenzin@mail.kib.ac.cn); hukun@mail.kib.ac.cn

**Contents of Supporting Information**

| **Contents** | **Pages** |
| --- | --- |
| 1D, 2D NMR, HRESIMS, EIMS, IR, UV and CD spectra of (±)-**1** | 3−12 |
| 1D, 2D NMR, HRESIMS, EIMS, IR and UV spectra of **2** | 13−21 |
| 1D, 2D NMR, HRESIMS, EIMS, IR, UV and CD spectra of **3** | 22−31 |
| 1D, 2D NMR, HRESIMS, EIMS, IR, UV and CD spectra of **4** | 31−40 |
| Methods for quantum chemical calculations | 41−43 |
| Computational data of **1** | 43−52 |
| Computational data of **2** | 53−66 |
| Computational data of **3** | 67−73 |
| Computational data of **4** | 74−112 |
| Reference | 113−114 |


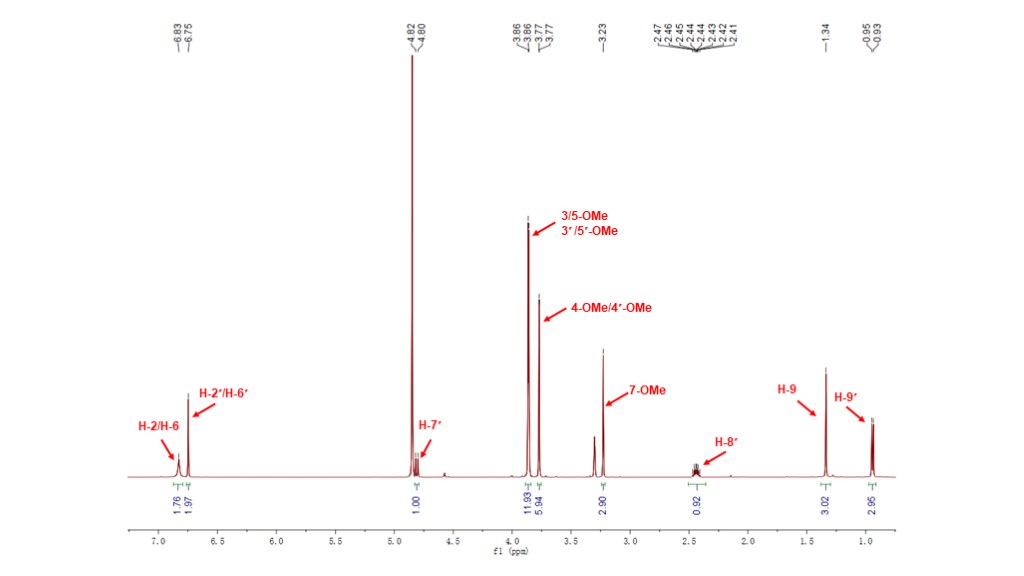

**Figure S1.** ^1^H NMR spectrum of compound (±)-**1** (methanol-*d*_4_).


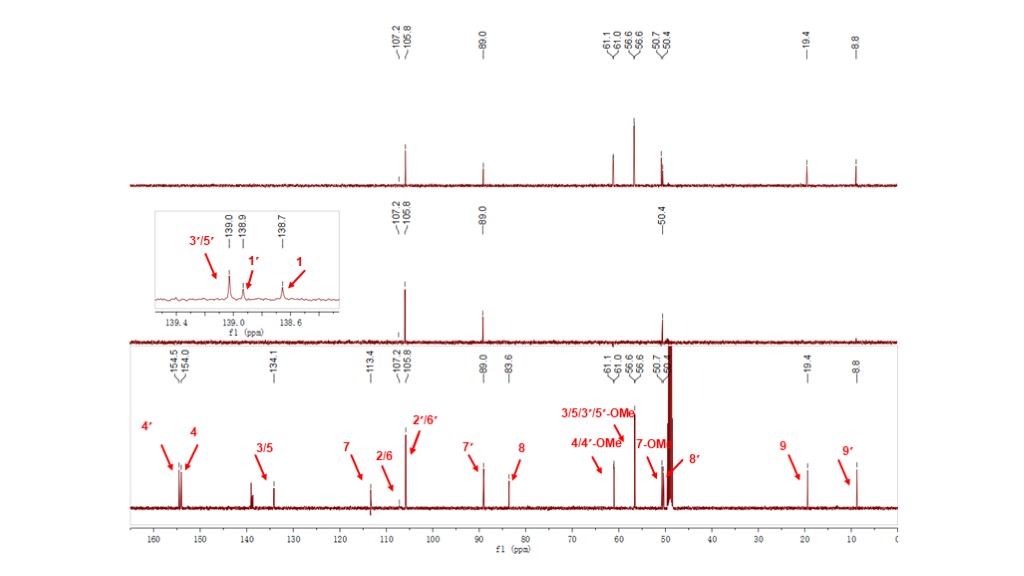

**Figure S2.** ^13^C NMR spectrum of compound (±)-**1** (methanol-*d*_4_).


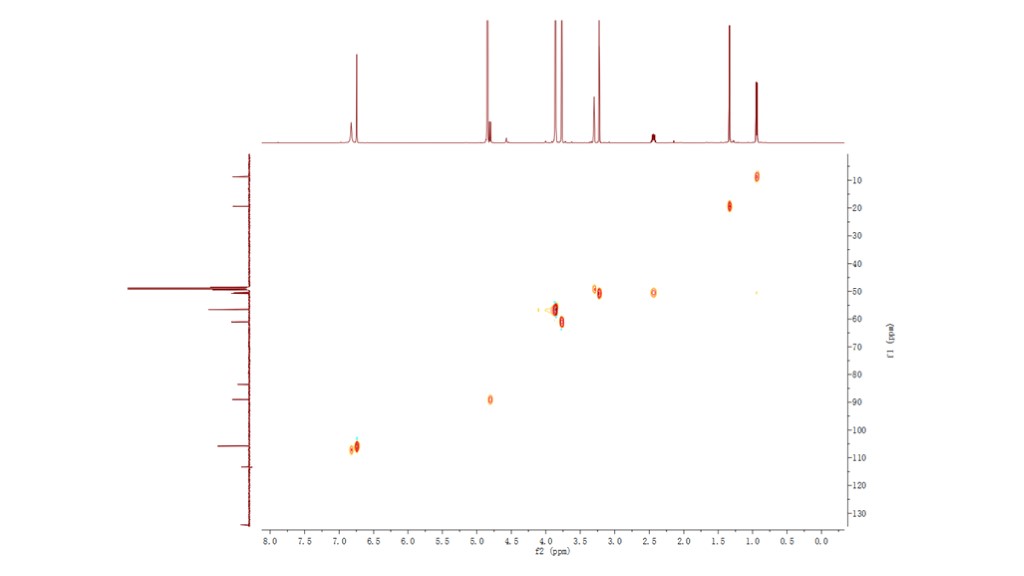

**Figure S3.** HMQC spectrum of compound (±)-**1** (methanol-*d*_4_).


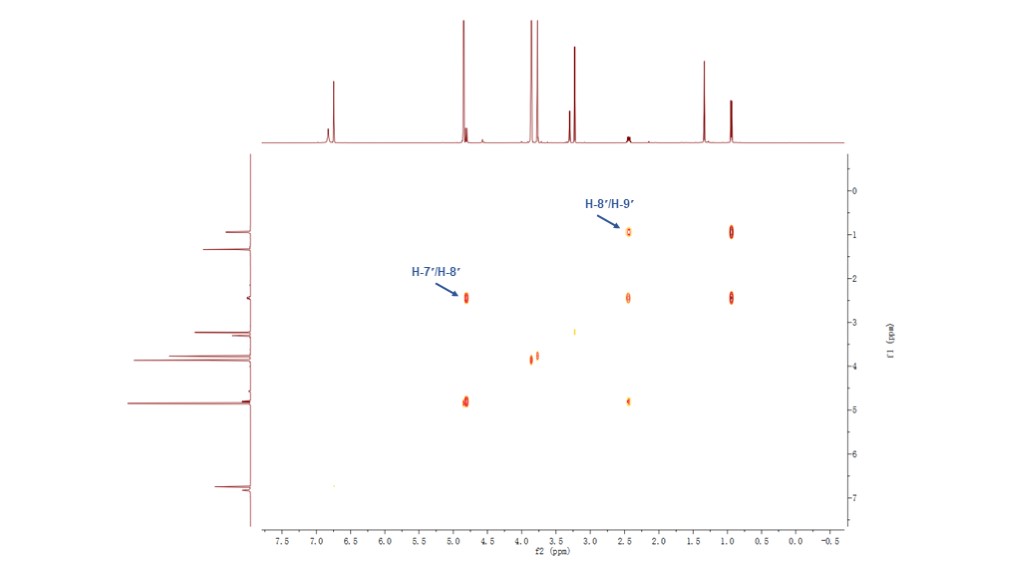

**Figure S4.** ^1^H-^1^H COSY spectrum of compound (±)-**1** (methanol-*d*_4_) and the selected cross-peaks (blue arrow and text).


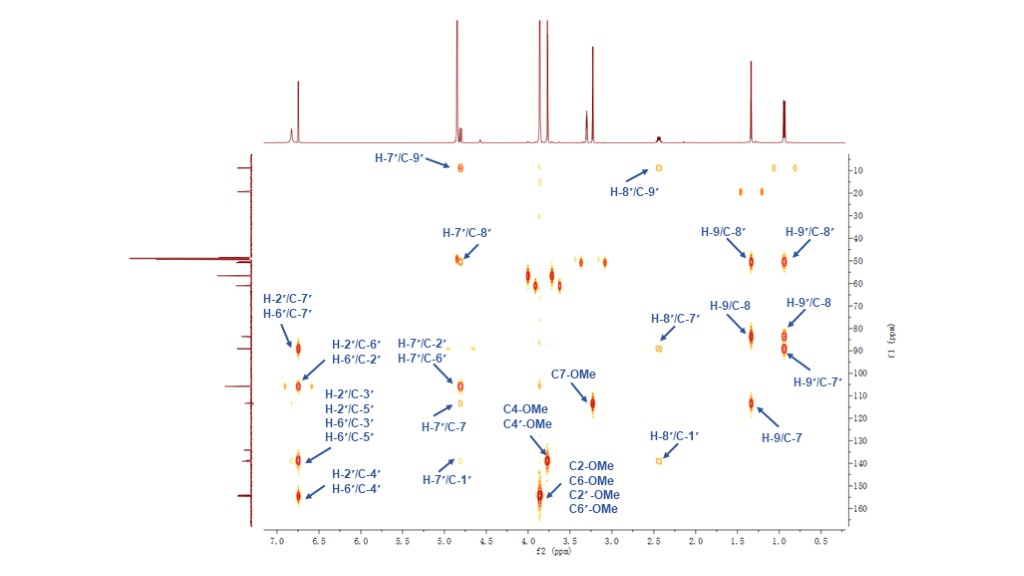

**Figure S5.** HMBC spectrum of compound (±)-**1** (methanol-*d*_4_) and the selected cross-peaks (blue arrow and text).


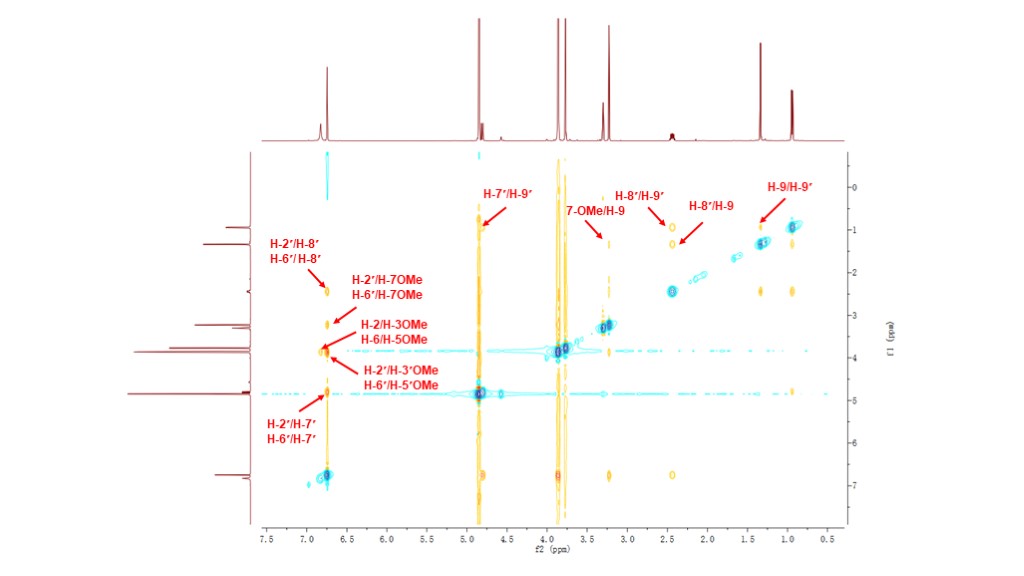

**Figure S6.** ROESY spectrum of compound (±)-**1** (methanol-*d*_4_) and the selected cross-peaks (red arrow and text).

**Figure S7.** IR spectrum of compound (±)-**1**.


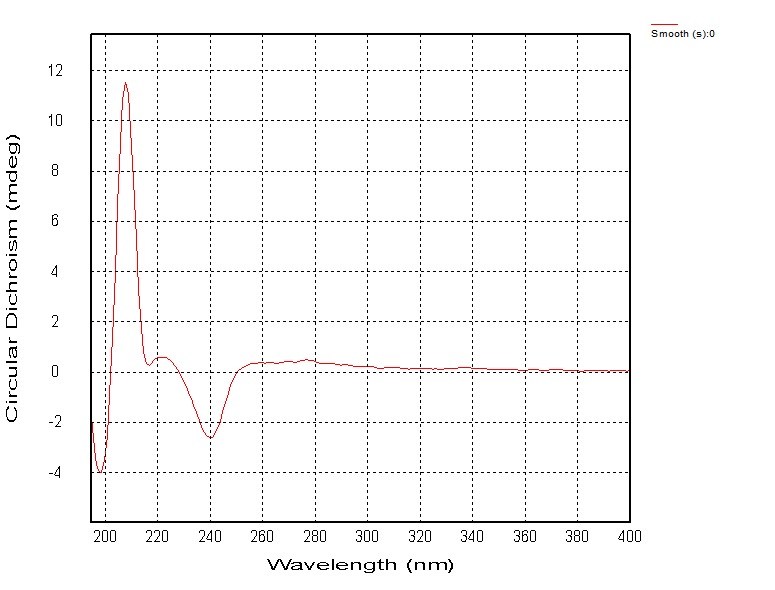


**Figure S8.** CD spectrum of compound (−)-**1**.


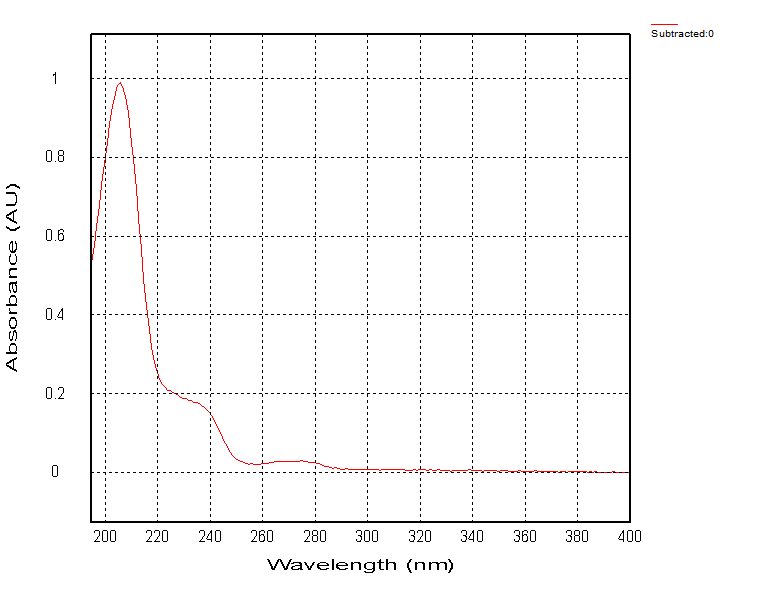


**Figure S9.** UV spectrum of compound (−)-**1**.


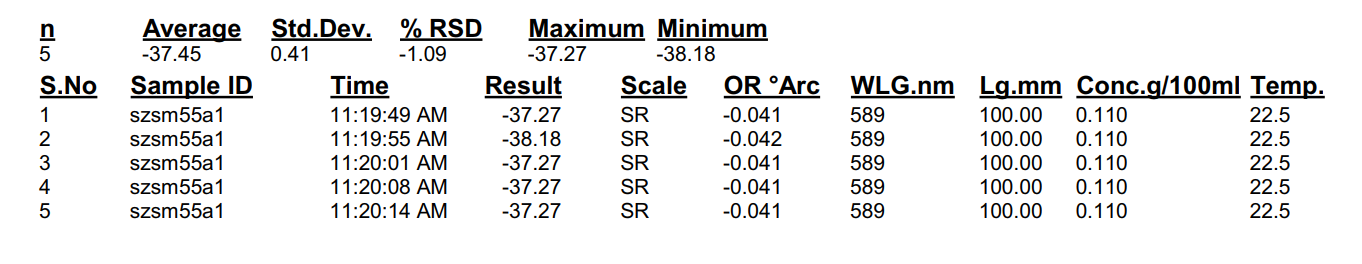


**Figure S10.** OR report of compound (−)-**1**.


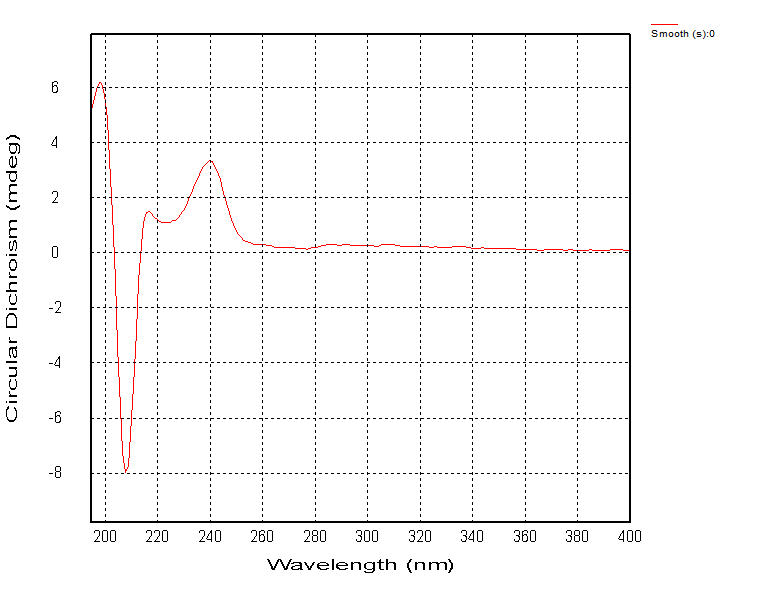


**Figure S11.** CD spectrum of compound (+)-**1**.


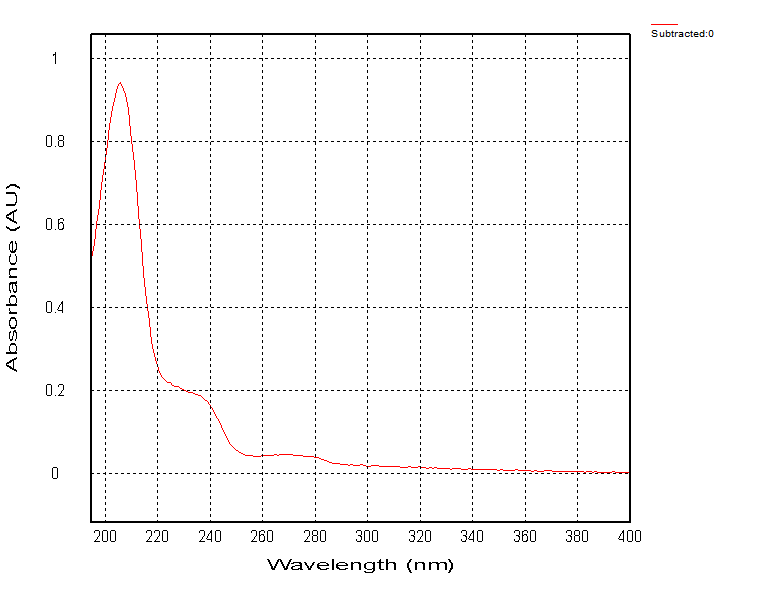


**Figure S12.** UV spectrum of compound (+)-**1**.


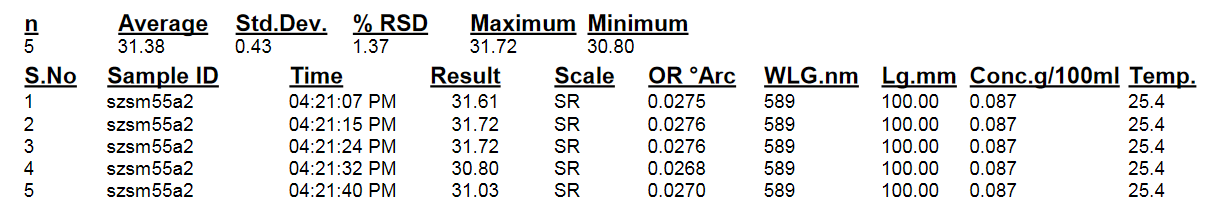


**Figure S13.** OR report of compound (+)-**1**.


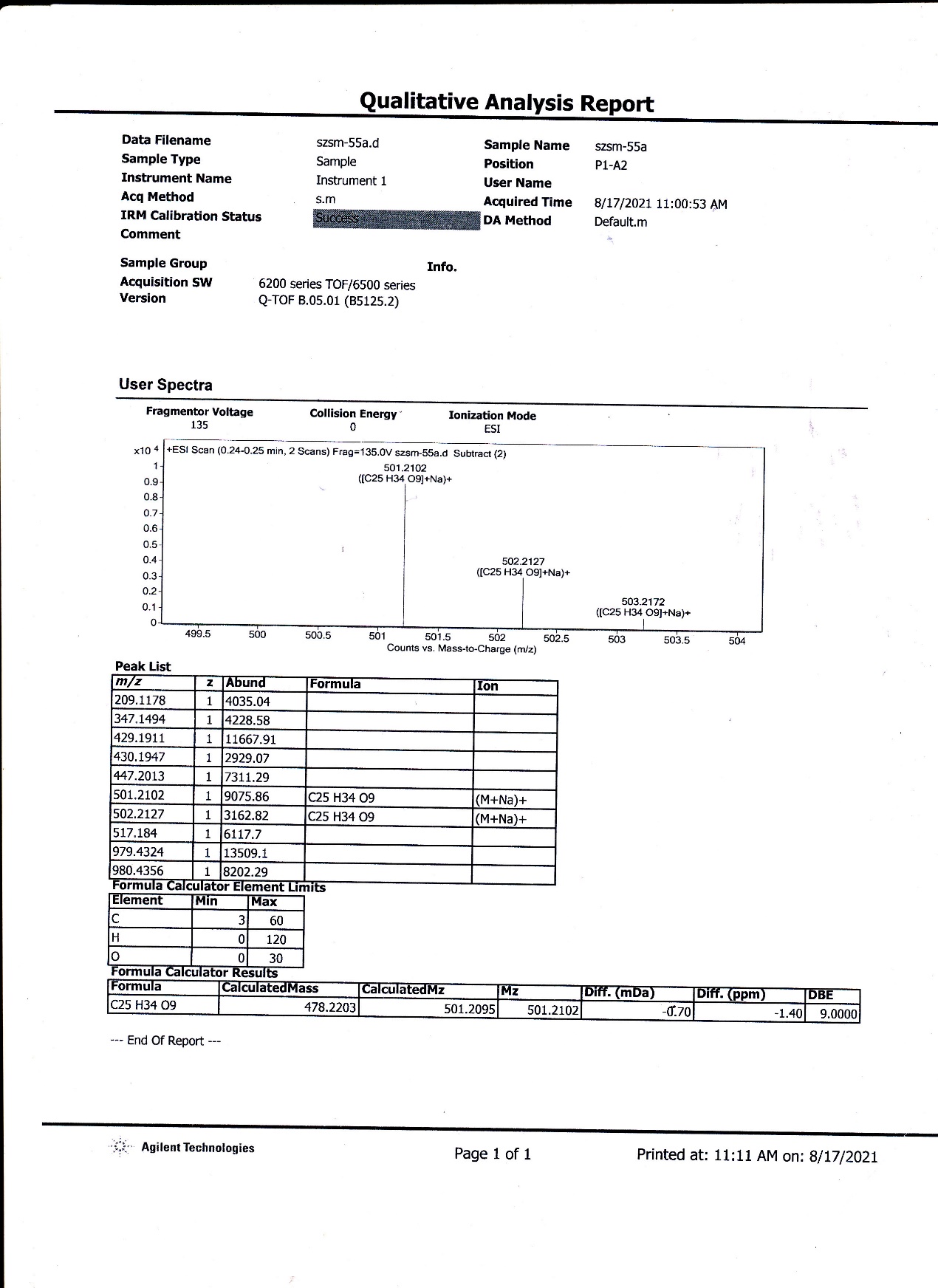


**Figure S14.** HRESIMS spectrum of compound (±)-**1**.


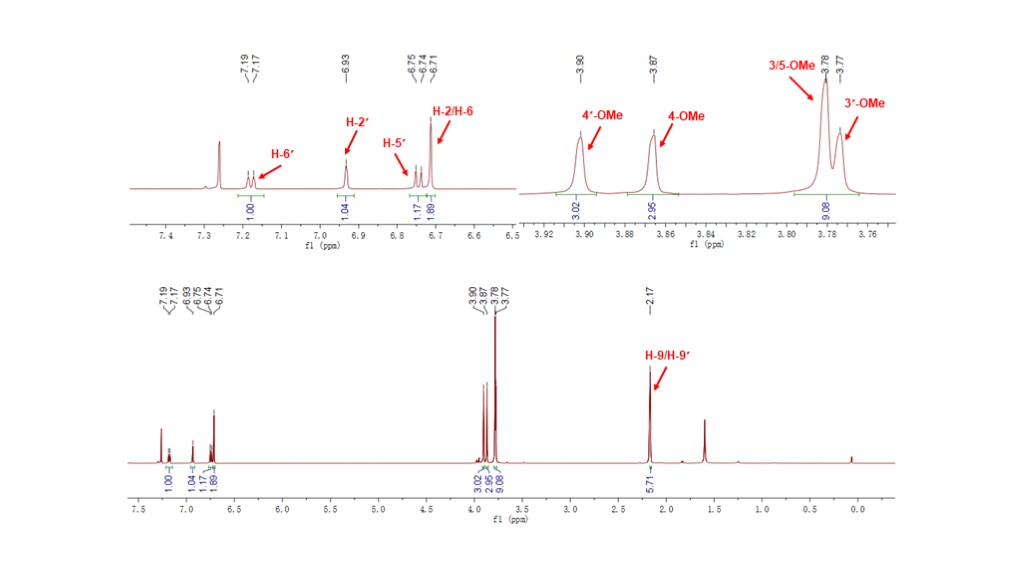

**Figure S15.** ^1^H NMR spectrum of compound **2** (chloroform-*d*).


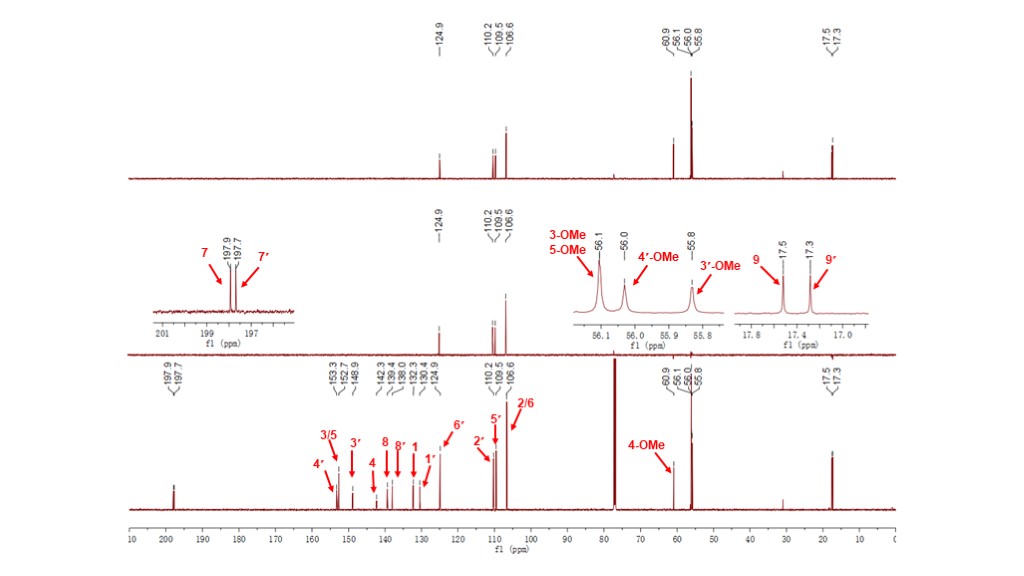

**Figure S16.** ^13^C NMR spectrum of compound **2** (chloroform-*d*).


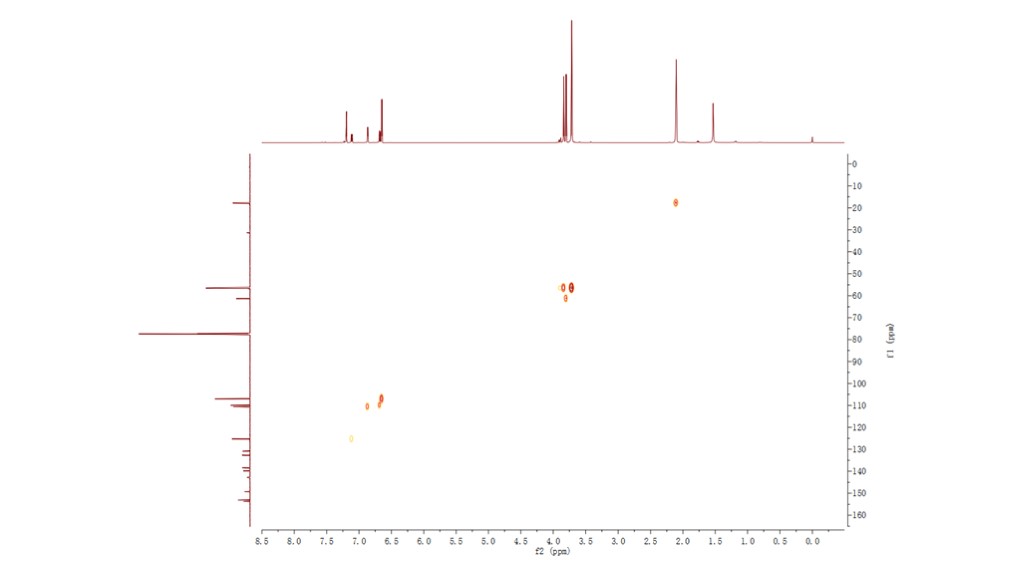

**Figure S17.** HMQC spectrum of compound **2** (chloroform-*d*).
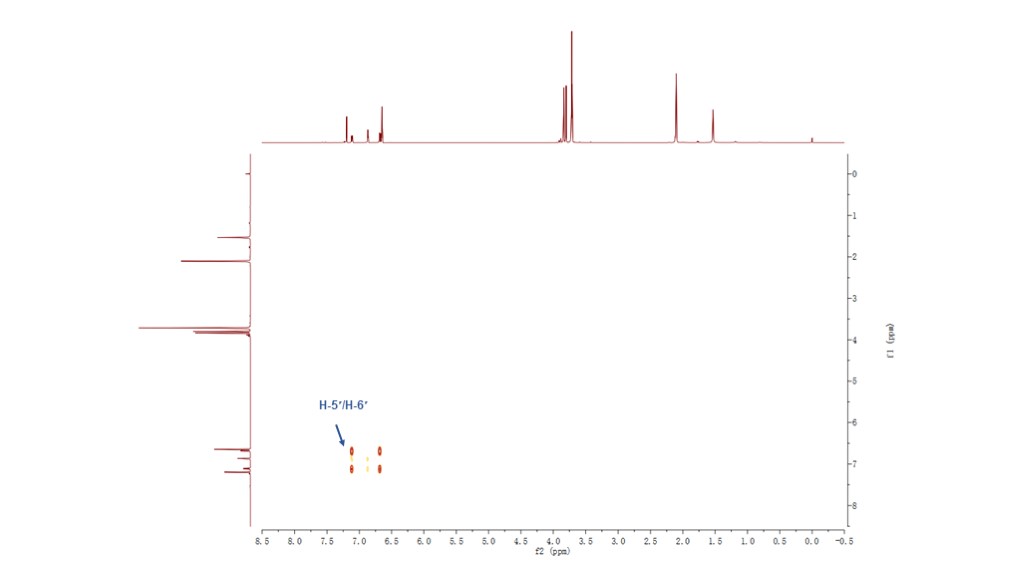

**Figure S18.** ^1^H-^1^H COSY spectrum of compound **2** (chloroform-*d*) and the selected cross-peaks (blue arrow and text).


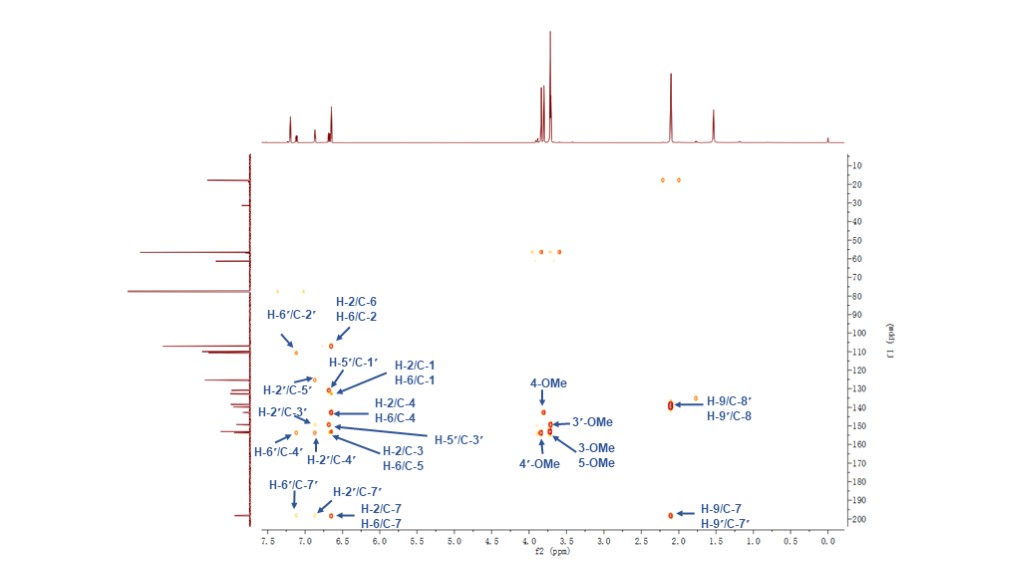

**Figure S19.** HMBC spectrum of compound **2** (chloroform-*d*) and the selected cross-peaks (blue arrow and text).


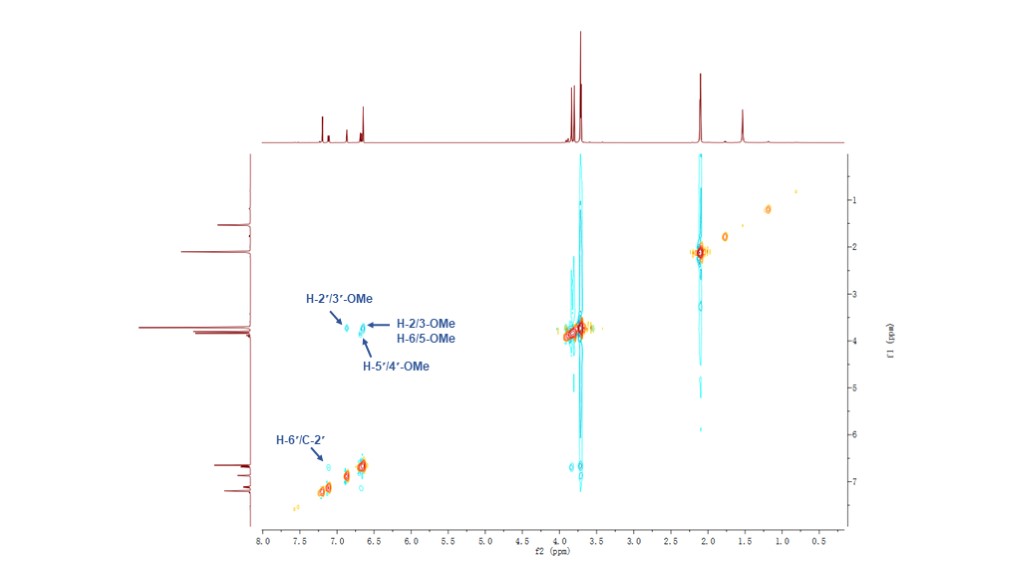

**Figure S20.** ROESY spectrum of compound **2** (chloroform-*d*) and the selected cross-peaks (blue arrow and text).

**Figure S21.** IR spectrum of compound **2**


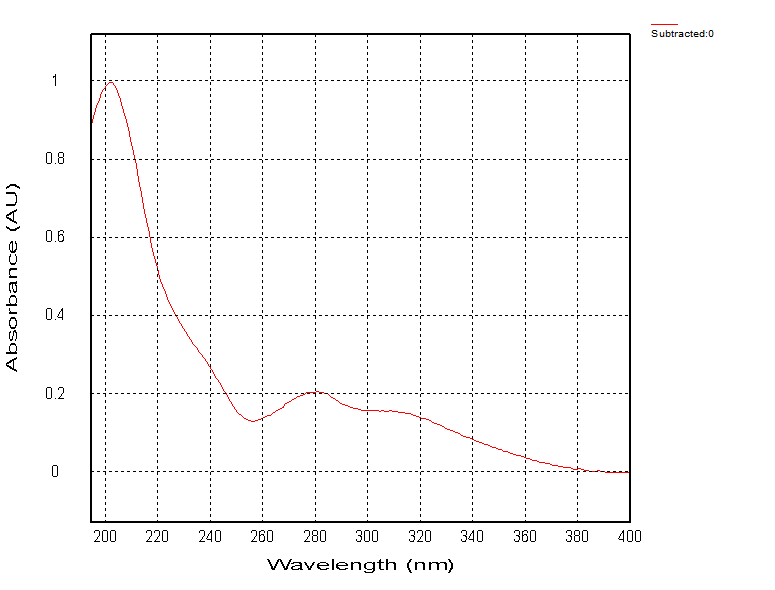


**Figure S22.** UV spectrum of compound **2**.


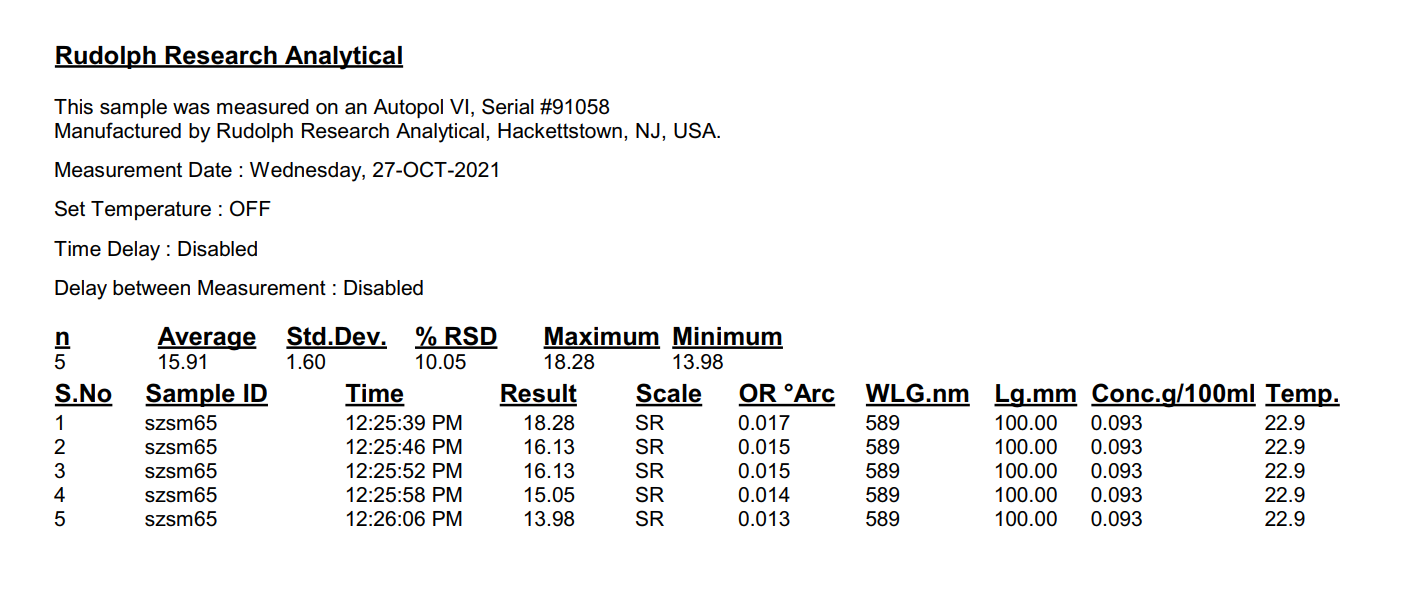


**Figure S23.** OR report of compound **2**.


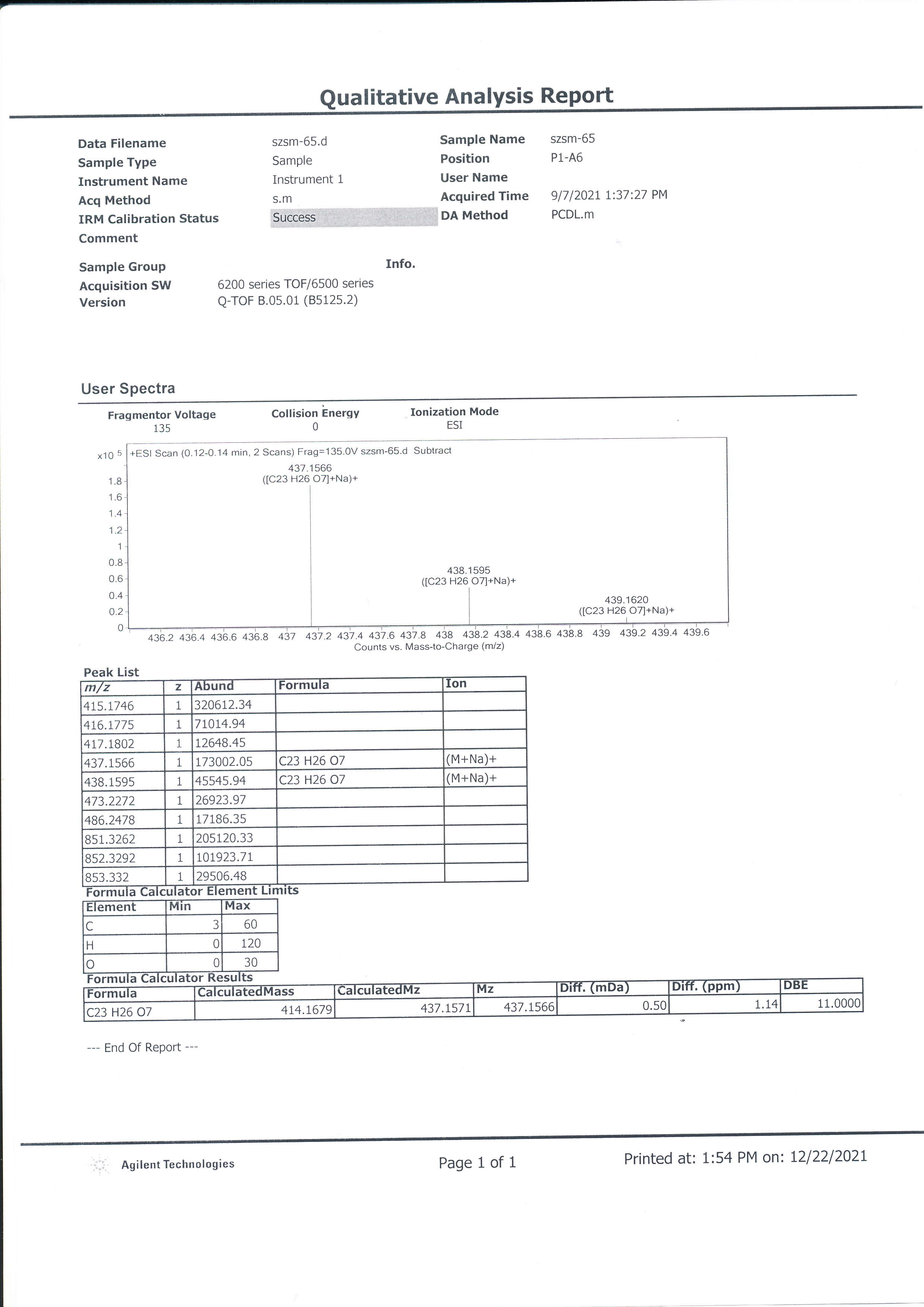


**Figure S24.** HRESIMS spectrum of compound **2**.


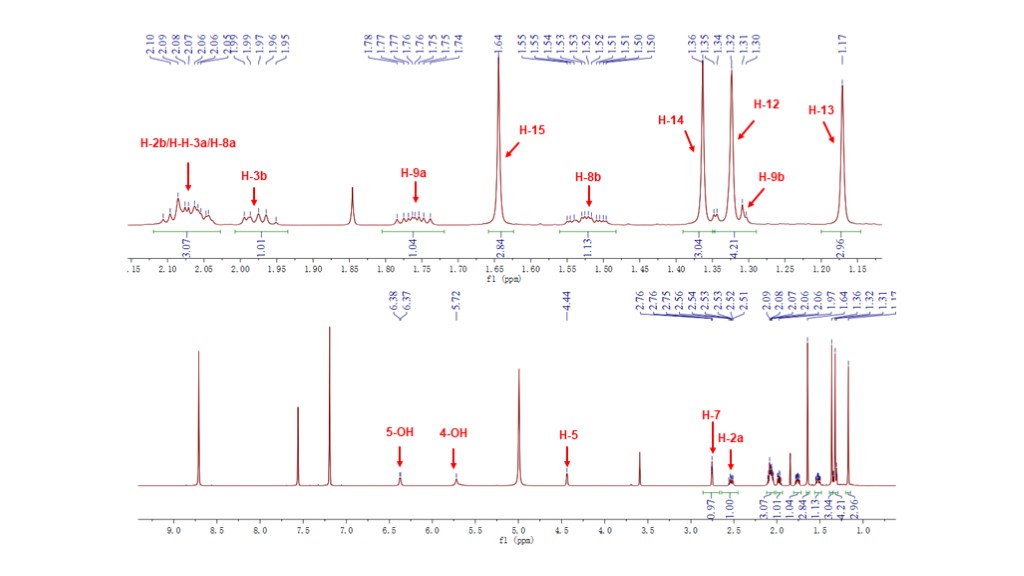

**Figure S25.** ^1^H NMR spectrum of compound **3** (pydine-*d*_5_).


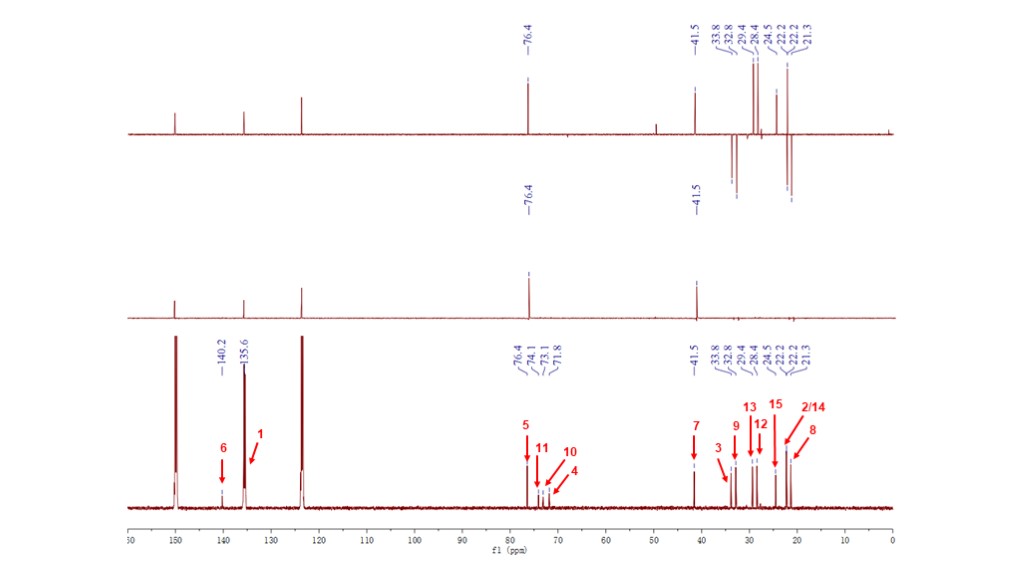

**Figure S26.** ^13^C NMR spectrum of compound **3** (pydine-*d*_5_).


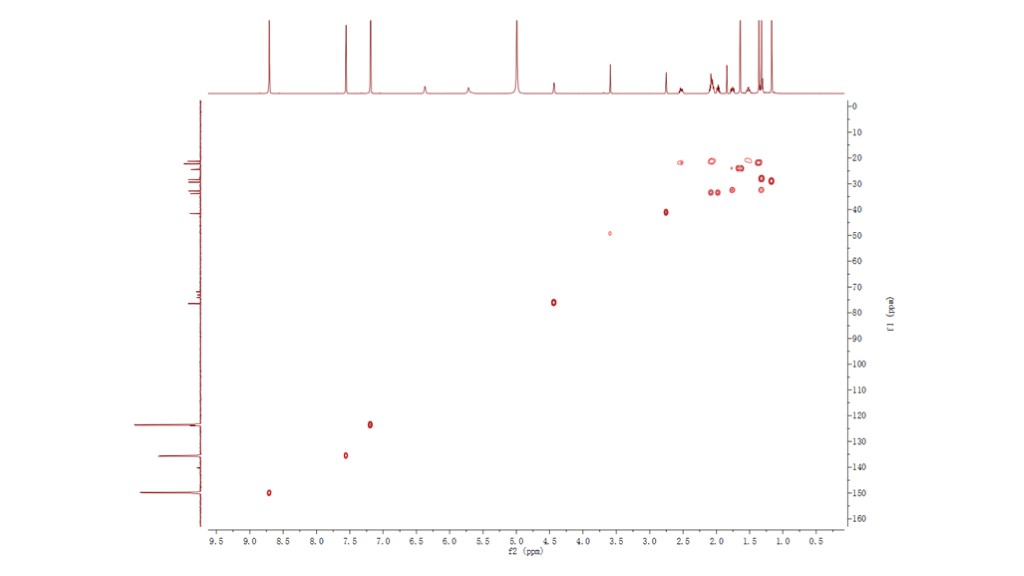

**Figure S27.** HSQC spectrum of compound **3** (pydine-*d*_5_).


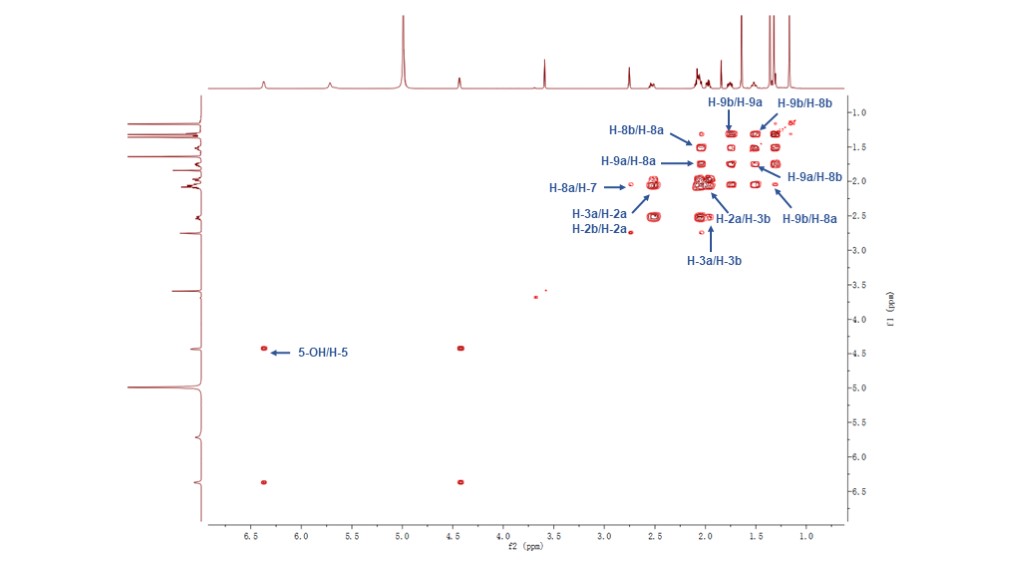

**Figure S28.** ^1^H-^1^H COSY spectrum of compound **3** (pydine-*d*_5_) and the selected cross-peaks (blue arrow and text).


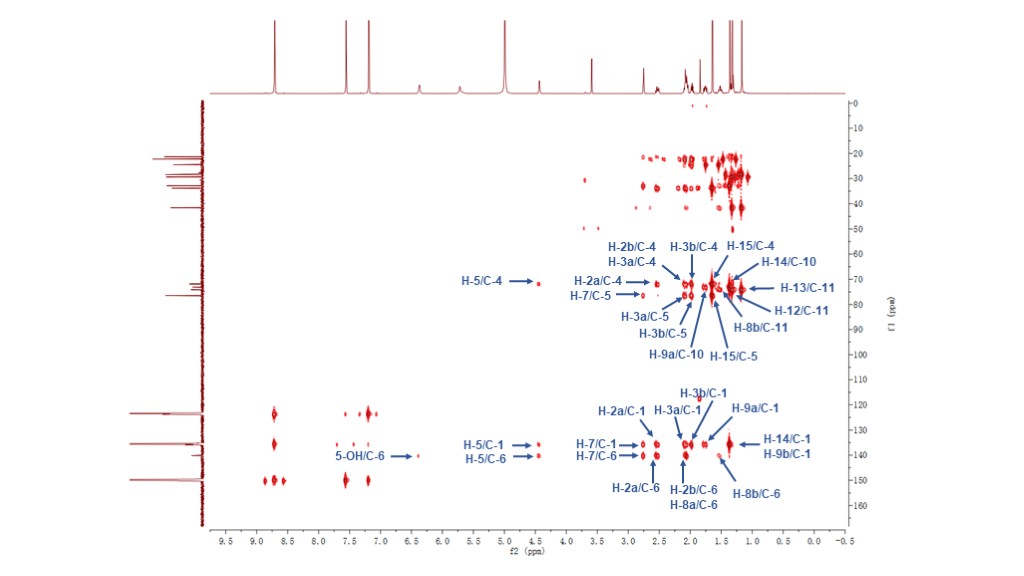

**Figure S29.** HMBC spectrum of compound **3** (pydine-*d*_5_) and the selected correlations (blue arrow and text).


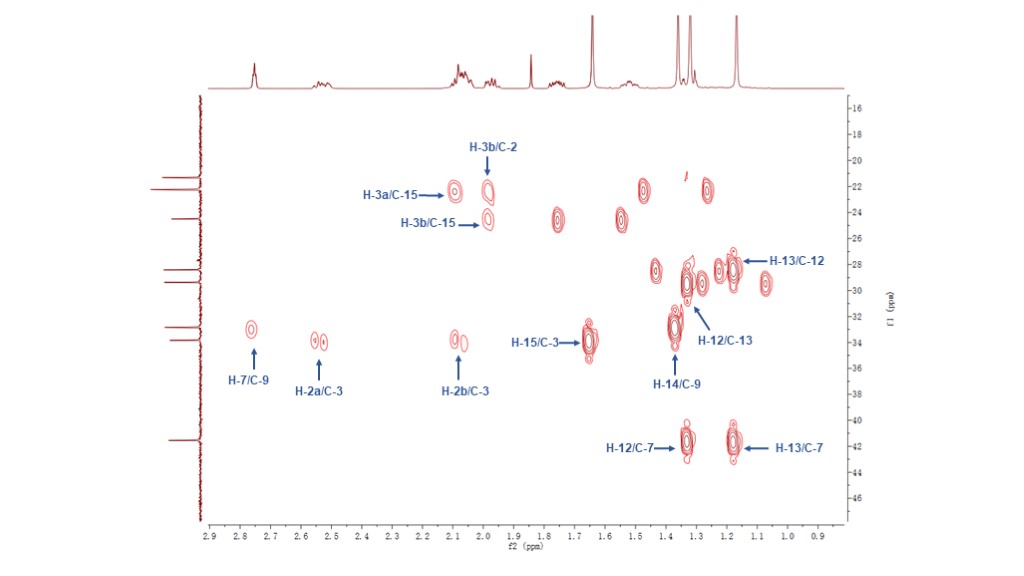

**Figure S30.** HMBC spectrum of compound **3** (pydine-*d*_5_) and the selected correlations (blue arrow and text).


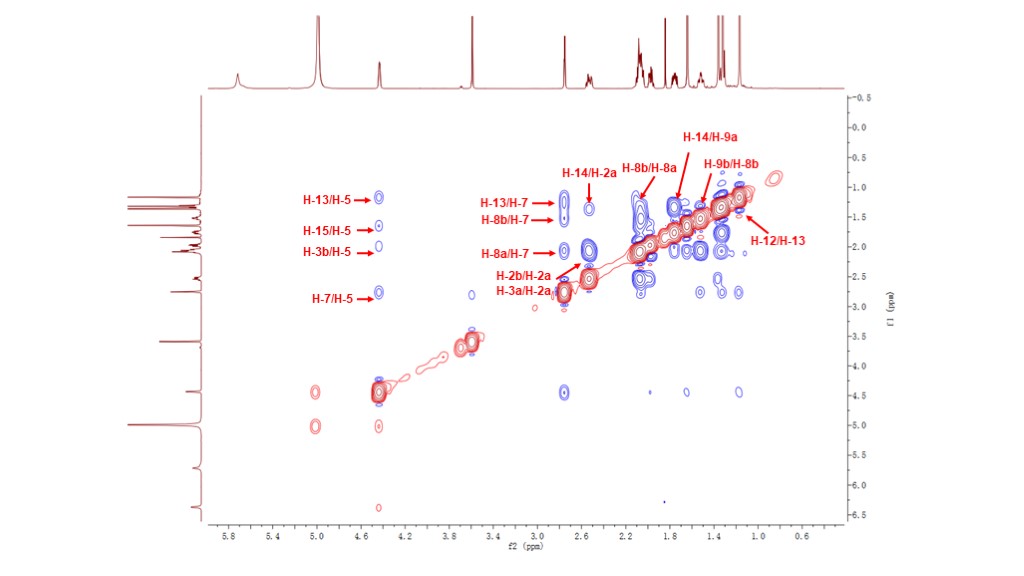

**Figure S31.** ROSY spectrum of compound **3** (pydine-*d*_5_) and the selected correlations (red arrow and text).

**Figure S32.** IR spectrum of compound **3**.


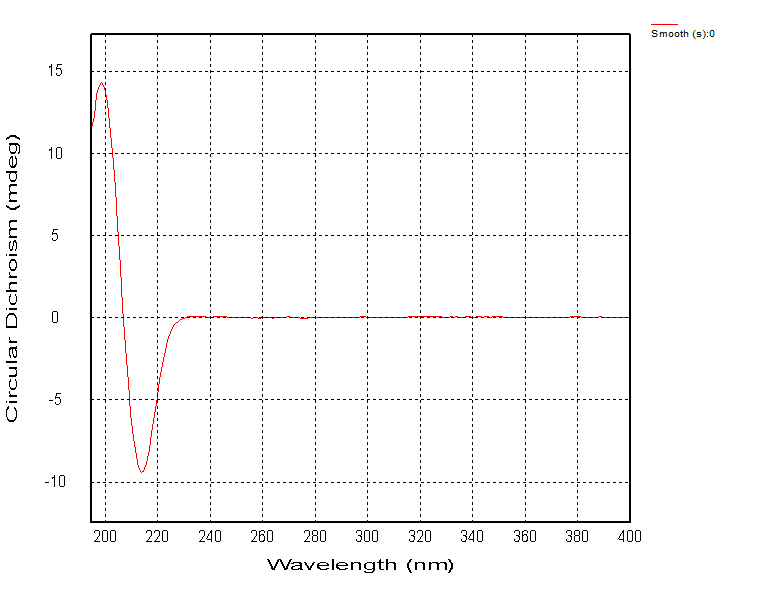


**Figure S33.** CD spectrum of compound **3**.


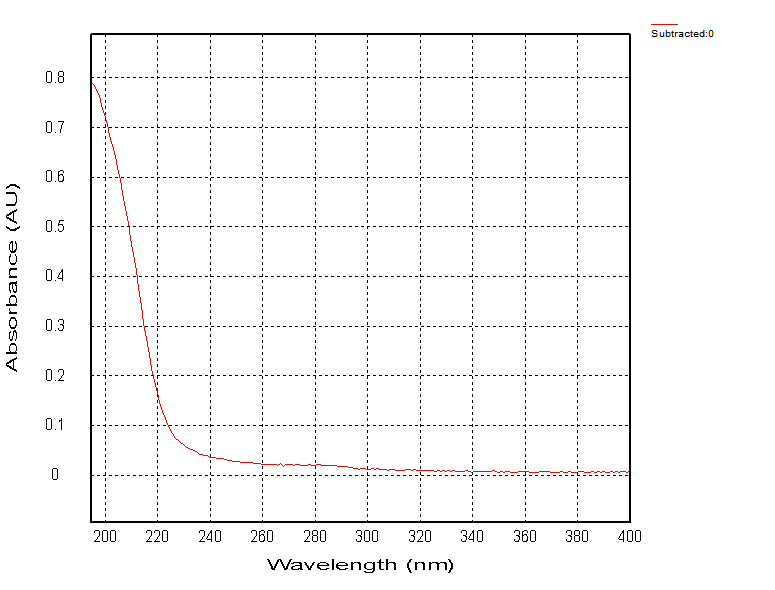


**Figure S34.** UV spectrum of compound **3**.


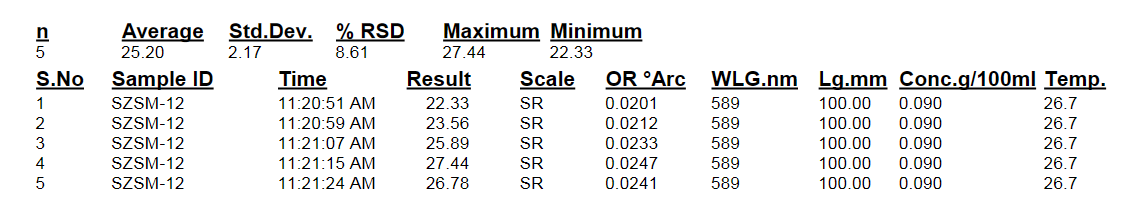


**Figure S35.** OR report of compound **3**.


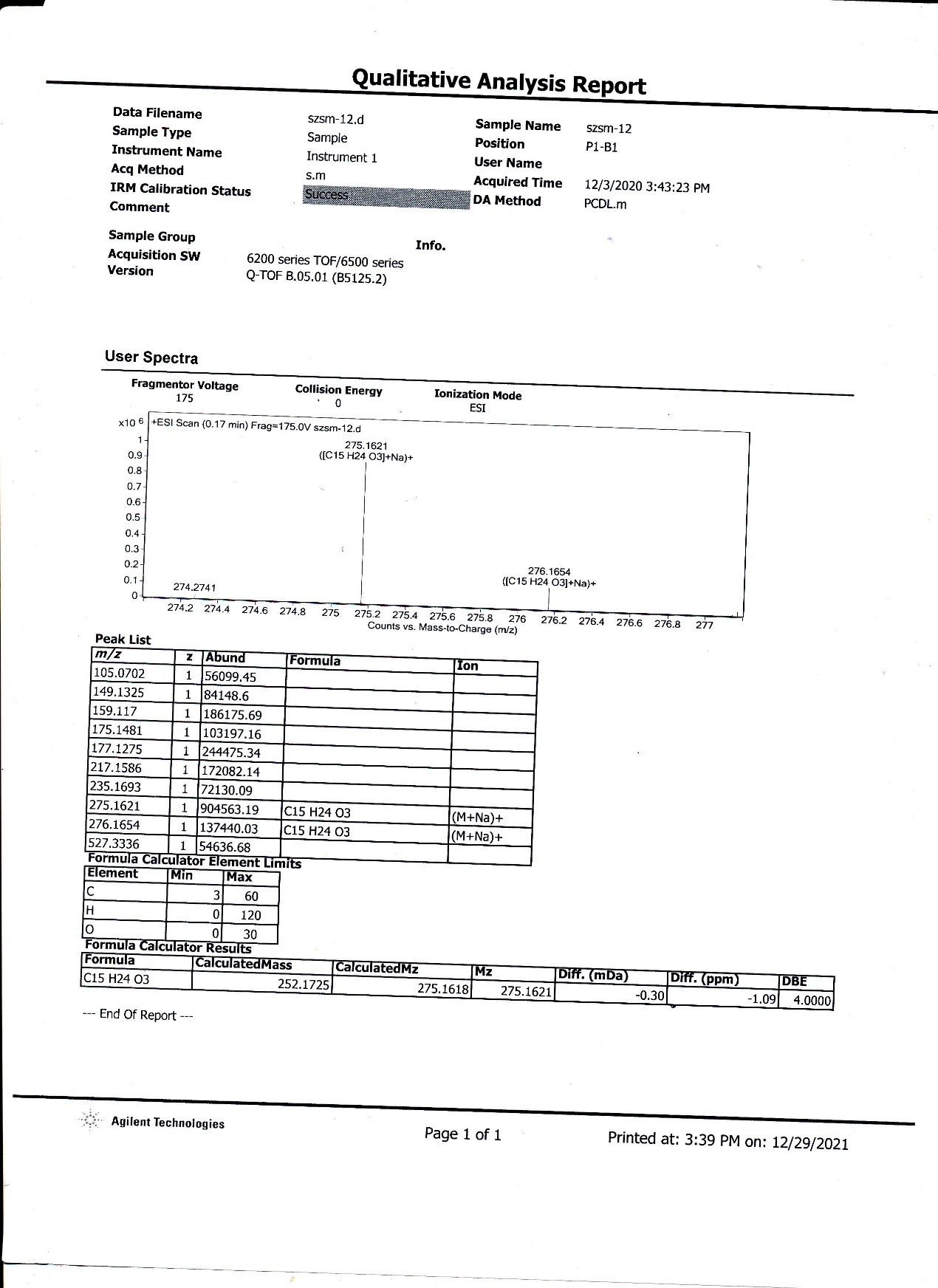


**Figure S36.** HRESIMS spectrum of compound **3**.


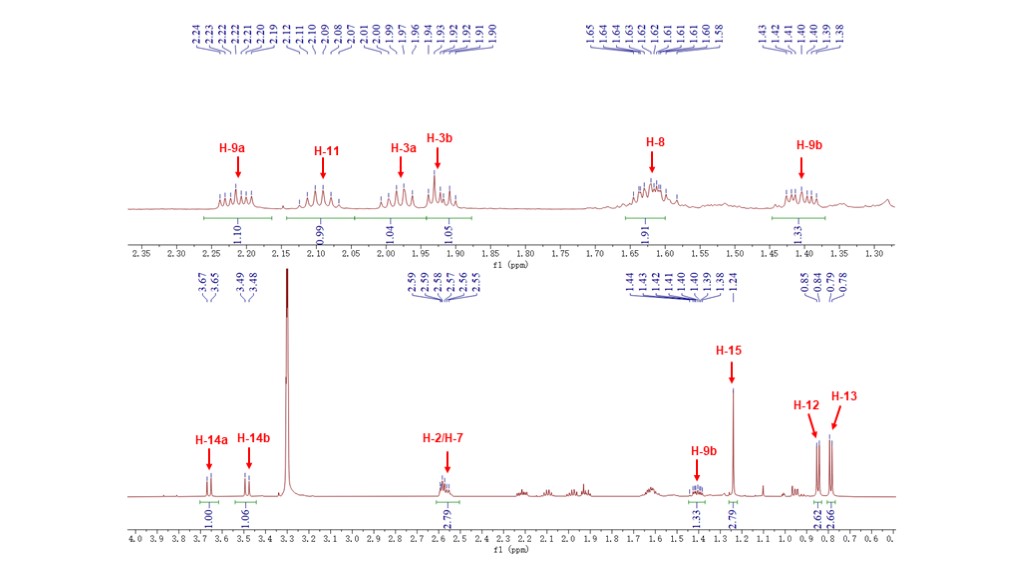

**Figure S37.** ^1^H NMR spectrum of compound **4** (methanol-*d*_4_).
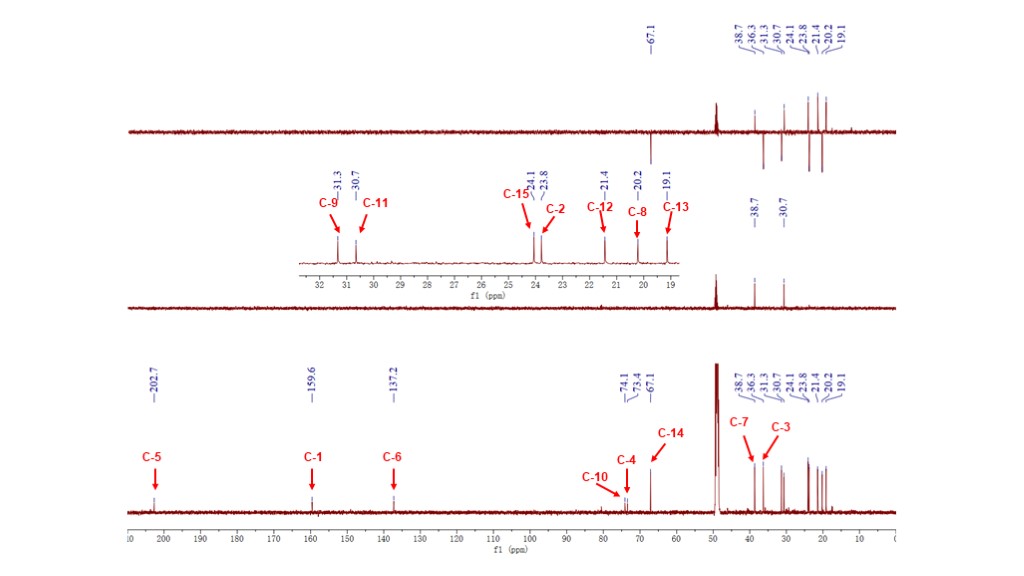

**Figure S38.** ^13^C NMR spectrum of compound **4** (methanol-*d*_4_).


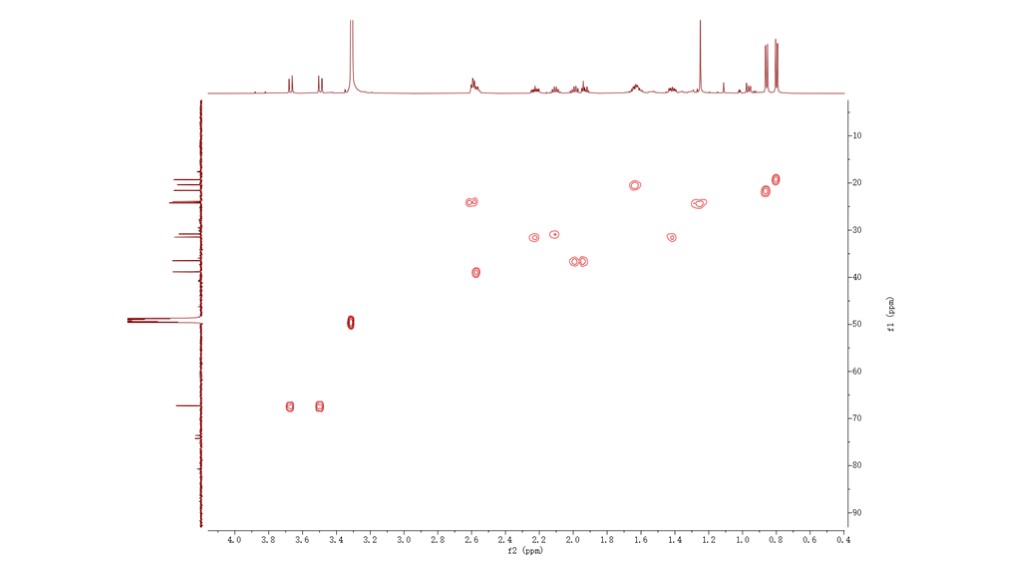

**Figure S39.** HMQC spectrum of compound **4** (methanol-*d*_4_).


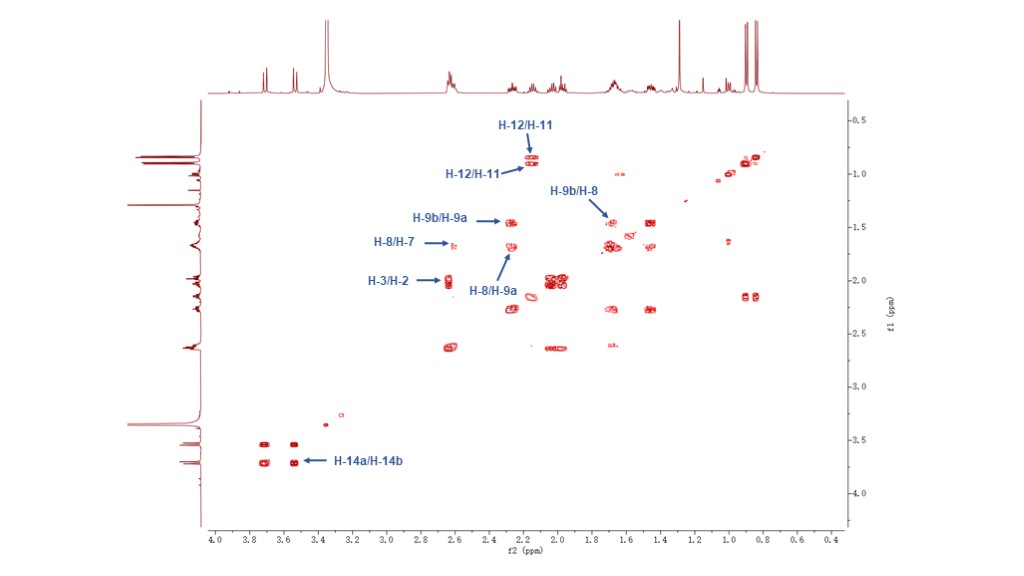

**Figure S40.** ^1^H-^1^H COSY spectrum of compound **4** (methanol-*d*_4_) and the selected cross-peaks (blue arrow and text).


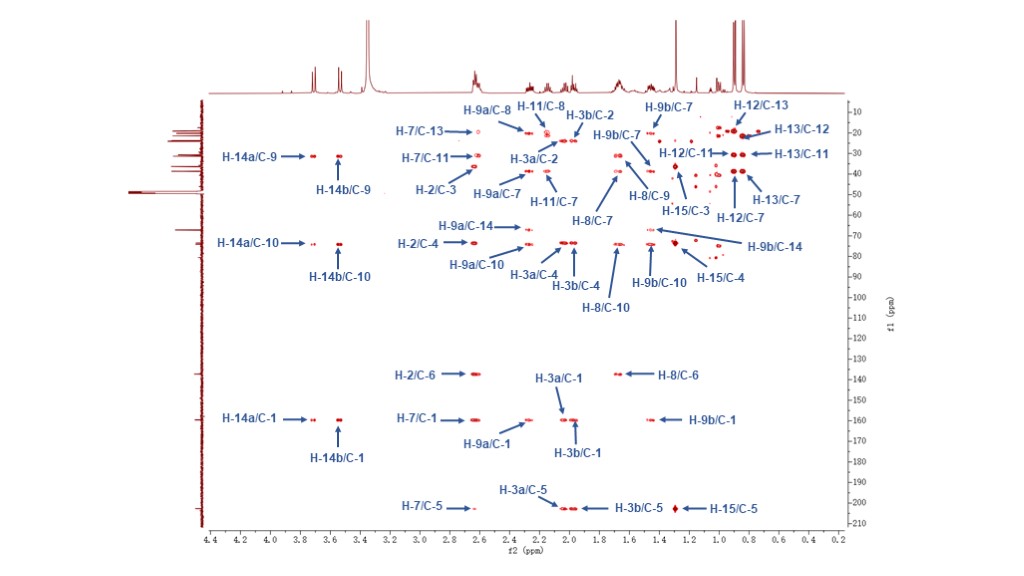

**Figure S41.** HMBC spectrum of compound **4** (methanol-*d*_4_) and the selected cross-peaks (blue arrow and text).


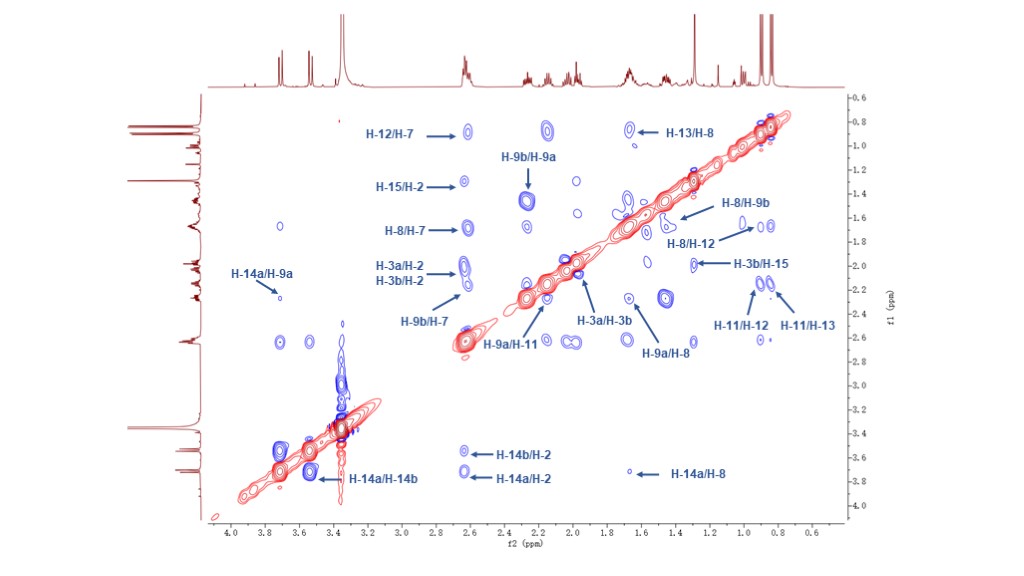

**Figure S42.** ROESY spectrum of compound **4** (methanol-*d*_4_) and the selected cross-peaks (blue arrow and text).

**Figure S43.** IR spectrum of compound **4**.


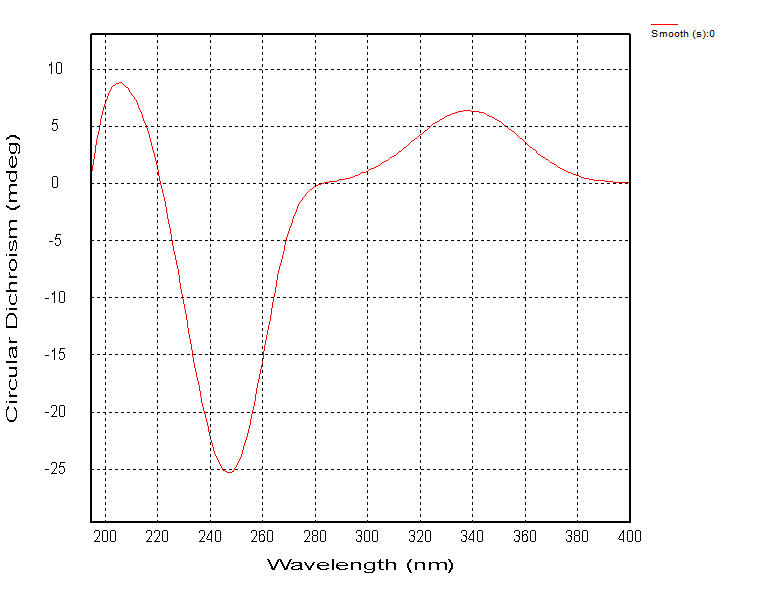


**Figure S44.** CD spectrum of compound **4**.


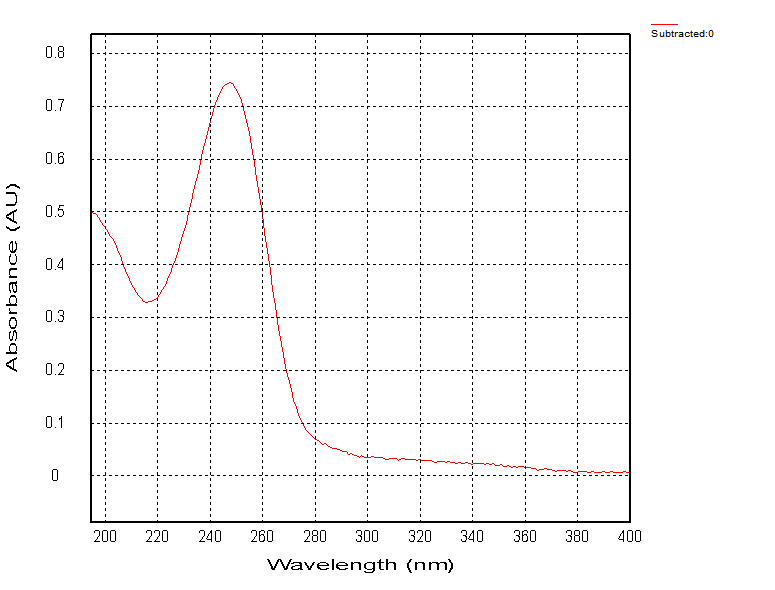


**Figure S45.** UV spectrum of compound **4**


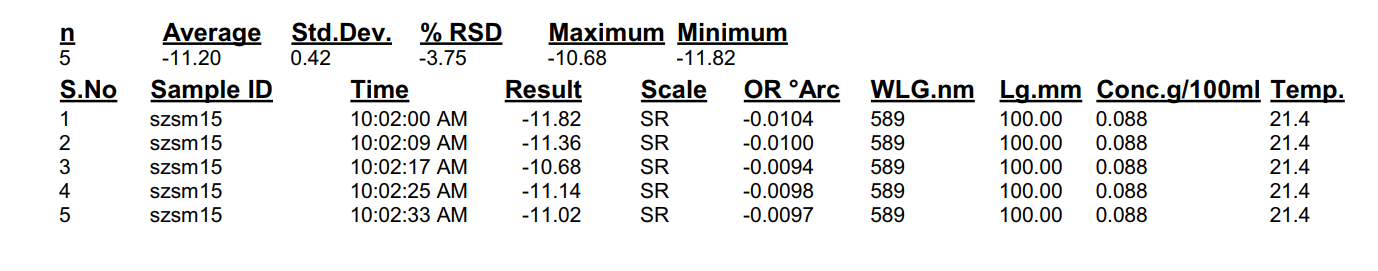


**Figure S46.** OR report of compound **4**.


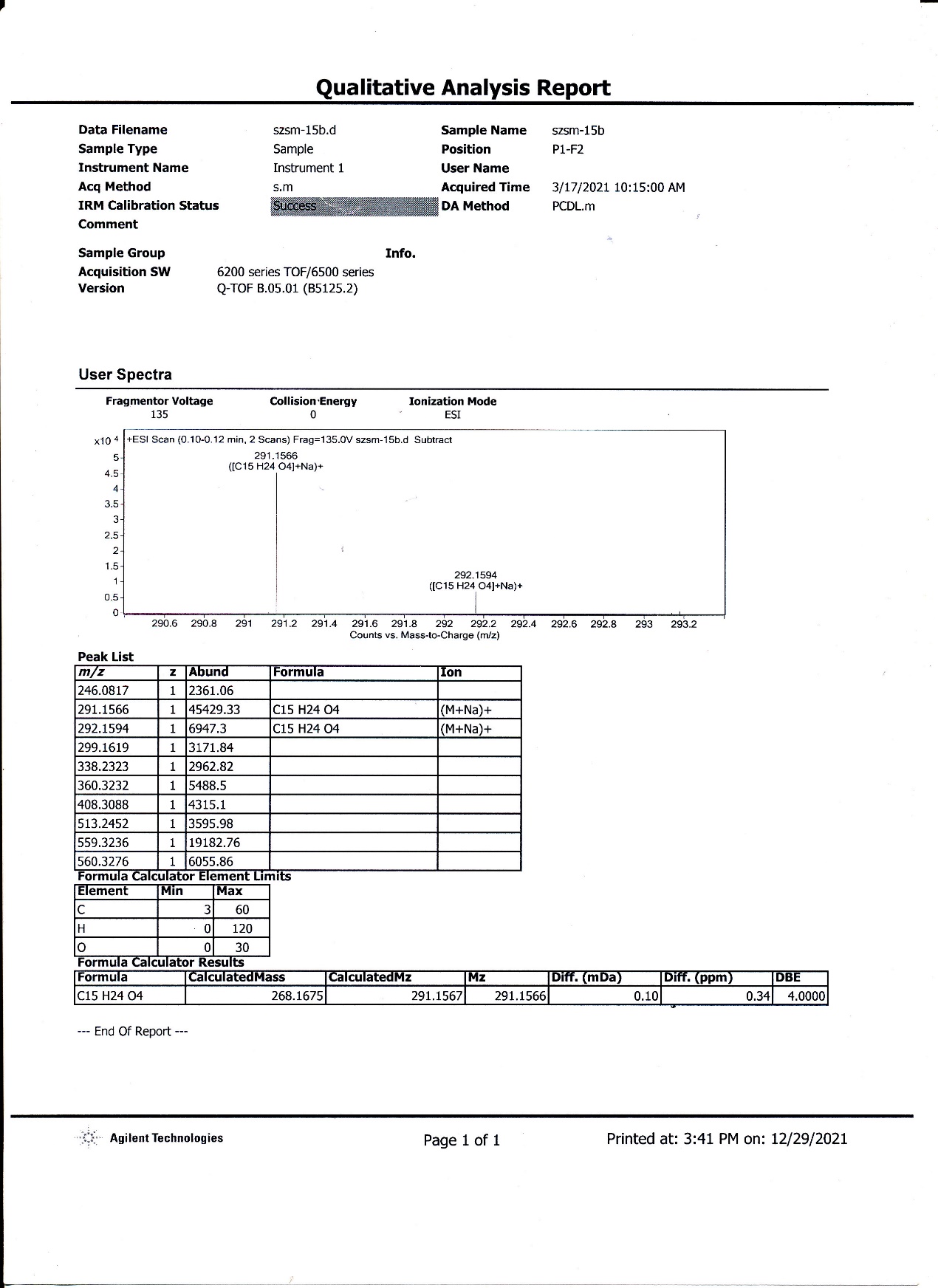


**Figure S47.** HRESIMS spectrum of compound **4**.

| **Methods for quantum chemical calculations**  Conformational searching of (7*S*,8*S*,7′*S*,8′*R*)-**1** (**1a**), **2a** and **2b**, **3**, **4a**–**4d** were performed with the Crest code (version 2.11) using the default iMTD-GC procedure [1]. Those two conformers with difference in distance matrix below 0.5 Å were regarded as duplicate conformers, and the one with higher energy was removed. After clustering, the first 25 conformers of each isomer were subjected to DFT geometry optimizations at B3LYP-D3BJ/6-31G(d) level of theory in the gas phase. Frequency analysis of all optimized conformations was undertaken at the same level of theory to ensure they were true local minima on the potential energy surface. Then, energies of all optimized conformations were evaluated at M06-2X-D3/6-311+G(2d,p) level of theory. Gibbs free energy of each conformer was calculated by adding thermal correction to Gibbs free energy obtained by frequency analysis to electronic energies obtained at M06-2X-D3/6-311+G(2d,p) level of theory. Room-temperature (298.15 K) equilibrium populations were calculated according to Boltzmann distribution law:  $p_{i}= \frac{n_{i}}{\sum_{j} n_{j}}= \frac{e^{-\Delta G_{i}/RT}}{\sum_{j} e^{-\Delta G_{j}/RT}}$  Where *P_i_* is the population of the *i^th^* conformer; *n_i_* the number of molecules in *i^th^* conformer; *ΔG* is the relative Gibbs free energy (kcal/mol); *T* is room temperature (298.15 K) here; *R* is the ideal gas constant (0.0019858995). Those conformers with a population of over 2% were subjected to subsequent NMR and ECD calculations.  NMR shielding constants were calculated with the GIAO method at mPW1PW91-SCRF/6-31+G(d,p) level with IEFPCM solvent model in DMSO solvent. The shielding constants obtained were converted into chemical shifts by referencing to TMS at 0 ppm (*δ*cal = *σ*TMS – *σ*cal), where the *σ*TMS was the shielding constant of TMS calculated at the same level. For each possible candidate, the parameters *a* and *b* of the linear regression *δ*cal = *aδ*exp + *b*; the correlation coefficient, *R*^2^; the mean absolute error (MAE) defined as Σn \|*δ*cal – *δ*exp\|/*n*; the corrected mean absolute error, CMAE, defined as Σn \|*δ*corr –*δ*exp\|/*n*, where *δ*corr = (*δ*cal – *b*)/*a*, were calculated. Then, DP4+ probability analysis [2] were undertaken using the calculated NMR chemical shifts and EXCEL spreadsheet provided by Sarotti, *et al*, and DP4+ probabilities of each structural candidates were obtained.  TDDFT ECD calculation of each conformer was run at CAM-B3LYP/6-31+G(2d,p) level of theory (MeOH, IEFPCM solvent model), and 36 excited states were calculated for each conformer. The calculated ECD curves were generated using the Multiwfn software (version 3.8) [3].  The geometry optimization, single-point energy calculation, NMR shielding constant calculation were all completed in Gaussian 09 program [4].  [1] P. Pracht, F. Bohle, S. Grimme, Automated exploration of the low-energy chemical space with fast quantum chemical methods, *Phys. Chem. Chem. Phys.* 22 (**2020**) 7169–7192.  [2] N. Grimblat; M. M. Zanardi; A. M. Sarotti, Beyond DP4: an improved probability for the stereochemical assignment of isomeric compounds using quantum chemical calculations of NMR Shifts, *J. Org. Chem.* 80 (**2015**) 12526−12534.  [3] T. Lu, F. Chen, Multiwfn: A multifunctional wavefunction analyzer, J. Comput. Chem. 33 (2012) 580–592.  [4] M.J. Frisch, G.W. Trucks, H.B. Schlegel, G.E. Scuseria, M.A. Robb, J.R. Cheeseman, G. Scalmani, V. Barone, B. Mennucci, G.A. Petersson, H. Nakatsuji, M. Caricato, X. Li, H.P. Hratchian, A.F. Izmaylov, J. Bloino, G. Zheng, J.L. Sonnenberg, M. Hada, M. Ehara, K. Toyota, R. Fukuda, J. Hasegawa, M. Ishida, T. Nakajima, Y. Honda, O. Kitao, H. Nakai, T. Vreven, J.A. Montgomery, J.E.P. Jr., F. Ogliaro, M. Bearpark, J.J. Heyd, E. Brothers, K.N. Kudin, V.N. Staroverov, T. Keith, R. Kobayashi, J. Normand, K. Raghavachari, A. Rendell, J.C. Burant, S.S. Iyengar, J. Tomasi, M. Cossi, N. Rega, J.M. Millam, M. Klene, J.E. Knox, J.B. Cross, V. Bakken, C. Adamo, J. Jaramillo, R. Gomperts, R.E. Stratmann, O. Yazyev, A.J. Austin, R. Cammi, C. Pomelli, J.W. Ochterski, R.L. Martin, K. Morokuma, V.G. Zakrzewski, G.A. Voth, P. Salvador, J.J. Dannenberg, S. Dapprich, A.D. Daniels, O. Farkas, J.B. Foresman, J.V. Ortiz, J. Cioslowski, D.J. Fox, Gaussian 09, Revision E.01; Gaussian, Inc., Wallingford CT: **2010**.  Computational data of **1** |
| --- |

**Table S1.** Conformational analysis of the B3LYP-D3BJ/6-31G(d) optimized conformers of **1a** in the gas phase (T=298.15 K)

| Conformer | E (Hartree)*^a^* | C (Hartree)*^b^* | G (kcal/mol)*^c^* | *Δ*G (kcal/mol)*^d^* | Population*^e^* |
| --- | --- | --- | --- | --- | --- |
| **1a**-1 | -1649.914554 | 0.487138 | -1035015.703295 | 0.0 | 42.11% |
| **1a**-2 | -1649.914724 | 0.487731 | -1035015.437938 | 0.265357 | 26.90% |
| **1a**-3 | -1649.914772 | 0.488225 | -1035015.158161 | 0.545134 | 16.77% |
| **1a**-4 | -1649.913057 | 0.486911 | -1035014.906508 | 0.796787 | 10.96% |
| **1a**-5 | -1649.91225 | 0.487253 | -1035014.185894 | 1.517402 | 3.25% |

*^a^*Electronic energy obtained at M06-2X-D3/6-311+G(2d,p) level of theory; *^b^*Thermal correction to Gibbs free energy obtained at B3LYP-D3BJ/6-31G(d) level of theory; *^c^*Gibbs free energy (E + C); *^d^*The relative Gibbs free energy; *^e^*The Boltzmann distribution of each conformer.

**Table S2.** Atomic coordinates (Å) of **1a**-1 obtained at the B3LYP-D3BJ/6-31G(d) level of theory in the gas phase.

| C | -2.243288 | -0.193410 | -0.079443 | H | 0.745105 | 1.317749 | 3.679683 |
| --- | --- | --- | --- | --- | --- | --- | --- |
| C | -2.506684 | 1.115655 | -0.486202 | H | 2.312234 | 0.502016 | 3.467841 |
| C | -3.794444 | 1.461123 | -0.915019 | H | 0.998550 | -0.310162 | 4.333008 |
| C | -4.813808 | 0.496226 | -0.940895 | O | 5.727730 | 2.109276 | -0.057068 |
| C | -4.542270 | -0.811444 | -0.505059 | H | -6.550285 | 2.248050 | 0.015528 |
| C | -3.256057 | -1.157928 | -0.078035 | H | -2.337578 | 3.451822 | -2.015354 |
| C | -0.873296 | -0.558289 | 0.465602 | H | -2.753888 | 3.892058 | -0.333331 |
| C | -0.768905 | -0.402757 | 2.019667 | H | -3.653349 | 4.618097 | -1.696700 |
| C | -1.383473 | -1.544908 | 2.815912 | H | -7.885778 | 1.540880 | -0.938175 |
| C | 2.493058 | 0.378085 | 0.409442 | O | -1.345378 | 0.845533 | 2.403317 |
| C | 3.435334 | 1.405425 | 0.437069 | H | -5.048809 | -3.056608 | 0.923394 |
| C | 4.738125 | 1.171666 | -0.024812 | H | -4.629030 | -3.499679 | -0.757067 |
| C | 5.095687 | -0.096181 | -0.504974 | H | -6.335622 | -3.514654 | -0.228945 |
| C | 4.131491 | -1.118723 | -0.547526 | H | -7.119571 | 0.581581 | 0.360278 |
| C | 2.836576 | -0.885036 | -0.081342 | H | -3.019812 | -2.163596 | 0.241961 |
| C | 1.107054 | 0.619606 | 0.950668 | C | -0.385598 | -2.181170 | -1.224189 |
| C | 0.749321 | -0.243355 | 2.174631 | H | -0.023127 | -3.209831 | -1.293641 |
| C | 1.226768 | 0.354281 | 3.492972 | H | 0.339766 | -1.499285 | -1.678469 |
| C | -3.159793 | 3.713649 | -1.337593 | H | -1.341726 | -2.095821 | -1.754057 |
| C | -6.944728 | 1.325273 | -0.427205 | H | -1.696599 | 1.830806 | -0.479257 |
| C | -5.376554 | -3.005115 | -0.123404 | H | -1.227612 | -1.367731 | 3.884525 |
| C | 5.424863 | 3.402400 | 0.435856 | H | -0.942996 | -2.505297 | 2.539400 |
| C | 6.597062 | -0.161660 | -2.302927 | H | -2.463452 | -1.594655 | 2.636881 |
| C | 3.632941 | -3.372298 | -1.113345 | O | 6.384425 | -0.341650 | -0.903695 |
| H | 2.087151 | -1.664986 | -0.090122 | H | 3.144920 | 2.380684 | 0.809318 |
| O | -5.601759 | -1.666989 | -0.534351 | H | 0.994086 | 1.678759 | 1.208914 |
| O | -4.160385 | 2.707496 | -1.323554 | O | -0.535979 | -1.909372 | 0.167501 |
| O | -6.056166 | 0.815946 | -1.421141 | H | 4.622668 | 3.878996 | -0.143378 |
| H | 6.383114 | 0.872193 | -2.602036 | H | 6.342015 | 3.984372 | 0.330089 |
| H | 5.975272 | -0.851178 | -2.886769 | H | 5.133394 | 3.372320 | 1.493997 |
| H | 7.651873 | -0.381048 | -2.484389 | H | 4.169146 | -4.216462 | -1.550167 |
| H | -2.235957 | 0.886580 | 2.016580 | H | 2.767496 | -3.123325 | -1.741663 |
| H | 1.193847 | -1.234384 | 2.040672 | H | 3.278382 | -3.644381 | -0.110642 |
| O | 4.564029 | -2.305089 | -1.063198 | O | 0.099720 | 0.323528 | -0.049934 |

**Table S3.** Atomic coordinates (Å) of **1a**-2 obtained at the B3LYP-D3BJ/6-31G(d) level of theory in the gas phase.

| C | -2.289553 | -0.157252 | 0.088705 | H | 1.263018 | -0.394237 | 4.248084 |
| --- | --- | --- | --- | --- | --- | --- | --- |
| C | -3.299858 | -1.123009 | 0.136729 | H | 0.958564 | 1.250016 | 3.661028 |
| C | -4.613662 | -0.767526 | -0.186846 | H | 2.509530 | 0.448940 | 3.315439 |
| C | -4.921935 | 0.559380 | -0.530774 | O | 4.422439 | -2.197884 | -1.485004 |
| C | -3.896650 | 1.517091 | -0.584132 | H | -6.434736 | -0.094009 | -2.599508 |
| C | -2.580459 | 1.161272 | -0.264902 | H | -5.026271 | -3.054684 | 1.188682 |
| C | -0.883909 | -0.535544 | 0.521528 | H | -4.697184 | -3.454209 | -0.522615 |
| C | -0.666791 | -0.424583 | 2.067087 | H | -6.372407 | -3.491825 | 0.098462 |
| C | -1.222294 | -1.590077 | 2.873069 | H | -7.611088 | 1.199698 | -2.230187 |
| C | 2.468621 | 0.417384 | 0.247255 | O | -1.211925 | 0.811508 | 2.527119 |
| C | 2.770740 | -0.820828 | -0.327170 | H | -2.831147 | 3.928272 | -0.014255 |
| C | 4.024925 | -1.029818 | -0.903723 | H | -3.808573 | 4.691892 | -1.300602 |
| C | 4.983209 | -0.001054 | -0.908472 | H | -2.507093 | 3.544497 | -1.730079 |
| C | 4.681352 | 1.227952 | -0.304877 | H | -5.948956 | 1.628221 | -2.728181 |
| C | 3.416446 | 1.439986 | 0.260966 | H | -1.769891 | 1.874589 | -0.308245 |
| C | 1.125115 | 0.634249 | 0.895346 | C | -0.523517 | -2.109549 | -1.244848 |
| C | 0.859362 | -0.266293 | 2.115779 | H | -0.161025 | -3.132849 | -1.370755 |
| C | 1.429009 | 0.294957 | 3.413434 | H | 0.160749 | -1.409444 | -1.733826 |
| C | -5.411233 | -2.979424 | 0.163327 | H | -1.517603 | -2.017790 | -1.698720 |
| C | -6.560013 | 0.908342 | -2.171002 | H | -3.038922 | -2.138895 | 0.400323 |
| C | -3.291439 | 3.779569 | -0.999291 | H | -0.991813 | -1.441437 | 3.932497 |
| C | 3.479300 | -3.254264 | -1.536543 | H | -0.800981 | -2.541302 | 2.540399 |
| C | 7.190643 | -0.752172 | -0.679988 | H | -2.312146 | -1.638512 | 2.769464 |
| C | 5.423474 | 3.420197 | 0.246745 | O | 6.192860 | -0.185384 | -1.527374 |
| H | 3.162177 | 2.391970 | 0.711576 | H | 2.023604 | -1.602796 | -0.309268 |
| O | -4.285705 | 2.767690 | -0.958180 | H | 1.027326 | 1.684617 | 1.192683 |
| O | -5.665472 | -1.632504 | -0.198469 | O | -0.569586 | -1.877319 | 0.161103 |
| O | -6.220159 | 0.914570 | -0.784433 | H | 3.180994 | -3.580441 | -0.531251 |
| H | 6.888942 | -1.746527 | -0.328472 | H | 3.979074 | -4.076356 | -2.051687 |
| H | 7.390443 | -0.101095 | 0.180316 | H | 2.581577 | -2.963067 | -2.097178 |
| H | 8.095340 | -0.840322 | -1.286165 | H | 4.597121 | 3.930660 | -0.265037 |
| H | -2.133486 | 0.856554 | 2.222099 | H | 6.339763 | 3.999274 | 0.119755 |
| H | 1.293983 | -1.251345 | 1.919765 | H | 5.188291 | 3.341083 | 1.316646 |
| O | 5.684878 | 2.151068 | -0.325894 | O | 0.049860 | 0.361621 | -0.038319 |

**Table S4.** Atomic coordinates (Å) of **1a**-3 obtained at the B3LYP-D3BJ/6-31G(d) level of theory in the gas phase.

| C | -2.265792 | -0.153654 | 0.082948 | H | 2.424924 | 0.287230 | 3.491125 |
| --- | --- | --- | --- | --- | --- | --- | --- |
| C | -3.295537 | -1.099702 | 0.070170 | H | 1.130180 | -0.552400 | 4.358799 |
| C | -4.589731 | -0.709858 | -0.290707 | H | 0.877519 | 1.110470 | 3.800127 |
| C | -4.860026 | 0.631232 | -0.610743 | O | 5.737850 | 2.039699 | -0.046211 |
| C | -3.815343 | 1.569239 | -0.602898 | H | -6.317807 | 0.050873 | -2.741111 |
| C | -2.518609 | 1.178919 | -0.246819 | H | -4.713592 | -3.385799 | -0.695764 |
| C | -0.884178 | -0.569413 | 0.556909 | H | -6.410676 | -3.403775 | -0.136450 |
| C | -0.720636 | -0.495224 | 2.111211 | H | -5.096960 | -3.020425 | 1.011890 |
| C | -1.327278 | -1.665910 | 2.871284 | H | -7.473945 | 1.366893 | -2.386842 |
| C | 2.493070 | 0.328155 | 0.423548 | O | -1.258050 | 0.741922 | 2.578161 |
| C | 3.453038 | 1.337662 | 0.485151 | H | -3.643614 | 4.760399 | -1.226094 |
| C | 4.732906 | 1.117400 | -0.042048 | H | -2.347624 | 3.601874 | -1.641780 |
| C | 5.048783 | -0.117095 | -0.626971 | H | -2.727478 | 3.942226 | 0.071736 |
| C | 4.066704 | -1.120353 | -0.702399 | H | -5.785506 | 1.762446 | -2.819129 |
| C | 2.796389 | -0.902959 | -0.165217 | H | -1.693608 | 1.876769 | -0.242276 |
| C | 1.131047 | 0.553855 | 1.028147 | C | -0.490412 | -2.114797 | -1.227956 |
| C | 0.805629 | -0.368283 | 2.217493 | H | -0.146406 | -3.143478 | -1.361710 |
| C | 1.338948 | 0.152444 | 3.547170 | H | 0.227006 | -1.421173 | -1.677319 |
| C | -5.442642 | -2.912856 | -0.024500 | H | -1.464765 | -1.991299 | -1.715948 |
| C | -6.433102 | 1.047825 | -2.297492 | H | -3.064207 | -2.126562 | 0.318686 |
| C | -3.154060 | 3.830285 | -0.933165 | H | -1.131672 | -1.544503 | 3.941111 |
| C | 5.479842 | 3.296645 | 0.554207 | H | -0.913274 | -2.618264 | 2.532739 |
| C | 6.492364 | 0.017753 | -2.465929 | H | -2.413449 | -1.689713 | 2.728056 |
| C | 3.503967 | -3.315513 | -1.415896 | O | 6.313581 | -0.354957 | -1.100574 |
| H | 2.034498 | -1.670137 | -0.196166 | H | 3.193403 | 2.288949 | 0.934170 |
| O | -4.166790 | 2.836522 | -0.957665 | H | 1.039631 | 1.598951 | 1.345473 |
| O | -5.657002 | -1.552814 | -0.362017 | O | -0.583047 | -1.909592 | 0.179573 |
| O | -6.141186 | 1.019364 | -0.900441 | H | 4.666179 | 3.829679 | 0.044361 |
| H | 6.322493 | 1.092639 | -2.604319 | H | 6.403855 | 3.869165 | 0.456984 |
| H | 5.817948 | -0.549999 | -3.119084 | H | 5.227914 | 3.190749 | 1.617559 |
| H | 7.528260 | -0.221745 | -2.717453 | H | 2.618459 | -3.003728 | -1.985574 |
| H | -2.166265 | 0.813221 | 2.239915 | H | 3.185283 | -3.660889 | -0.423713 |
| H | 1.228467 | -1.356975 | 2.014341 | H | 4.005330 | -4.130986 | -1.939963 |
| O | 4.456695 | -2.270866 | -1.321867 | O | 0.086385 | 0.320038 | 0.049870 |

**Table S5.** Atomic coordinates (Å) of **1a**-4 obtained at the B3LYP-D3BJ/6-31G(d) level of theory in the gas phase.

| C | -2.221423 | -0.249076 | 0.037330 | H | 2.429002 | 0.093789 | 3.508082 |
| --- | --- | --- | --- | --- | --- | --- | --- |
| C | -3.242963 | -1.196557 | -0.039311 | H | 1.144910 | -0.817665 | 4.317279 |
| C | -4.534445 | -0.802507 | -0.397683 | H | 0.862885 | 0.867879 | 3.847864 |
| C | -4.817698 | 0.543528 | -0.658052 | O | 4.538666 | -2.170151 | -1.424743 |
| C | -3.784762 | 1.497653 | -0.572161 | H | -5.661402 | 1.840242 | -2.810132 |
| C | -2.492905 | 1.099915 | -0.219298 | H | -7.234543 | -2.536972 | 0.167752 |
| C | -0.836862 | -0.666914 | 0.500255 | H | -7.126387 | -0.754697 | 0.298040 |
| C | -0.687598 | -0.677127 | 2.057861 | H | -6.202253 | -1.797842 | 1.424654 |
| C | -1.276180 | -1.901365 | 2.743180 | H | -6.143161 | 0.113570 | -2.899072 |
| C | 2.522571 | 0.297454 | 0.446452 | O | -1.256135 | 0.520297 | 2.588695 |
| C | 2.848425 | -0.893200 | -0.209410 | H | -2.327060 | 3.585247 | -1.497935 |
| C | 4.126115 | -1.061515 | -0.746262 | H | -2.724440 | 3.847833 | 0.225220 |
| C | 5.092581 | -0.049789 | -0.605986 | H | -3.635082 | 4.716200 | -1.043804 |
| C | 4.754025 | 1.144484 | 0.045798 | H | -7.357811 | 1.367052 | -2.506796 |
| C | 3.466938 | 1.315274 | 0.574386 | H | -1.677367 | 1.806305 | -0.158601 |
| C | 1.152342 | 0.467615 | 1.050935 | C | -0.409460 | -2.107103 | -1.363897 |
| C | 0.834280 | -0.523648 | 2.186036 | H | 0.294031 | -1.377329 | -1.777044 |
| C | 1.345572 | -0.065522 | 3.547047 | H | -1.386089 | -1.980851 | -1.845799 |
| C | -6.578067 | -1.695057 | 0.397958 | H | -0.044637 | -3.120496 | -1.548313 |
| C | -6.314057 | 1.068426 | -2.387385 | H | -3.046130 | -2.245307 | 0.148594 |
| C | -3.141094 | 3.777731 | -0.787518 | H | -2.359847 | -1.941366 | 2.583965 |
| C | 3.603426 | -3.223115 | -1.584312 | H | -1.095111 | -1.837109 | 3.820509 |
| C | 6.535868 | 0.160007 | -2.438538 | H | -0.838805 | -2.823788 | 2.355504 |
| C | 5.461786 | 3.295541 | 0.774589 | O | 6.365361 | -0.239619 | -1.079769 |
| H | 3.189539 | 2.235448 | 1.074723 | H | 2.097905 | -1.667799 | -0.290235 |
| O | -4.146369 | 2.778186 | -0.864688 | H | 1.041057 | 1.492328 | 1.423978 |
| O | -5.499815 | -1.767917 | -0.539991 | O | -0.505788 | -1.975281 | 0.052566 |
| O | -6.094671 | 0.932687 | -0.980721 | H | 3.281204 | -3.625850 | -0.615260 |
| H | 7.580168 | -0.040234 | -2.689657 | H | 4.122841 | -4.001360 | -2.145985 |
| H | 6.330299 | 1.230831 | -2.560751 | H | 2.718547 | -2.895012 | -2.145569 |
| H | 5.883322 | -0.419611 | -3.103020 | H | 5.200815 | 3.123145 | 1.827022 |
| H | -2.180215 | 0.559528 | 2.292009 | H | 4.645197 | 3.846083 | 0.288463 |
| H | 1.278751 | -1.491501 | 1.934550 | H | 6.377955 | 3.886176 | 0.721305 |
| O | 5.744577 | 2.080360 | 0.103785 | O | 0.119600 | 0.270481 | 0.053163 |

**Table S6.** Atomic coordinates (Å) of **1a**-5 obtained at the B3LYP-D3BJ/6-31G(d) level of theory in the gas phase.

| C | -2.220998 | -0.158779 | -0.012795 | H | 0.932011 | 1.152189 | 3.678540 |
| --- | --- | --- | --- | --- | --- | --- | --- |
| C | -2.530043 | 1.161821 | -0.330752 | H | 2.497577 | 0.376227 | 3.341866 |
| C | -3.843236 | 1.508717 | -0.665862 | H | 1.238244 | -0.508429 | 4.217367 |
| C | -4.848785 | 0.536633 | -0.693064 | O | 4.551003 | -2.090947 | -1.486392 |
| C | -4.532008 | -0.797880 | -0.367414 | H | -6.610880 | 1.938933 | 0.677170 |
| C | -3.222680 | -1.140568 | -0.027422 | H | -4.689283 | 4.203506 | -2.289381 |
| C | -0.822683 | -0.539747 | 0.440694 | H | -5.454061 | 2.600942 | -2.517386 |
| C | -0.642385 | -0.479427 | 1.993831 | H | -3.735410 | 2.850083 | -2.960702 |
| C | -1.206210 | -1.676023 | 2.747201 | H | -7.964474 | 1.386456 | -0.353651 |
| C | 2.525313 | 0.449848 | 0.274414 | O | -1.206807 | 0.735396 | 2.485048 |
| C | 2.856497 | -0.768063 | -0.326875 | H | -4.930880 | -3.132578 | 0.943549 |
| C | 4.125106 | -0.944566 | -0.882119 | H | -4.587315 | -3.467501 | -0.778884 |
| C | 5.069047 | 0.096521 | -0.838263 | H | -6.265416 | -3.548021 | -0.170727 |
| C | 4.737691 | 1.304431 | -0.208082 | H | -7.097055 | 0.211220 | 0.675588 |
| C | 3.458358 | 1.484054 | 0.336120 | H | -2.953784 | -2.159868 | 0.215390 |
| C | 1.165630 | 0.632074 | 0.898770 | C | -0.402871 | -2.052801 | -1.366424 |
| C | 0.881331 | -0.311919 | 2.081851 | H | -1.384426 | -1.954385 | -1.845918 |
| C | 1.416599 | 0.209956 | 3.410233 | H | -0.027971 | -3.068460 | -1.515697 |
| C | -4.530430 | 3.123561 | -2.254992 | H | 0.288524 | -1.331416 | -1.812286 |
| C | -6.992540 | 1.114010 | 0.063368 | H | -1.761249 | 1.923710 | -0.327657 |
| C | -5.310961 | -3.027113 | -0.081064 | H | -2.293471 | -1.727747 | 2.619644 |
| C | 3.624028 | -3.157736 | -1.587763 | H | -0.999136 | -1.562098 | 3.815744 |
| C | 7.282273 | -0.627457 | -0.580784 | H | -0.771208 | -2.612687 | 2.391536 |
| C | 5.435832 | 3.490412 | 0.419276 | O | 6.294116 | -0.055130 | -1.435539 |
| H | 3.180840 | 2.420175 | 0.805734 | H | 2.119690 | -1.559724 | -0.346245 |
| O | -5.577963 | -1.670899 | -0.400067 | H | 1.049333 | 1.670626 | 1.228307 |
| O | -4.131762 | 2.827066 | -0.912525 | O | -0.491006 | -1.868131 | 0.044762 |
| O | -6.130532 | 0.874311 | -1.052073 | H | 4.146089 | -3.958391 | -2.114493 |
| H | 8.201757 | -0.684343 | -1.168195 | H | 2.734459 | -2.863142 | -2.159599 |
| H | 6.990036 | -1.635775 | -0.262441 | H | 3.308732 | -3.516103 | -0.598687 |
| H | 7.451537 | 0.003647 | 0.300678 | H | 5.177342 | 3.378310 | 1.480802 |
| H | -2.109603 | 0.805926 | 2.132780 | H | 4.614178 | 4.003264 | -0.097459 |
| H | 1.328519 | -1.286534 | 1.863170 | H | 6.346464 | 4.085151 | 0.329506 |
| O | 5.728446 | 2.241052 | -0.181499 | O | 0.114386 | 0.380668 | -0.068023 |

**Table S7.** Key transitions, oscillator strengths, and rotatory strengths in the ECD spectrum of conformer **1a**-1 at the CAM-B3LYP/6-31+G(2d,p) level of theory in MeOH with IEFPCM solvent model.

| *Num^a^* | *Transition^b^* | *CI-coeff^b^* | *ΔE (eV)^d^* | *λ (nm)^e^* | *f^f^* | *R_vel_^g^* | *R_len_^h^* |
| --- | --- | --- | --- | --- | --- | --- | --- |
| 1 | 125->129 | 0.43635 | 5.0544 | 245.30 | 0.0207 | 1.4603 | 1.5762 |
|  | 126->129 | -0.31319 |  |  |  |  |  |
|  | 126->137 | -0.2112 |  |  |  |  |  |
| 2 | 127->129 | -0.2369 | 5.0799 | 244.07 | 0.0201 | 0.5805 | 0.3845 |
|  | 127->130 | -0.20938 |  |  |  |  |  |
|  | 127->131 | 0.40771 |  |  |  |  |  |
|  | 128->138 | -0.29832 |  |  |  |  |  |
| 3 | 125->129 | 0.19817 | 5.5075 | 225.12 | 0.3058 | -87.8749 | -86.4529 |
|  | 126->129 | 0.32145 |  |  |  |  |  |
|  | 128->129 | -0.29451 |  |  |  |  |  |
|  | 128->131 | 0.28617 |  |  |  |  |  |
| 4 | 125->129 | 0.24367 | 5.5415 | 223.74 | 0.0326 | 57.2753 | 57.9068 |
|  | 126->129 | 0.3617 |  |  |  |  |  |
|  | 128->130 | 0.21021 |  |  |  |  |  |
|  | 128->131 | -0.35729 |  |  |  |  |  |
| 5 | 128->130 | -0.31902 | 5.8467 | 212.06 | 0.0028 | 1.9348 | 1.9422 |
|  | 128->132 | 0.37724 |  |  |  |  |  |
|  | 128->133 | 0.21498 |  |  |  |  |  |
|  | 128->135 | -0.25859 |  |  |  |  |  |
| 6 | 126->130 | 0.34261 | 5.9012 | 210.10 | 0.0040 | -0.6409 | -0.5685 |
|  | 126->131 | 0.24827 |  |  |  |  |  |
|  | 126->132 | 0.21709 |  |  |  |  |  |
|  | 126->133 | 0.22945 |  |  |  |  |  |
|  | 126->134 | 0.18801 |  |  |  |  |  |
| 7 | 125->130 | 0.30818 | 5.9551 | 208.20 | 0.0008 | 6.5574 | 6.3895 |
|  | 125->131 | 0.24946 |  |  |  |  |  |
|  | 125->132 | 0.25639 |  |  |  |  |  |
|  | 127->130 | -0.21038 |  |  |  |  |  |
| 8 | 125->130 | -0.20449 | 5.9582 | 208.09 | 0.0079 | 29.926 | 29.3795 |
|  | 127->130 | -0.33579 |  |  |  |  |  |
|  | 127->132 | 0.34263 |  |  |  |  |  |
|  | 127->133 | 0.21454 |  |  |  |  |  |
| 9 | 126->137 | 0.1905 | 6.2481 | 198.43 | 0.1923 | 465.2191 | 470.2177 |
|  | 127->131 | 0.20675 |  |  |  |  |  |
|  | 128->138 | 0.3367 |  |  |  |  |  |
| 10 | 125->136 | 0.31539 | 6.2651 | 197.90 | 1.2949 | 375.3454 | 376.2642 |
|  | 125->137 | 0.33015 |  |  |  |  |  |
|  | 126->129 | -0.19292 |  |  |  |  |  |
|  | 128->138 | -0.21328 |  |  |  |  |  |
| 11 | 125->129 | -0.2007 | 6.3008 | 196.77 | 1.0292 | -1111.514 | -1121.6142 |
|  | 126->136 | -0.25039 |  |  |  |  |  |
|  | 126->137 | -0.26407 |  |  |  |  |  |
|  | 127->138 | 0.32209 |  |  |  |  |  |
| 12 | 125->136 | -0.23514 | 6.3822 | 194.27 | 0.2157 | 276.3897 | 276.7235 |
|  | 125->137 | -0.23738 |  |  |  |  |  |
|  | 126->136 | 0.20614 |  |  |  |  |  |
|  | 126->137 | 0.23017 |  |  |  |  |  |
|  | 127->138 | 0.24414 |  |  |  |  |  |
| 13 | 128->130 | -0.21714 | 6.5065 | 190.56 | 0.0129 | -0.536 | -1.3205 |
|  | 128->132 | 0.23889 |  |  |  |  |  |
|  | 128->133 | -0.2406 |  |  |  |  |  |
|  | 128->135 | 0.24426 |  |  |  |  |  |
| 14 | 127->130 | -0.19517 | 6.6173 | 187.36 | 0.0346 | 53.1649 | 54.1562 |
|  | 127->133 | -0.1965 |  |  |  |  |  |
|  | 127->134 | 0.20353 |  |  |  |  |  |
| 15 | 126->130 | 0.2509 | 6.6249 | 187.15 | 0.0052 | -33.6098 | -32.1908 |
|  | 126->135 | -0.21997 |  |  |  |  |  |
|  | 126->139 | 0.27838 |  |  |  |  |  |
| 16 | 125->130 | -0.21977 | 6.6770 | 185.69 | 0.0240 | -29.8693 | -29.8637 |
|  | 125->135 | 0.24843 |  |  |  |  |  |
|  | 125->139 | -0.23588 |  |  |  |  |  |
| 17 | 128->136 | 0.19584 | 6.6811 | 185.58 | 0.0072 | 12.9742 | 13.3832 |
|  | 128->137 | -0.18737 |  |  |  |  |  |
| 18 | 125->132 | -0.20855 | 6.7082 | 184.82 | 0.0204 | 4.8412 | 4.9914 |
|  | 125->133 | 0.25454 |  |  |  |  |  |
|  | 125->137 | -0.19401 |  |  |  |  |  |
|  | 126->133 | -0.19581 |  |  |  |  |  |
| 19 | 128->129 | -0.19502 | 6.7200 | 184.50 | 0.0025 | -2.7267 | -2.9095 |
|  | 128->134 | 0.37872 |  |  |  |  |  |
|  | 128->141 | 0.20451 |  |  |  |  |  |
| 20 | 122->129 | -0.2932 | 6.7262 | 184.33 | 0.0060 | 19.7744 | 20.4042 |
|  | 124->129 | -0.29331 |  |  |  |  |  |
|  | 128->129 | 0.32431 |  |  |  |  |  |
|  | 128->134 | 0.24588 |  |  |  |  |  |
| 21 | 127->133 | 0.22347 | 6.7479 | 183.74 | 0.0135 | -10.3818 | -9.9664 |
|  | 127->135 | 0.26558 |  |  |  |  |  |
|  | 127->140 | -0.24151 |  |  |  |  |  |
| 22 | 122->129 | -0.26244 | 6.7942 | 182.49 | 0.0070 | 7.0852 | 6.5648 |
|  | 127->134 | 0.31194 |  |  |  |  |  |
|  | 127->139 | 0.19213 |  |  |  |  |  |
|  | 127->141 | 0.21165 |  |  |  |  |  |
|  | 128->129 | -0.20296 |  |  |  |  |  |
| 23 | 126->132 | -0.21484 | 6.7957 | 182.45 | 0.0134 | 17.9137 | 17.7986 |
|  | 126->133 | 0.29254 |  |  |  |  |  |
| 24 | 122->129 | 0.32535 | 6.8115 | 182.02 | 0.0246 | -24.221 | -23.9694 |
|  | 127->134 | 0.1873 |  |  |  |  |  |
|  | 128->129 | 0.30365 |  |  |  |  |  |
| 25 | 126->134 | -0.22793 | 6.8439 | 181.16 | 0.0139 | 0.0068 | 0.1747 |
|  | 126->135 | 0.28298 |  |  |  |  |  |
|  | 126->136 | 0.1999 |  |  |  |  |  |
|  | 126->138 | 0.18801 |  |  |  |  |  |
|  | 126->140 | 0.26185 |  |  |  |  |  |
| 26 | 125->134 | -0.26761 | 6.8705 | 180.46 | 0.0211 | 4.8107 | 4.5924 |
|  | 125->135 | 0.29526 |  |  |  |  |  |
|  | 125->138 | 0.20613 |  |  |  |  |  |
|  | 125->140 | 0.25643 |  |  |  |  |  |
| 27 | 123->129 | -0.2819 | 6.8890 | 179.97 | 0.0068 | 2.9046 | 2.6367 |
|  | 123->130 | -0.27052 |  |  |  |  |  |
|  | 123->131 | 0.51298 |  |  |  |  |  |
| 28 | 124->129 | 0.20649 | 6.9399 | 178.66 | 0.0012 | -1.9397 | -1.9699 |
|  | 127->129 | 0.51633 |  |  |  |  |  |
|  | 127->131 | 0.23271 |  |  |  |  |  |
| 29 | 122->129 | -0.20029 | 7.0153 | 176.74 | 0.0015 | 1.8084 | 1.7698 |
|  | 124->129 | 0.38957 |  |  |  |  |  |
|  | 127->129 | -0.20824 |  |  |  |  |  |
| 30 | 124->129 | -0.23707 | 7.0318 | 176.32 | 0.0053 | 5.9675 | 5.8227 |
|  | 128->133 | -0.2232 |  |  |  |  |  |
|  | 128->140 | 0.25528 |  |  |  |  |  |
|  | 128->142 | -0.22436 |  |  |  |  |  |
| 31 | 127->140 | 0.19418 | 7.0683 | 175.41 | 0.0091 | -3.8755 | -3.4766 |
|  | 127->144 | 0.31404 |  |  |  |  |  |
|  | 128->144 | 0.29274 |  |  |  |  |  |
| 32 | 120->129 | -0.19428 | 7.0704 | 175.36 | 0.0043 | -4.2071 | -4.4229 |
|  | 124->131 | 0.19768 |  |  |  |  |  |
|  | 126->130 | -0.20465 |  |  |  |  |  |
|  | 126->131 | 0.24819 |  |  |  |  |  |
|  | 126->132 | -0.21467 |  |  |  |  |  |
| 33 | 126->131 | -0.3027 | 7.0928 | 174.80 | 0.0005 | 0.8447 | 1.0389 |
|  | 126->141 | 0.30435 |  |  |  |  |  |
| 34 | 125->129 | -0.18973 | 7.1181 | 174.18 | 0.0053 | 4.0591 | 3.9025 |
|  | 125->130 | -0.21104 |  |  |  |  |  |
|  | 125->131 | 0.4049 |  |  |  |  |  |
| 35 | 125->133 | 0.31361 | 7.1659 | 173.02 | 0.0064 | 5.2583 | 5.6702 |
|  | 125->141 | -0.20585 |  |  |  |  |  |
|  | 125->142 | 0.22399 |  |  |  |  |  |
| 36 | 127->142 | 0.27019 | 7.1778 | 172.73 | 0.0012 | -7.923 | -8.7203 |
|  | 127->144 | 0.19543 |  |  |  |  |  |
|  | 127->146 | 0.21147 |  |  |  |  |  |
|  | 128->144 | -0.21641 |  |  |  |  |  |

*^a^*Number of the excited states; *^b^*Only transitions with contribution over 10.0% were listed; *^c^*Configuration-interaction coefficient; *^d^*Excitation energy; *^e^*Wavelength; *^f^*Oscillator strength; *^g^*Rotatory strength in velocity form (10^-40^ cgs); *^h^*Rotatory strength in length form (10^-40^ cgs).

**Computational data of 2**

**Table S8.** Experimental ^13^C-NMR chemical shifts of **2**, and calculated ^13^C-NMR chemical shifts of **2a** and **2b**.

| **Num.** | **^13^C exp** | **2a** | **2b** |
| --- | --- | --- | --- |
| 1 | 132.3 | 129.4 | 132.0 |
| 2,6 | 106.6 | 104.7 | 105.5 |
| 3 | 152.7 | 151.6 | 149.7 |
| 4 | 142.3 | 141.0 | 140.3 |
| 5 | 152.7 | 151.4 | 150.0 |
| 7 | 197.9 | 196.8 | 194.3 |
| 8 | 139.4 | 134.1 | 143.8 |
| 9 | 17.5 | 21.2 | 20.4 |
| 1′ | 130.4 | 126.3 | 129.9 |
| 2′ | 110.2 | 110.1 | 105.8 |
| 3′ | 148.9 | 146.2 | 146.2 |
| 4′ | 153.3 | 151.5 | 150.1 |
| 5′ | 109.5 | 107.4 | 107.4 |
| 6′ | 124.9 | 122.8 | 122.3 |
| 7′ | 197.7 | 195.5 | 194.2 |
| 8′ | 138.0 | 136.6 | 144.8 |
| 9′ | 17.3 | 22.1 | 20.5 |
| 3,5-OMe | 56.1 | 54.7 | 55.8 |
| 4-OMe | 60.9 | 58.8 | 58.5 |
| 3′-OMe | 55.8 | 54.3 | 55.1 |
| 4′-OMe | 56.0 | 54.7 | 54.5 |
| **R^2^** |  | 0.9988 | 0.9972 |
| **MAE** |  | 2.2 | 2.6 |
| **CMAE** |  | 1.5 | 1.9 |

**Table S9.** Experimental ^1^H-NMR chemical shifts of **2**, and calculated ^1^H-NMR chemical shifts of **2a** and **2b**.

| **Num.** | **^1^H exp** | **2a** | **2b** |
| --- | --- | --- | --- |
| 2,6 | 6.71 | 7.63 | 6.20 |
| 9 | 2.17 | 1.67 | 2.21 |
| 2′ | 6.93 | 7.82 | 6.24 |
| 5′ | 6.74 | 7.15 | 6.68 |
| 6′ | 7.18 | 8.11 | 6.98 |
| 9′ | 2.17 | 1.69 | 2.26 |
| 3,5-OMe | 3.78 | 3.89 | 3.70 |
| 4-OMe | 3.87 | 3.68 | 3.73 |
| 3′-OMe | 3.77 | 3.87 | 3.70 |
| 4′-OMe | 3.90 | 3.90 | 3.83 |
| **R^2^** |  | 0.9959 | 0.9914 |
| **MAE** |  | 0.45 | 0.20 |
| **CMAE** |  | 0.09 | 0.12 |


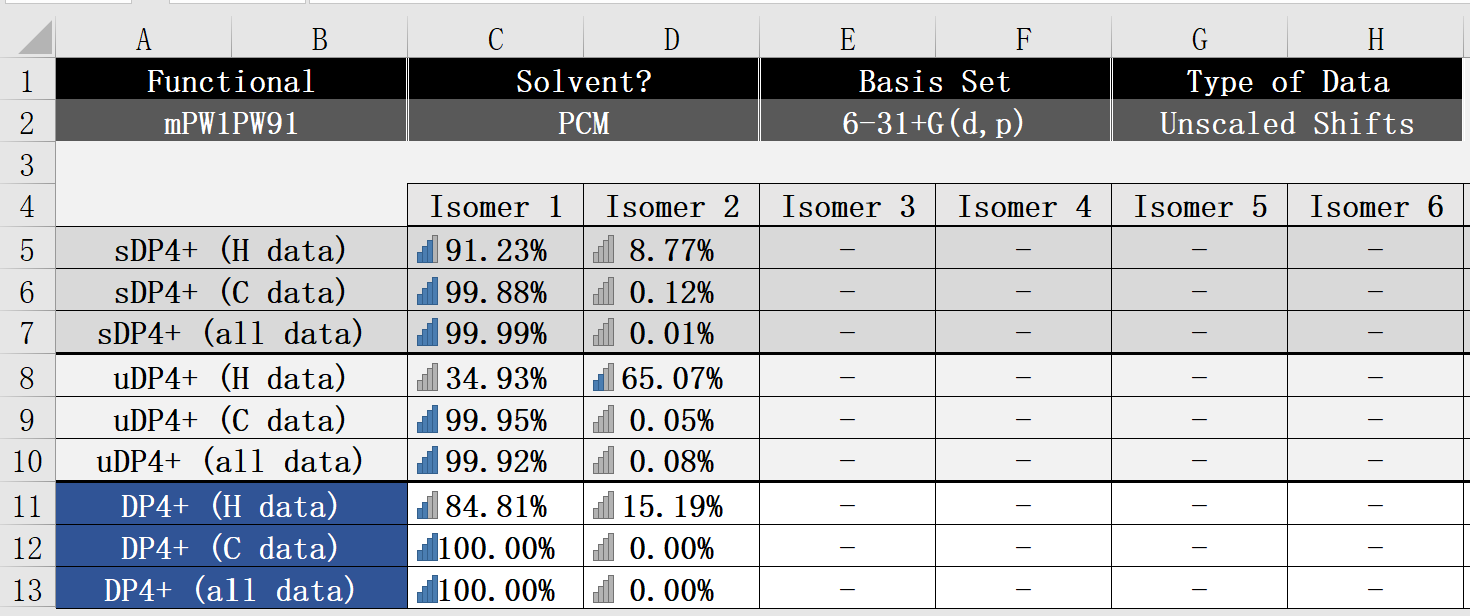


**Figure 48.** DP4+ probability analysis of **2a** and **2b**

**Table S10.** Conformational analysis of the B3LYP-D3BJ/6-31G(d) optimized conformers of **2a** in the gas phase (T=298.15 K)

| Conformer | E (Hartree)*^a^* | C (Hartree)*^b^* | G (kcal/mol)*^c^* | *Δ*G (kcal/mol)*^d^* | Population*^e^* |
| --- | --- | --- | --- | --- | --- |
| **2a**-1 | -1418.450468 | 0.379567 | -889839.49012 | 0.0 | 38.66% |
| **2a**-2 | -1418.450196 | 0.379788 | -889839.181064 | 0.309056 | 22.94% |
| **2a**-3 | -1418.450259 | 0.379932 | -889839.130305 | 0.359815 | 21.05% |
| **2a**-4 | -1418.450335 | 0.38019 | -889839.015906 | 0.474214 | 17.35% |

*^a^*Electronic energy obtained at M06-2X-D3/6-311+G(2d,p) level of theory; *^b^*Thermal correction to Gibbs free energy obtained at B3LYP-D3BJ/6-31G(d) level of theory; *^c^*Gibbs free energy (E + C); *^d^*The relative Gibbs free energy; *^e^*The Boltzmann distribution of each conformer.

**Table S11.** Atomic coordinates (Å) of **2a**-1 obtained at the B3LYP-D3BJ/6-31G(d) level of theory in the gas phase.

| C | -2.534590 | -0.799127 | 0.191288 | O | 6.580180 | 1.409309 | 0.166866 |
| --- | --- | --- | --- | --- | --- | --- | --- |
| C | -3.695845 | -1.561436 | 0.013148 | O | -1.204548 | -2.739249 | 0.527096 |
| C | -4.918253 | -0.917688 | -0.169590 | H | -3.603135 | -2.639469 | 0.026258 |
| C | -4.981187 | 0.490099 | -0.179441 | H | -1.678792 | 1.165738 | 0.354424 |
| C | -3.813466 | 1.245723 | 0.025114 | H | 0.851984 | -1.868273 | 2.122666 |
| C | -2.588024 | 0.600327 | 0.205716 | H | 1.582098 | -0.254479 | 1.965851 |
| C | -1.247624 | -1.520990 | 0.395317 | H | -0.041441 | -0.445478 | 2.656679 |
| C | 0.021295 | -0.714774 | 0.521306 | H | -0.386500 | 1.104743 | -2.110298 |
| C | 0.650176 | -0.816751 | 1.889236 | H | 0.646412 | -0.221563 | -2.643064 |
| C | 3.069221 | 0.291777 | -0.239430 | H | -0.969419 | -0.570073 | -2.000675 |
| C | 4.168681 | 1.160118 | -0.082113 | H | 2.441594 | -1.759245 | -0.430189 |
| C | 5.456165 | 0.661041 | 0.010106 | H | 4.729327 | -2.671128 | -0.278080 |
| C | 5.667387 | -0.745020 | -0.060967 | H | 3.964528 | 2.222396 | -0.038773 |
| C | 4.577179 | -1.600282 | -0.221572 | H | -6.484471 | 1.888950 | 1.493729 |
| C | 3.282490 | -1.085118 | -0.310226 | H | -7.890855 | 1.804401 | 0.394083 |
| C | 1.718678 | 0.885918 | -0.349454 | H | -7.268096 | 0.310584 | 1.152709 |
| C | 0.514995 | -0.009743 | -0.509987 | H | -5.741898 | -3.384426 | 0.580214 |
| C | -0.094632 | 0.072044 | -1.888240 | H | -7.136969 | -3.271476 | -0.531991 |
| C | -6.101088 | -2.969445 | -0.370265 | H | -5.476339 | -3.351490 | -1.187389 |
| C | -6.992136 | 1.284328 | 0.732145 | H | -2.344309 | 3.219560 | 1.171209 |
| C | -2.818048 | 3.396533 | 0.196983 | H | -2.081714 | 3.211066 | -0.595003 |
| C | 6.417700 | 2.816865 | 0.242170 | H | -3.160754 | 4.430761 | 0.141707 |
| C | 7.233008 | -2.533683 | -0.024780 | H | 6.913633 | -2.961808 | -0.983396 |
| O | 1.539913 | 2.101378 | -0.367837 | H | 8.315429 | -2.625911 | 0.072899 |
| O | -3.979705 | 2.595783 | 0.029454 | H | 6.743513 | -3.074716 | 0.794916 |
| O | -6.165074 | 1.126428 | -0.422513 | H | 5.967024 | 3.217998 | -0.674774 |
| O | 6.957657 | -1.142203 | 0.037386 | H | 5.796974 | 3.103003 | 1.101129 |
| O | -6.111159 | -1.550184 | -0.347758 | H | 7.421595 | 3.225927 | 0.364840 |

**Table S12.** Atomic coordinates (Å) of **2a**-2 obtained at the B3LYP-D3BJ/6-31G(d) level of theory in the gas phase.

| C | 2.307494 | 0.689446 | -0.334356 | O | -5.084896 | 2.127319 | 0.261228 |
| --- | --- | --- | --- | --- | --- | --- | --- |
| C | 3.351098 | 1.607118 | -0.163293 | O | 0.683302 | 2.419269 | -0.466608 |
| C | 4.664175 | 1.146873 | -0.074032 | H | 3.098645 | 2.657255 | -0.097933 |
| C | 4.936035 | -0.232104 | -0.177625 | H | 1.740208 | -1.372043 | -0.557370 |
| C | 3.879435 | -1.145216 | -0.343227 | H | -0.188482 | 0.083256 | -2.703117 |
| C | 2.564526 | -0.684373 | -0.428575 | H | -1.746504 | -0.376129 | -1.989656 |
| C | 0.919784 | 1.216088 | -0.431533 | H | -1.267438 | 1.333108 | -2.086420 |
| C | -0.227074 | 0.244372 | -0.559287 | H | 0.916618 | 0.084949 | 1.894784 |
| C | -0.906136 | 0.314372 | -1.905221 | H | -0.637424 | -0.389996 | 2.606717 |
| C | -3.125403 | -1.019465 | 0.225427 | H | 0.451519 | -1.628516 | 1.984983 |
| C | -3.409778 | 0.359110 | 0.301642 | H | -3.940385 | -2.986582 | 0.011730 |
| C | -4.714063 | 0.820343 | 0.206291 | H | -6.289218 | -2.193287 | -0.162474 |
| C | -5.772681 | -0.111252 | 0.036240 | H | -2.592405 | 1.056207 | 0.429247 |
| C | -5.486500 | -1.477348 | -0.033100 | H | 7.698100 | -1.473352 | 0.987269 |
| C | -4.171729 | -1.928481 | 0.062779 | H | 6.587661 | -0.400769 | 1.887513 |
| C | -1.737273 | -1.527888 | 0.333538 | H | 6.058075 | -2.046896 | 1.406616 |
| C | -0.599016 | -0.544384 | 0.462698 | H | 5.081036 | 3.731329 | -0.713932 |
| C | 0.081929 | -0.612199 | 1.807750 | H | 4.894144 | 3.592605 | 1.058546 |
| C | 5.535153 | 3.338276 | 0.204450 | H | 6.521080 | 3.784449 | 0.343257 |
| C | 6.655146 | -1.176002 | 1.114235 | H | 2.486746 | -3.387356 | 0.243699 |
| C | 3.208365 | -3.414639 | -0.582625 | H | 2.675090 | -3.257161 | -1.528437 |
| C | -4.053309 | 3.090536 | 0.415510 | H | 3.704513 | -4.385921 | -0.602491 |
| C | -8.112586 | -0.453915 | -0.214788 | H | -8.997878 | 0.182076 | -0.254330 |
| O | -1.481350 | -2.727801 | 0.376405 | H | -8.029887 | -1.024582 | -1.148552 |
| O | 4.242563 | -2.456033 | -0.406609 | H | -8.199280 | -1.150411 | 0.628860 |
| O | 6.230545 | -0.665106 | -0.151467 | H | -4.551445 | 4.060979 | 0.421094 |
| O | -7.012411 | 0.427391 | -0.047311 | H | -3.512481 | 2.951092 | 1.360544 |
| O | 5.753530 | 1.938677 | 0.116513 | H | -3.336423 | 3.050359 | -0.414741 |

**Table S13.** Atomic coordinates (Å) of **2a**-3 obtained at the B3LYP-D3BJ/6-31G(d) level of theory in the gas phase.

| C | 2.304156 | 0.662814 | -0.176222 | O | -5.086961 | 2.141857 | 0.110052 |
| --- | --- | --- | --- | --- | --- | --- | --- |
| C | 2.567049 | -0.712831 | -0.204915 | O | 0.687237 | 2.381499 | -0.455767 |
| C | 3.877761 | -1.165513 | -0.043659 | H | 1.750358 | -1.407634 | -0.343846 |
| C | 4.922410 | -0.243741 | 0.147743 | H | 3.083944 | 2.641791 | -0.005504 |
| C | 4.648773 | 1.138661 | 0.152064 | H | -1.682231 | -0.472509 | -1.955723 |
| C | 3.338837 | 1.590188 | -0.003187 | H | -1.207034 | 1.235023 | -2.093168 |
| C | 0.921634 | 1.181354 | -0.356816 | H | -0.101495 | -0.029432 | -2.627699 |
| C | -0.218829 | 0.203720 | -0.492807 | H | 0.357428 | -1.573568 | 2.143551 |
| C | -0.848319 | 0.224999 | -1.864418 | H | 0.821030 | 0.137288 | 2.013412 |
| C | -3.150830 | -1.016927 | 0.237714 | H | -0.760319 | -0.319198 | 2.675519 |
| C | -3.427100 | 0.365206 | 0.259701 | H | -3.973638 | -2.984791 | 0.064527 |
| C | -4.724530 | 0.831370 | 0.110210 | H | -6.310380 | -2.182701 | -0.204873 |
| C | -5.784634 | -0.098510 | -0.059807 | H | -2.608856 | 1.061072 | 0.388493 |
| C | -5.506597 | -1.467929 | -0.075663 | H | 7.008296 | -0.151054 | -1.482463 |
| C | -4.198538 | -1.924105 | 0.073838 | H | 7.907308 | -1.339649 | -0.495830 |
| C | -1.769920 | -1.529721 | 0.403298 | H | 6.421387 | -1.842196 | -1.352794 |
| C | -0.632006 | -0.546916 | 0.541947 | H | 3.718918 | -4.415341 | -0.155240 |
| C | -0.007462 | -0.565681 | 1.915513 | H | 2.480292 | -3.374382 | 0.603631 |
| C | 3.220596 | -3.446129 | -0.202839 | H | 2.708530 | -3.344759 | -1.168268 |
| C | 6.914772 | -1.021077 | -0.820901 | H | 4.851322 | 3.624738 | 1.176291 |
| C | 5.508285 | 3.343845 | 0.343345 | H | 6.489566 | 3.799625 | 0.483832 |
| C | -4.053403 | 3.103042 | 0.264018 | H | 5.068552 | 3.701987 | -0.596450 |
| C | -8.119287 | -0.434041 | -0.364467 | H | -8.016449 | -1.036015 | -1.276316 |
| O | -1.522266 | -2.729094 | 0.486420 | H | -8.233395 | -1.101428 | 0.499190 |
| O | 5.733991 | 1.942988 | 0.311645 | H | -8.998481 | 0.206076 | -0.448564 |
| O | 6.198354 | -0.677115 | 0.367293 | H | -4.544040 | 4.076335 | 0.220297 |
| O | -7.017426 | 0.445116 | -0.197099 | H | -3.543927 | 2.992884 | 1.230102 |
| O | 4.247958 | -2.475770 | -0.054209 | H | -3.310707 | 3.029529 | -0.540916 |

**Table S14.** Atomic coordinates (Å) of **2a**-4 obtained at the B3LYP-D3BJ/6-31G(d) level of theory in the gas phase.

| C | -2.539631 | -0.835358 | 0.195020 | O | 6.554927 | 1.440715 | 0.165681 |
| --- | --- | --- | --- | --- | --- | --- | --- |
| C | -2.592897 | 0.564323 | 0.207728 | O | -1.207339 | -2.774673 | 0.529461 |
| C | -3.816779 | 1.208485 | 0.020907 | H | -1.683080 | 1.130599 | 0.349034 |
| C | -4.988472 | 0.454702 | -0.171994 | H | -3.609723 | -2.674230 | 0.034895 |
| C | -4.926130 | -0.953620 | -0.159916 | H | -0.049365 | -0.469264 | 2.655761 |
| C | -3.701506 | -1.596201 | 0.017815 | H | 0.847132 | -1.892235 | 2.127592 |
| C | -1.252292 | -1.556543 | 0.397664 | H | 1.574245 | -0.277647 | 1.964920 |
| C | 0.014983 | -0.747078 | 0.521506 | H | -0.975004 | -0.617709 | -2.000875 |
| C | 0.643399 | -0.841984 | 1.890035 | H | -0.404464 | 1.061007 | -2.115948 |
| C | 3.055957 | 0.286331 | -0.240363 | H | 0.638357 | -0.259429 | -2.643915 |
| C | 4.146216 | 1.166311 | -0.083682 | H | 2.449401 | -1.771084 | -0.428683 |
| C | 5.438748 | 0.680695 | 0.009567 | H | 4.746660 | -2.659298 | -0.274529 |
| C | 5.664536 | -0.723225 | -0.059649 | H | 3.931087 | 2.226464 | -0.041680 |
| C | 4.583344 | -1.590025 | -0.219471 | H | -7.018008 | 0.854520 | 1.485038 |
| C | 3.283456 | -1.088398 | -0.309264 | H | -6.188386 | 2.416118 | 1.179472 |
| C | 1.699353 | 0.865686 | -0.351822 | H | -7.752828 | 2.061143 | 0.390773 |
| C | 0.504793 | -0.042001 | -0.511561 | H | -2.076280 | 3.156925 | -0.629914 |
| C | -0.105028 | 0.031258 | -1.890256 | H | -3.151431 | 4.395637 | 0.080490 |
| C | -2.812704 | 3.361490 | 0.156912 | H | -2.341864 | 3.201234 | 1.135188 |
| C | -6.811169 | 1.630988 | 0.738010 | H | -5.736866 | -3.419718 | 0.599844 |
| C | -6.103862 | -3.006629 | -0.348674 | H | -7.140346 | -3.311243 | -0.501766 |
| C | 6.377894 | 2.846629 | 0.238735 | H | -5.484577 | -3.389922 | -1.169660 |
| C | 7.248728 | -2.495413 | -0.021100 | H | 6.764870 | -3.040666 | 0.799144 |
| O | 1.507199 | 2.079237 | -0.371379 | H | 6.933899 | -2.927831 | -0.979282 |
| O | -6.117499 | -1.587839 | -0.328653 | H | 8.332046 | -2.576179 | 0.076737 |
| O | -6.184451 | 1.066052 | -0.416090 | H | 5.923308 | 3.241586 | -0.678960 |
| O | 6.958801 | -1.106799 | 0.039598 | H | 5.754003 | 3.127692 | 1.097081 |
| O | -3.977372 | 2.561400 | 0.002802 | H | 7.377469 | 3.266249 | 0.360999 |

**Table S15.** Conformational analysis of the B3LYP-D3BJ/6-31G(d) optimized conformers of **2b** in the gas phase (T=298.15 K)

| Conformer | E (Hartree)*^a^* | C (Hartree)*^b^* | G (kcal/mol)*^c^* | *Δ*G (kcal/mol)*^d^* | Population*^e^* |
| --- | --- | --- | --- | --- | --- |
| **2b**-1 | -1418.460969 | 0.385482 | -889842.368061 | 0.0 | 59.02% |
| **2b**-2 | -1418.459654 | 0.385329 | -889841.638837 | 0.729224 | 17.22% |
| **2b**-3 | -1418.458314 | 0.38434 | -889841.418792 | 0.949269 | 11.88% |
| **2b**-4 | -1418.458286 | 0.385281 | -889840.810694 | 1.557367 | 4.25% |
| **2b**-5 | -1418.459504 | 0.386543 | -889840.782808 | 1.585253 | 4.06% |
| **2b**-6 | -1418.459549 | 0.38671 | -889840.706686 | 1.661375 | 3.57% |

*^a^*Electronic energy obtained at M06-2X-D3/6-311+G(2d,p) level of theory; *^b^*Thermal correction to Gibbs free energy obtained at B3LYP-D3BJ/6-31G(d) level of theory; *^c^*Gibbs free energy (E + C); *^d^*The relative Gibbs free energy; *^e^*The Boltzmann distribution of each conformer.

**Table S16.** Atomic coordinates (Å) of **2b**-1 obtained at the B3LYP-D3BJ/6-31G(d) level of theory in the gas phase.

| C | -0.920157 | 0.374172 | 1.303680 | O | -0.832766 | 3.593180 | -0.497694 |
| --- | --- | --- | --- | --- | --- | --- | --- |
| C | -1.388279 | 1.507487 | 0.632024 | O | -1.558673 | -1.536586 | 2.549832 |
| C | -0.485399 | 2.481135 | 0.200315 | H | -2.441831 | 1.603661 | 0.414270 |
| C | 0.885506 | 2.320748 | 0.459525 | H | 0.758231 | -0.672662 | 2.113675 |
| C | 1.342736 | 1.193115 | 1.171355 | H | -3.919554 | -1.931011 | 2.588017 |
| C | 0.438159 | 0.230436 | 1.612269 | H | -4.707499 | -0.385435 | 2.269347 |
| C | -1.832047 | -0.751195 | 1.647572 | H | -5.094649 | -1.792632 | 1.261378 |
| C | -3.130452 | -0.926446 | 0.888444 | H | -4.533745 | -0.242492 | -2.039258 |
| C | -4.288856 | -1.281179 | 1.790179 | H | -4.692310 | -1.947389 | -1.611598 |
| C | -0.790906 | -1.262383 | -1.176194 | H | -5.410783 | -0.709186 | -0.565814 |
| C | 0.394184 | -0.640448 | -1.615668 | H | -1.632875 | -2.983106 | -0.188088 |
| C | 1.628322 | -1.201572 | -1.332363 | H | 0.544481 | -4.041275 | 0.244159 |
| C | 1.685173 | -2.446699 | -0.646329 | H | 0.311345 | 0.319818 | -2.107201 |
| C | 0.508429 | -3.089868 | -0.271517 | H | 3.054025 | 4.737899 | 0.238437 |
| C | -0.727091 | -2.493417 | -0.524209 | H | 1.672169 | 4.691391 | 1.370009 |
| C | -2.068665 | -0.538375 | -1.371254 | H | 3.022334 | 3.523424 | 1.551035 |
| C | -3.237940 | -0.832575 | -0.458631 | H | -2.191103 | 4.533468 | -1.649937 |
| C | -4.551013 | -0.939654 | -1.197346 | H | -2.388280 | 2.752835 | -1.605772 |
| C | -2.159066 | 3.646801 | -1.014696 | H | -2.897836 | 3.755192 | -0.209142 |
| C | 2.414003 | 4.085293 | 0.835874 | H | 3.060079 | -0.923092 | 1.216338 |
| C | 3.246911 | -0.067341 | 1.871790 | H | 4.321663 | 0.111284 | 1.931209 |
| C | 2.849776 | 0.633214 | -2.240014 | H | 2.856041 | -0.267842 | 2.876954 |
| C | 3.057227 | -4.128604 | 0.329587 | H | 2.584945 | -4.069245 | 1.318522 |
| O | 2.833644 | -0.652308 | -1.624104 | H | 2.612792 | -4.958506 | -0.233872 |
| O | 2.693826 | 1.126054 | 1.328384 | H | 4.128321 | -4.299764 | 0.445181 |
| O | 2.936666 | -2.902070 | -0.375892 | H | 2.447149 | 1.402738 | -1.574780 |
| O | 1.768564 | 3.208889 | -0.091865 | H | 3.901436 | 0.843475 | -2.440558 |
| O | -2.201597 | 0.298718 | -2.262738 | H | 2.294831 | 0.625565 | -3.186250 |

**Table S17.** Atomic coordinates (Å) of **2b**-2 obtained at the B3LYP-D3BJ/6-31G(d) level of theory in the gas phase.

| C | 0.926433 | -0.525073 | 1.344564 | O | 0.316071 | -3.771942 | -0.241033 |
| --- | --- | --- | --- | --- | --- | --- | --- |
| C | 1.161159 | -1.755657 | 0.729093 | O | 1.933615 | 1.267996 | 2.521151 |
| C | 0.085553 | -2.559245 | 0.356518 | H | 2.163596 | -2.095486 | 0.502323 |
| C | -1.224781 | -2.126268 | 0.580288 | H | -0.520702 | 0.852256 | 2.106934 |
| C | -1.459582 | -0.898804 | 1.241249 | H | 4.854392 | -0.467476 | 2.144454 |
| C | -0.383107 | -0.114848 | 1.642070 | H | 5.407357 | 0.881972 | 1.136667 |
| C | 2.036725 | 0.422956 | 1.637908 | H | 4.328362 | 1.176557 | 2.517357 |
| C | 3.320660 | 0.333347 | 0.843104 | H | 4.979735 | 0.968603 | -1.710454 |
| C | 4.556358 | 0.490823 | 1.697226 | H | 5.467866 | -0.369437 | -0.654504 |
| C | 1.025066 | 1.048433 | -1.181294 | H | 4.479254 | -0.680105 | -2.097595 |
| C | -0.263891 | 0.660808 | -1.596688 | H | 2.197197 | 2.597237 | -0.247671 |
| C | -1.361148 | 1.459483 | -1.322084 | H | 0.274601 | 4.071808 | 0.168022 |
| C | -1.166845 | 2.709674 | -0.671756 | H | -0.376266 | -0.306219 | -2.068197 |
| C | 0.117282 | 3.118595 | -0.321036 | H | -3.801499 | -4.112243 | 0.478398 |
| C | 1.209503 | 2.284805 | -0.563799 | H | -2.385941 | -4.310576 | 1.552386 |
| C | 2.136923 | 0.087720 | -1.377678 | H | -3.486737 | -2.907438 | 1.762062 |
| C | 3.365869 | 0.179264 | -0.501904 | H | 0.470938 | -4.729693 | -2.024206 |
| C | 4.654365 | 0.014946 | -1.272267 | H | -0.838343 | -3.514552 | -1.962964 |
| C | 0.194606 | -3.734190 | -1.669892 | H | 0.872317 | -2.984654 | -2.094634 |
| C | -3.025619 | -3.584760 | 1.036873 | H | -2.754196 | 1.507870 | 1.234345 |
| C | -3.085858 | 0.716741 | 1.913234 | H | -4.173903 | 0.742266 | 1.989737 |
| C | -2.929269 | -0.137495 | -2.141221 | H | -2.647763 | 0.863018 | 2.908170 |
| C | -2.172305 | 4.653953 | 0.261437 | H | -1.577482 | 5.363820 | -0.326712 |
| O | -2.654335 | 1.147274 | -1.591125 | H | -3.187502 | 5.036442 | 0.375198 |
| O | -2.772185 | -0.570736 | 1.393234 | H | -1.712830 | 4.529199 | 1.250316 |
| O | -2.301924 | 3.408823 | -0.408979 | H | -2.665937 | -0.937284 | -1.442364 |
| O | -2.265497 | -2.855102 | 0.067465 | H | -4.006027 | -0.152289 | -2.316702 |
| O | 2.086288 | -0.777877 | -2.249430 | H | -2.405338 | -0.282334 | -3.094324 |

**Table S18.** Atomic coordinates (Å) of **2b**-3 obtained at the B3LYP-D3BJ/6-31G(d) level of theory in the gas phase.

| C | 0.885307 | 0.466116 | -1.374551 | O | -2.700518 | 0.362667 | -2.091348 |
| --- | --- | --- | --- | --- | --- | --- | --- |
| C | -0.352857 | 0.052217 | -1.871764 | O | 2.037425 | -1.172744 | -2.629350 |
| C | -1.511795 | 0.744734 | -1.529833 | H | -0.414527 | -0.821237 | -2.508601 |
| C | -1.436404 | 1.847623 | -0.665128 | H | 1.940493 | 1.929601 | -0.198824 |
| C | -0.183427 | 2.274625 | -0.178752 | H | 4.982393 | 0.718417 | -1.907062 |
| C | 0.976674 | 1.607110 | -0.564952 | H | 5.394744 | -0.888122 | -1.297575 |
| C | 2.069512 | -0.381061 | -1.692322 | H | 4.356745 | -0.724203 | -2.728127 |
| C | 3.349224 | -0.277503 | -0.892606 | H | 4.617385 | 0.283355 | 2.167972 |
| C | 4.594175 | -0.297881 | -1.752076 | H | 5.273986 | -1.075137 | 1.234266 |
| C | 1.099806 | -1.105773 | 1.171480 | H | 5.446911 | 0.574475 | 0.626537 |
| C | -0.140668 | -0.689770 | 1.691716 | H | 2.131291 | -2.627603 | 0.045090 |
| C | -1.294687 | -1.400996 | 1.421954 | H | 0.099802 | -3.944766 | -0.394370 |
| C | -1.212239 | -2.589977 | 0.645031 | H | -0.166396 | 0.231957 | 2.256507 |
| C | 0.027229 | -3.034292 | 0.187229 | H | -2.027987 | 4.451355 | -0.562156 |
| C | 1.180775 | -2.290027 | 0.440727 | H | -2.898809 | 3.666472 | -1.921911 |
| C | 2.253023 | -0.206405 | 1.390700 | H | -3.775456 | 4.097769 | -0.424191 |
| C | 3.435672 | -0.233103 | 0.459225 | H | -3.336004 | -1.090162 | -0.724437 |
| C | 4.770875 | -0.101476 | 1.158308 | H | -3.958802 | 0.558553 | -0.429525 |
| C | -3.689448 | -0.169496 | -1.198303 | H | -4.559294 | -0.386773 | -1.822331 |
| C | -2.823748 | 3.750329 | -0.831394 | H | 0.748687 | 4.511740 | 1.996087 |
| C | 1.008942 | 3.713615 | 1.299141 | H | 1.450556 | 2.870623 | 1.841871 |
| C | -2.683204 | 0.191941 | 2.512909 | H | 1.729851 | 4.095824 | 0.564613 |
| C | -2.389929 | -4.339374 | -0.456150 | H | -1.976348 | -4.096789 | -1.442863 |
| O | -2.543774 | -1.043298 | 1.817911 | H | -1.815714 | -5.162626 | -0.013148 |
| O | -0.215183 | 3.339089 | 0.671344 | H | -3.433749 | -4.638336 | -0.559084 |
| O | -2.397343 | -3.199644 | 0.392194 | H | -2.365493 | 1.039353 | 1.895260 |
| O | -2.589244 | 2.463356 | -0.248544 | H | -3.747759 | 0.283023 | 2.733419 |
| O | 2.261475 | 0.597051 | 2.324908 | H | -2.116058 | 0.184384 | 3.452140 |

**Table S19.** Atomic coordinates (Å) of **2b**-4 obtained at the B3LYP-D3BJ/6-31G(d) level of theory in the gas phase.

| C | -1.050638 | 0.452361 | 1.284436 | O | -0.808788 | 3.483683 | -0.804790 |
| --- | --- | --- | --- | --- | --- | --- | --- |
| C | -1.461182 | 1.516961 | 0.476787 | O | -1.800997 | -1.313561 | 2.673187 |
| C | -0.521059 | 2.441941 | 0.018242 | H | -2.496933 | 1.596349 | 0.180437 |
| C | 0.827075 | 2.301294 | 0.384623 | H | 0.557671 | -0.520626 | 2.302072 |
| C | 1.225882 | 1.240392 | 1.223860 | H | -5.157518 | -1.844340 | 1.153679 |
| C | 0.282192 | 0.331118 | 1.695613 | H | -4.156033 | -1.627948 | 2.605532 |
| C | -1.996342 | -0.629069 | 1.674335 | H | -5.002844 | -0.245261 | 1.900136 |
| C | -3.229370 | -0.887448 | 0.836276 | H | -5.387754 | -0.735661 | -0.800827 |
| C | -4.462679 | -1.171915 | 1.661262 | H | -4.404699 | -0.593217 | -2.273410 |
| C | -0.741783 | -1.381718 | -1.034524 | H | -4.674323 | -2.178830 | -1.538116 |
| C | 0.455449 | -0.785270 | -1.465127 | H | -1.626306 | -3.012171 | 0.070973 |
| C | 1.681741 | -1.298259 | -1.059815 | H | 0.582612 | -3.984357 | 0.716761 |
| C | 1.710414 | -2.473973 | -0.268324 | H | 0.388207 | 0.126554 | -2.043584 |
| C | 0.520906 | -3.089578 | 0.106527 | H | 1.579668 | 4.744653 | 1.126316 |
| C | -0.706757 | -2.544466 | -0.259148 | H | 2.893112 | 3.583650 | 1.509816 |
| C | -2.014675 | -0.697142 | -1.376668 | H | 3.039186 | 4.672298 | 0.098255 |
| C | -3.237908 | -0.918123 | -0.518117 | H | -2.289433 | 2.543768 | -1.935857 |
| C | -4.504485 | -1.115585 | -1.318974 | H | -2.887844 | 3.676972 | -0.681773 |
| C | -2.094536 | 3.489946 | -1.417988 | H | -2.073556 | 4.311339 | -2.136080 |
| C | 2.347080 | 4.084282 | 0.704621 | H | 2.901297 | -0.880770 | 1.626691 |
| C | 3.060750 | 0.047669 | 2.182981 | H | 4.132131 | 0.223781 | 2.290804 |
| C | 2.921881 | 0.485816 | -2.064922 | H | 2.603461 | -0.026453 | 3.177062 |
| C | 3.858883 | -3.413526 | -0.711088 | H | 3.433089 | -4.182438 | -1.367865 |
| O | 2.890375 | -0.733188 | -1.323677 | H | 4.233899 | -2.581353 | -1.310146 |
| O | 2.562891 | 1.175264 | 1.467633 | H | 4.669120 | -3.847930 | -0.122216 |
| O | 2.880046 | -2.975345 | 0.238678 | H | 2.465022 | 1.306590 | -1.505840 |
| O | 1.753592 | 3.133417 | -0.183977 | H | 3.980254 | 0.701179 | -2.219355 |
| O | -2.096608 | 0.041167 | -2.356415 | H | 2.429391 | 0.368463 | -3.037788 |

**Table S20.** Atomic coordinates (Å) of **2b**-5 obtained at the B3LYP-D3BJ/6-31G(d) level of theory in the gas phase.

| C | -0.626098 | 0.556260 | 1.283003 | O | 0.363991 | 3.706492 | -0.359838 |
| --- | --- | --- | --- | --- | --- | --- | --- |
| C | -0.759745 | 1.821352 | 0.702419 | O | -1.705201 | -1.218759 | 2.411433 |
| C | 0.381558 | 2.500664 | 0.272588 | H | -1.744117 | 2.236136 | 0.537964 |
| C | 1.653490 | 1.923269 | 0.444975 | H | 0.687140 | -0.976233 | 1.972279 |
| C | 1.775774 | 0.676059 | 1.087390 | H | -5.069689 | -0.949766 | 1.320624 |
| C | 0.636916 | 0.002647 | 1.517278 | H | -4.013384 | -0.741121 | 2.728817 |
| C | -1.795161 | -0.306420 | 1.590344 | H | -4.807878 | 0.663520 | 1.986257 |
| C | -3.112335 | -0.113739 | 0.884921 | H | -5.305399 | 0.843359 | -0.405383 |
| C | -4.319306 | -0.298333 | 1.779964 | H | -4.561074 | 0.770643 | -2.014833 |
| C | -0.962850 | -0.595279 | -1.457710 | H | -5.111234 | -0.714501 | -1.212467 |
| C | -0.946388 | -1.849705 | -0.818904 | H | 0.182736 | 0.856367 | -2.536133 |
| C | 0.239020 | -2.559034 | -0.689615 | H | 2.302522 | -0.405595 | -2.362052 |
| C | 1.432468 | -2.030514 | -1.246436 | H | -1.854823 | -2.230506 | -0.372992 |
| C | 1.394820 | -0.824481 | -1.946607 | H | 3.490270 | 3.384850 | 1.684970 |
| C | 0.205548 | -0.110836 | -2.048861 | H | 4.130593 | 4.002756 | 0.135189 |
| C | -2.140172 | 0.301497 | -1.439228 | H | 2.480100 | 4.451873 | 0.655361 |
| C | -3.261829 | 0.106481 | -0.444664 | H | -1.527229 | 4.476968 | 0.087795 |
| C | -4.638670 | 0.264353 | -1.051466 | H | -0.687377 | 5.107965 | -1.354102 |
| C | -0.903234 | 4.208742 | -0.775009 | H | -1.432028 | 3.479277 | -1.398703 |
| C | 3.233524 | 3.657747 | 0.653651 | H | 2.713944 | -1.871538 | 1.359616 |
| C | 3.202996 | -1.047053 | 1.890271 | H | 4.279211 | -1.224543 | 1.925471 |
| C | -0.758285 | -4.205366 | 0.703099 | H | 2.810315 | -0.982222 | 2.912508 |
| C | 3.797896 | -2.179201 | -1.423640 | H | 3.850165 | -2.089439 | -2.515709 |
| O | 0.377130 | -3.730696 | -0.012195 | H | 3.948146 | -1.194535 | -0.965251 |
| O | 3.042831 | 0.188760 | 1.199414 | H | 4.568302 | -2.871545 | -1.081138 |
| O | 2.560006 | -2.748240 | -1.010332 | H | -0.420631 | -5.098267 | 1.231555 |
| O | 2.772834 | 2.513698 | -0.069866 | H | -1.117559 | -3.458868 | 1.421161 |
| O | -2.236247 | 1.241023 | -2.229460 | H | -1.573385 | -4.477940 | 0.019364 |

**Table S21.** Atomic coordinates (Å) of **2b**-6 obtained at the B3LYP-D3BJ/6-31G(d) level of theory in the gas phase.

| C | -0.808896 | 0.364481 | 1.360918 | O | -0.408436 | 3.802901 | 0.087549 |
| --- | --- | --- | --- | --- | --- | --- | --- |
| C | -1.169992 | 1.625691 | 0.876613 | O | -1.571163 | -1.684819 | 2.260119 |
| C | -0.174326 | 2.564923 | 0.603973 | H | -2.208411 | 1.847624 | 0.676291 |
| C | 1.177294 | 2.246841 | 0.831365 | H | 0.756660 | -0.936414 | 2.000397 |
| C | 1.523431 | 0.982092 | 1.346415 | H | -4.877936 | -1.937735 | 0.884035 |
| C | 0.526998 | 0.056174 | 1.639518 | H | -3.930377 | -1.775179 | 2.373951 |
| C | -1.792101 | -0.737723 | 1.506094 | H | -4.919298 | -0.422135 | 1.791333 |
| C | -3.070381 | -0.742853 | 0.706813 | H | -4.689661 | -1.450598 | -1.746307 |
| C | -4.269296 | -1.250765 | 1.479129 | H | -5.326210 | -0.199672 | -0.670727 |
| C | -0.708516 | -0.609977 | -1.467608 | H | -4.468220 | 0.258374 | -2.157517 |
| C | -0.509023 | -1.891005 | -0.919778 | H | 0.222777 | 1.094167 | -2.365268 |
| C | 0.772085 | -2.399290 | -0.759605 | H | 2.519628 | 0.184663 | -2.155447 |
| C | 1.884514 | -1.637453 | -1.201774 | H | -1.360262 | -2.455907 | -0.566451 |
| C | 1.675635 | -0.402175 | -1.815512 | H | 1.592871 | 3.663119 | -1.366045 |
| C | 0.388882 | 0.110969 | -1.941766 | H | 3.245429 | 4.104140 | -0.847586 |
| C | -2.027159 | 0.065301 | -1.477864 | H | 2.861883 | 2.402763 | -1.235136 |
| C | -3.164158 | -0.420682 | -0.606723 | H | -1.676788 | 5.060107 | -0.844527 |
| C | -4.492073 | -0.452987 | -1.329743 | H | -2.054403 | 3.327522 | -1.105658 |
| C | -1.728004 | 4.076527 | -0.374970 | H | -2.441042 | 4.113397 | 0.458882 |
| C | 2.472771 | 3.333685 | -0.804262 | H | 2.945533 | -1.351387 | 1.469589 |
| C | 3.235926 | -0.491773 | 2.084227 | H | 4.323425 | -0.454441 | 2.167533 |
| C | -0.002427 | -4.300077 | 0.437581 | H | 2.796881 | -0.590868 | 3.084417 |
| C | 4.247553 | -1.384574 | -1.246895 | H | 4.213992 | -0.429766 | -0.707986 |
| O | 1.072269 | -3.581629 | -0.159974 | H | 5.106045 | -1.968808 | -0.912875 |
| O | 2.856792 | 0.740217 | 1.478366 | H | 4.333564 | -1.196010 | -2.324303 |
| O | 3.104232 | -2.176854 | -0.949374 | H | -0.715898 | -4.649686 | -0.320509 |
| O | 2.164656 | 3.157250 | 0.579275 | H | 0.455662 | -5.162842 | 0.923512 |
| O | -2.232215 | 1.040503 | -2.200935 | H | -0.527339 | -3.687048 | 1.179337 |

**Table S22.** Experimental and calculated ^13^C-NMR chemical shifts of **3**.

| **Num.** | **^13^C exp** | **3-*δ*_calcd._** |
| --- | --- | --- |
| 1 | 135.6 | 138.0 |
| 2 | 22.2 | 23.9 |
| 3 | 33.8 | 35.4 |
| 4 | 71.8 | 71.9 |
| 5 | 76.4 | 74.8 |
| 6 | 140.2 | 140.2 |
| 7 | 41.5 | 43.5 |
| 8 | 21.3 | 22.3 |
| 9 | 32.8 | 33.8 |
| 10 | 73.1 | 73.7 |
| 11 | 74.1 | 75.8 |
| 12 | 28.4 | 28.3 |
| 13 | 29.4 | 29.1 |
| 14 | 22.2 | 22.9 |
| 15 | 24.5 | 24.7 |
| **R^2^** |  | 0.9993 |
| **MAE** |  | 1.0 |
| **CMAE** |  | 0.8 |

**Table S23.** Experimental and calculated ^1^H-NMR chemical shifts of **3**.

| **Num.** | **^13^C exp** | **3-*δ*_calcd._** |
| --- | --- | --- |
| H-2a | 2.53 | 2.09 |
| H-2b | 2.07 | 2.05 |
| H-3a | 2.07 | 1.69 |
| H-3b | 1.97 | 1.50 |
| H-5 | 4.44 | 3.83 |
| H-7 | 2.76 | 2.39 |
| H-8a | 1.52 | 1.03 |
| H-8b | 2.07 | 2.09 |
| H-9a | 1.33 | 1.18 |
| H-9b | 1.76 | 1.55 |
| H-12 | 1.33 | 1.11 |
| H-13 | 1.17 | 0.74 |
| H-14 | 1.36 | 1.04 |
| H-15 | 1.64 | 1.10 |
| **R^2^** |  | 0.9506 |
| **MAE** |  | 0.33 |
| **CMAE** |  | 0.15 |

**Table S24.** Conformational analysis of the B3LYP-D3BJ/6-31G(d) optimized conformers of **3** in the gas phase (T=298.15 K)

| Conformer | E (Hartree) | C (Hartree) | G (kcal/mol) | ΔG (kcal/mol) | Population |
| --- | --- | --- | --- | --- | --- |
| **3**-1 | -811.628842 | 0.324146 | -509093.696571 | 0.0 | 36.03% |
| **3**-2 | -811.627121 | 0.323045 | -509093.307432 | 0.389138 | 18.67% |
| **3**-3 | -811.627391 | 0.323442 | -509093.227894 | 0.468677 | 16.33% |
| **3**-4 | -811.627218 | 0.323568 | -509093.040167 | 0.656403 | 11.89% |
| **3**-5 | -811.626558 | 0.32291 | -509093.039148 | 0.657423 | 11.87% |
| **3**-6 | -811.626235 | 0.323363 | -509092.552154 | 1.144416 | 5.21% |

*^a^*Electronic energy obtained at M06-2X-D3/6-311+G(2d,p) level of theory; *^b^*Thermal correction to Gibbs free energy obtained at B3LYP-D3BJ/6-31G(d) level of theory; *^c^*Gibbs free energy (E + C); *^d^*The relative Gibbs free energy; *^e^*The Boltzmann distribution of each conformer.

**Table S25.** Atomic coordinates (Å) of **3a**-1 obtained at the B3LYP-D3BJ/6-31G(d) level of theory in the gas phase.

| C | -0.006352 | 0.922719 | -0.141050 | H | -2.178270 | 0.788503 | -2.066317 |
| --- | --- | --- | --- | --- | --- | --- | --- |
| C | -1.062709 | 1.899403 | -0.585378 | H | -4.850793 | 0.103458 | -0.383417 |
| C | -2.336016 | 1.174954 | -1.051271 | H | -4.246268 | -1.511143 | 0.060155 |
| C | -2.708875 | -0.008963 | -0.149788 | H | -4.036892 | -0.911340 | -1.597566 |
| C | -1.575560 | -1.062501 | -0.170606 | H | -2.020654 | 0.826449 | 1.483711 |
| C | -0.236202 | -0.391304 | 0.026432 | H | -1.589491 | -1.602685 | -1.126871 |
| C | 0.978542 | -1.168434 | 0.469455 | H | -2.189213 | -1.540971 | 1.577592 |
| C | 1.400139 | -0.572834 | 1.835974 | H | 0.764662 | -2.235858 | 0.556550 |
| C | 1.743203 | 0.923093 | 1.624753 | H | 0.568845 | -0.698075 | 2.536545 |
| C | 1.438048 | 1.308113 | 0.155893 | H | 2.250849 | -1.117316 | 2.255289 |
| C | 2.103137 | -0.918237 | -0.576799 | H | 1.177489 | 1.569259 | 2.305910 |
| C | 3.438971 | -1.546108 | -0.166494 | H | 2.807299 | 1.113283 | 1.802021 |
| C | 1.698645 | -1.428443 | -1.964191 | H | 1.185942 | 3.442263 | 0.442914 |
| C | 1.797863 | 2.752525 | -0.147682 | H | 2.850552 | 2.927282 | 0.092402 |
| C | -4.046262 | -0.621079 | -0.541786 | H | 1.651772 | 2.972122 | -1.209500 |
| O | -2.863896 | 0.417900 | 1.222798 | H | 0.760541 | -0.970606 | -2.288105 |
| O | -1.808929 | -2.046268 | 0.834180 | H | 1.578245 | -2.517995 | -1.963296 |
| O | 2.310778 | 0.510054 | -0.682968 | H | 2.472286 | -1.165542 | -2.692722 |
| H | -1.302853 | 2.599697 | 0.230023 | H | 3.816789 | -1.117755 | 0.764981 |
| H | -0.681865 | 2.530864 | -1.397146 | H | 4.186327 | -1.358948 | -0.944142 |
| H | -3.178085 | 1.874416 | -1.096970 | H | 3.339726 | -2.630260 | -0.038907 |

**Table S26.** Atomic coordinates (Å) of **3a**-2 obtained at the B3LYP-D3BJ/6-31G(d) level of theory in the gas phase.

| C | -0.005611 | 0.940161 | 0.017042 | H | -3.288480 | 1.970178 | -0.241021 |
| --- | --- | --- | --- | --- | --- | --- | --- |
| C | -1.106849 | 1.915198 | -0.288807 | H | -1.717658 | -0.010619 | -2.235883 |
| C | -2.467779 | 1.358479 | 0.149174 | H | -3.448516 | 0.400152 | -2.243315 |
| C | -2.676584 | -0.099087 | -0.285284 | H | -2.938673 | -1.288597 | -2.067762 |
| C | -1.577839 | -0.989409 | 0.348968 | H | -3.980950 | -0.365710 | 1.118393 |
| C | -0.225071 | -0.350586 | 0.310825 | H | -1.546973 | -1.945000 | -0.198515 |
| C | 1.028789 | -1.124108 | 0.636219 | H | -2.585053 | -1.940998 | 1.714219 |
| C | 1.662992 | -0.424810 | 1.864191 | H | 0.817552 | -2.176761 | 0.847750 |
| C | 2.009367 | 1.030151 | 1.460764 | H | 0.935994 | -0.447730 | 2.680226 |
| C | 1.474073 | 1.298746 | 0.031408 | H | 2.552147 | -0.962612 | 2.205209 |
| C | 1.987921 | -1.004763 | -0.584366 | H | 1.577996 | 1.753605 | 2.161319 |
| C | 3.369861 | -1.609176 | -0.312021 | H | 3.092955 | 1.192425 | 1.444897 |
| C | 1.383754 | -1.647978 | -1.837062 | H | 2.894442 | 2.829876 | -0.468380 |
| C | 1.808904 | 2.695880 | -0.462243 | H | 1.440383 | 2.844532 | -1.481826 |
| C | -2.691427 | -0.255769 | -1.802166 | H | 1.367958 | 3.459248 | 0.186350 |
| O | -3.956777 | -0.561517 | 0.167086 | H | 1.278885 | -2.732236 | -1.711687 |
| O | -1.887041 | -1.266491 | 1.729731 | H | 2.034707 | -1.461230 | -2.697203 |
| O | 2.176064 | 0.400405 | -0.867716 | H | 0.401577 | -1.223217 | -2.058217 |
| H | -0.921298 | 2.873542 | 0.212452 | H | 3.291014 | -2.673192 | -0.059422 |
| H | -1.116153 | 2.145651 | -1.364593 | H | 3.880629 | -1.093725 | 0.504542 |
| H | -2.521456 | 1.389856 | 1.246183 | H | 3.993986 | -1.514194 | -1.206371 |

**Table S27.** Atomic coordinates (Å) of **3a**-3 obtained at the B3LYP-D3BJ/6-31G(d) level of theory in the gas phase.

| C | 0.002801 | 0.923462 | -0.127177 | H | -2.092674 | 0.778251 | -2.072000 |
| --- | --- | --- | --- | --- | --- | --- | --- |
| C | -1.070622 | 1.896319 | -0.525615 | H | -3.926691 | -0.993276 | -1.690152 |
| C | -2.304793 | 1.161770 | -1.065090 | H | -4.845711 | 0.027835 | -0.576414 |
| C | -2.709914 | -0.019658 | -0.175199 | H | -4.224716 | -1.554927 | -0.031666 |
| C | -1.564602 | -1.056374 | -0.108436 | H | -3.726658 | 0.839646 | 1.277633 |
| C | -0.224788 | -0.385866 | 0.052180 | H | -1.576261 | -1.653004 | -1.030598 |
| C | 0.995230 | -1.164522 | 0.478190 | H | -2.089532 | -1.430621 | 1.691941 |
| C | 1.433441 | -0.563104 | 1.837494 | H | 0.783928 | -2.232409 | 0.571980 |
| C | 1.770295 | 0.932656 | 1.615899 | H | 0.609799 | -0.683678 | 2.547060 |
| C | 1.450064 | 1.309601 | 0.148133 | H | 2.290522 | -1.104500 | 2.248587 |
| C | 2.107577 | -0.919697 | -0.581787 | H | 1.204219 | 1.577951 | 2.296748 |
| C | 3.447857 | -1.547998 | -0.185873 | H | 2.835371 | 1.127569 | 1.783934 |
| C | 1.686286 | -1.434639 | -1.962622 | H | 1.200626 | 3.443877 | 0.426391 |
| C | 1.806993 | 2.751934 | -0.167380 | H | 2.862414 | 2.928609 | 0.059772 |
| C | -4.005736 | -0.676416 | -0.644657 | H | 1.648147 | 2.965483 | -1.228811 |
| O | -2.854806 | 0.425046 | 1.193059 | H | 2.450524 | -1.173432 | -2.701815 |
| O | -1.811389 | -1.988378 | 0.942432 | H | 0.743989 | -0.977041 | -2.274371 |
| O | 2.316505 | 0.506924 | -0.695601 | H | 1.566452 | -2.524489 | -1.957354 |
| H | -1.356932 | 2.505912 | 0.343148 | H | 3.349545 | -2.631977 | -0.054452 |
| H | -0.700191 | 2.598035 | -1.282560 | H | 3.836432 | -1.117778 | 0.740301 |
| H | -3.152225 | 1.853731 | -1.162292 | H | 4.186620 | -1.363303 | -0.972487 |

**Table S28.** Atomic coordinates (Å) of **3a**-4 obtained at the B3LYP-D3BJ/6-31G(d) level of theory in the gas phase.

| C | -0.016944 | 0.929886 | 0.001145 | H | -3.301637 | 1.927270 | -0.321147 |
| --- | --- | --- | --- | --- | --- | --- | --- |
| C | -1.126022 | 1.895537 | -0.311970 | H | -3.358461 | 0.254322 | -2.307421 |
| C | -2.488882 | 1.327613 | 0.112535 | H | -2.761037 | -1.396327 | -2.050772 |
| C | -2.651926 | -0.141720 | -0.293108 | H | -1.614928 | -0.049972 | -2.215136 |
| C | -1.580822 | -0.985184 | 0.434896 | H | -4.598208 | -0.022203 | -0.022018 |
| C | -0.224690 | -0.352874 | 0.333062 | H | -1.560082 | -1.986080 | -0.030692 |
| C | 1.036552 | -1.106847 | 0.673892 | H | -2.855220 | -1.287040 | 1.837426 |
| C | 1.670328 | -0.361855 | 1.876096 | H | 0.834562 | -2.153787 | 0.919594 |
| C | 2.010893 | 1.078843 | 1.419918 | H | 0.943898 | -0.357566 | 2.692719 |
| C | 1.460207 | 1.298324 | -0.011931 | H | 2.561965 | -0.884198 | 2.234340 |
| C | 1.990172 | -1.019514 | -0.553581 | H | 1.585718 | 1.825050 | 2.100086 |
| C | 3.380556 | -1.596254 | -0.263775 | H | 3.094058 | 1.242012 | 1.386911 |
| C | 1.390784 | -1.716531 | -1.779174 | H | 1.335498 | 3.461913 | 0.071950 |
| C | 1.780835 | 2.681363 | -0.552948 | H | 2.865087 | 2.825090 | -0.568272 |
| C | -2.586571 | -0.343806 | -1.807492 | H | 1.407532 | 2.793160 | -1.575643 |
| O | -3.903032 | -0.660900 | 0.199136 | H | 2.032669 | -1.547248 | -2.649780 |
| O | -1.896369 | -1.114102 | 1.820971 | H | 0.398938 | -1.319007 | -2.006902 |
| O | 2.158595 | 0.376512 | -0.889625 | H | 1.307146 | -2.797446 | -1.615380 |
| H | -0.954567 | 2.852371 | 0.197393 | H | 3.999775 | -1.530297 | -1.164233 |
| H | -1.126101 | 2.133157 | -1.386201 | H | 3.315296 | -2.649995 | 0.032042 |
| H | -2.580676 | 1.375005 | 1.202944 | H | 3.888418 | -1.042162 | 0.528941 |

**Table S29.** Atomic coordinates (Å) of **3a**-5 obtained at the B3LYP-D3BJ/6-31G(d) level of theory in the gas phase.

| C | 0.004260 | 0.926050 | -0.090523 | H | -2.028351 | 0.771905 | -2.057974 |
| --- | --- | --- | --- | --- | --- | --- | --- |
| C | -1.073393 | 1.897199 | -0.480502 | H | -4.817532 | -0.001938 | -0.737891 |
| C | -2.284581 | 1.158222 | -1.062878 | H | -4.227590 | -1.579283 | -0.147684 |
| C | -2.722034 | -0.020810 | -0.192027 | H | -3.813333 | -1.033860 | -1.779700 |
| C | -1.566282 | -1.046773 | -0.045921 | H | -3.539509 | -0.138101 | 1.574255 |
| C | -0.223316 | -0.384784 | 0.082813 | H | -1.573967 | -1.729538 | -0.905319 |
| C | 1.005041 | -1.170231 | 0.477249 | H | -1.711696 | -1.317653 | 1.855431 |
| C | 1.484264 | -0.581157 | 1.828128 | H | 0.794490 | -2.239079 | 0.566746 |
| C | 1.814408 | 0.916598 | 1.610327 | H | 0.686742 | -0.707421 | 2.567287 |
| C | 1.459274 | 1.305313 | 0.154088 | H | 2.352855 | -1.126710 | 2.207675 |
| C | 2.092462 | -0.921323 | -0.608402 | H | 1.265389 | 1.556564 | 2.309701 |
| C | 3.440918 | -1.554994 | -0.249806 | H | 2.883142 | 1.109722 | 1.754454 |
| C | 1.636066 | -1.426332 | -1.981559 | H | 1.220716 | 3.438109 | 0.450483 |
| C | 1.813634 | 2.748532 | -0.159283 | H | 2.873867 | 2.921042 | 0.047595 |
| C | -3.972738 | -0.698489 | -0.749073 | H | 1.633924 | 2.968731 | -1.215862 |
| O | -3.006510 | 0.526464 | 1.110141 | H | 2.382381 | -1.161171 | -2.737343 |
| O | -1.816656 | -1.899163 | 1.083198 | H | 0.687199 | -0.965419 | -2.266941 |
| O | 2.301183 | 0.505361 | -0.717111 | H | 1.515083 | -2.516083 | -1.980608 |
| H | -1.385825 | 2.481586 | 0.395916 | H | 3.343583 | -2.639452 | -0.121543 |
| H | -0.696330 | 2.620277 | -1.213344 | H | 3.855046 | -1.130452 | 0.667852 |
| H | -3.131681 | 1.842779 | -1.184440 | H | 4.159364 | -1.367979 | -1.054358 |

**Table S30.** Atomic coordinates (Å) of **3a**-6 obtained at the B3LYP-D3BJ/6-31G(d) level of theory in the gas phase.

| C | -0.004196 | 0.924151 | -0.107039 | H | -2.095192 | 0.795306 | -2.057649 |
| --- | --- | --- | --- | --- | --- | --- | --- |
| C | -1.077451 | 1.899820 | -0.495948 | H | -4.836383 | 0.058499 | -0.615658 |
| C | -2.309236 | 1.173211 | -1.049334 | H | -4.268421 | -1.526865 | -0.048489 |
| C | -2.725571 | -0.004275 | -0.163653 | H | -3.910802 | -1.000718 | -1.706276 |
| C | -1.577249 | -1.039753 | -0.106949 | H | -2.804440 | -0.241578 | 1.743045 |
| C | -0.233006 | -0.386655 | 0.059872 | H | -1.592876 | -1.647495 | -1.026486 |
| C | 0.998723 | -1.166402 | 0.462758 | H | -1.096539 | -2.390672 | 1.249202 |
| C | 1.447921 | -0.587796 | 1.828521 | H | 0.823726 | -2.248214 | 0.528997 |
| C | 1.779070 | 0.911402 | 1.621255 | H | 0.630010 | -0.713645 | 2.544739 |
| C | 1.444621 | 1.308199 | 0.162025 | H | 2.309584 | -1.133067 | 2.224225 |
| C | 2.101098 | -0.907386 | -0.607518 | H | 1.217129 | 1.544748 | 2.315673 |
| C | 3.444861 | -1.540414 | -0.230590 | H | 2.845094 | 1.104989 | 1.782860 |
| C | 1.663745 | -1.406465 | -1.988917 | H | 1.192611 | 3.436837 | 0.469626 |
| C | 1.798270 | 2.754632 | -0.135487 | H | 2.854355 | 2.929791 | 0.089635 |
| C | -4.014027 | -0.661497 | -0.668488 | H | 1.633874 | 2.981745 | -1.192959 |
| O | -2.930521 | 0.519413 | 1.148500 | H | 2.417419 | -1.129980 | -2.732955 |
| O | -1.894079 | -1.895977 | 1.010427 | H | 0.715065 | -0.950285 | -2.281937 |
| O | 2.304910 | 0.517370 | -0.701491 | H | 1.552027 | -2.497414 | -1.999023 |
| H | -1.368834 | 2.490763 | 0.382471 | H | 3.842196 | -1.121090 | 0.696834 |
| H | -0.699734 | 2.612043 | -1.239327 | H | 4.175355 | -1.344616 | -1.021856 |
| H | -3.153626 | 1.866368 | -1.134237 | H | 3.350477 | -2.626515 | -0.112994 |

**Table S31.** Experimental ^13^C-NMR chemical shifts of **4**, and calculated ^13^C-NMR chemical shifts of **1a**–**4d**.

| No. | δ*_exptl._* | **4a**-*δ*_calcd._ | **4b**-*δ*calcd. | **4c**-*δ*_calcd._ | **4d**-*δ*_calcd._ |
| --- | --- | --- | --- | --- | --- |
| 1 | 159.6 | 162.4 | 162.7 | 159.6 | 164.2 |
| 2 | 23.8 | 26.5 | 28.3 | 23.8 | 26.8 |
| 3 | 36.3 | 39.4 | 39.2 | 36.3 | 35.4 |
| 4 | 73.4 | 73.7 | 73.6 | 73.4 | 73.6 |
| 5 | 202.7 | 202.6 | 202.4 | 202.7 | 202.0 |
| 6 | 137.2 | 135.4 | 134.8 | 137.2 | 134.1 |
| 7 | 38.7 | 39.9 | 38.4 | 38.7 | 39.6 |
| 8 | 20.2 | 21.3 | 22.1 | 20.2 | 22.5 |
| 9 | 31.3 | 32.7 | 31.4 | 31.3 | 32.1 |
| 10 | 74.1 | 76.2 | 76.6 | 74.1 | 76.6 |
| 11 | 30.7 | 31.7 | 33.1 | 30.7 | 34.1 |
| 12 | 19.1 | 21.5 | 21.5 | 19.1 | 19.1 |
| 13 | 21.4 | 18.5 | 19.3 | 21.4 | 21.3 |
| 14 | 67.1 | 68.5 | 66.2 | 67.1 | 67.7 |
| 15 | 24.1 | 25.4 | 25.4 | 24.1 | 25.9 |
| **R^2^** |  | 0.9992 | 0.9988 | 0.9977 | 0.9989 |
| **MAE** |  | 1.7 | 1.8 | 2.1 | 1.7 |
| **CMAE** |  | 1.1 | 1.6 | 2.1 | 1.5 |

**Table S32.** Experimental ^1^H-NMR chemical shifts of **4**, and calculated ^1^H-NMR chemical shifts of **1a**–**4d**.

| No. | δ*_exptl._* | **4a**-*δ*_calcd._ | **4b**-*δ*calcd. | **4c**-*δ*_calcd._ | **4d**-*δ*_calcd._ |
| --- | --- | --- | --- | --- | --- |
| H-2a | 2.58 | 2.53 | 2.69 | 2.73 | 2.56 |
| H-2b | 2.58 | 3.04 | 2.63 | 2.64 | 3.08 |
| H-3a | 1.99 | 1.93 | 1.94 | 1.85 | 1.87 |
| H-3b | 1.92 | 1.76 | 1.82 | 1.77 | 1.73 |
| H-7 | 2.58 | 2.69 | 2.6 | 2.45 | 2.38 |
| H-8a | 1.62 | 1.41 | 1.56 | 1.35 | 1.58 |
| H-8b | 1.62 | 1.58 | 1.67 | 1.52 | 1.55 |
| H-9a | 2.22 | 1.99 | 1.92 | 2.09 | 1.83 |
| H-9b | 1.41 | 1.35 | 1.37 | 1.09 | 1.44 |
| H-11 | 2.1 | 1.91 | 1.8 | 2.48 | 2.16 |
| H-12 | 0.79 | 0.68 | 0.8 | 0.45 | 0.66 |
| H-13 | 0.85 | 0.58 | 0.67 | 0.74 | 0.78 |
| H-14a | 3.49 | 3.55 | 3.39 | 3.54 | 3.47 |
| H-14b | 3.66 | 3.58 | 3.57 | 3.64 | 3.6 |
| H-15 | 1.24 | 1.13 | 1.15 | 1.08 | 1.02 |
| **R^2^** |  | 0.9704 | 0.981 | 0.9722 | 0.9563 |
| **MAE** |  | 0.15 | 0.1 | 0.17 | 0.14 |
| **CMAE** |  | 0.1 | 0.09 | 0.1 | 0.11 |


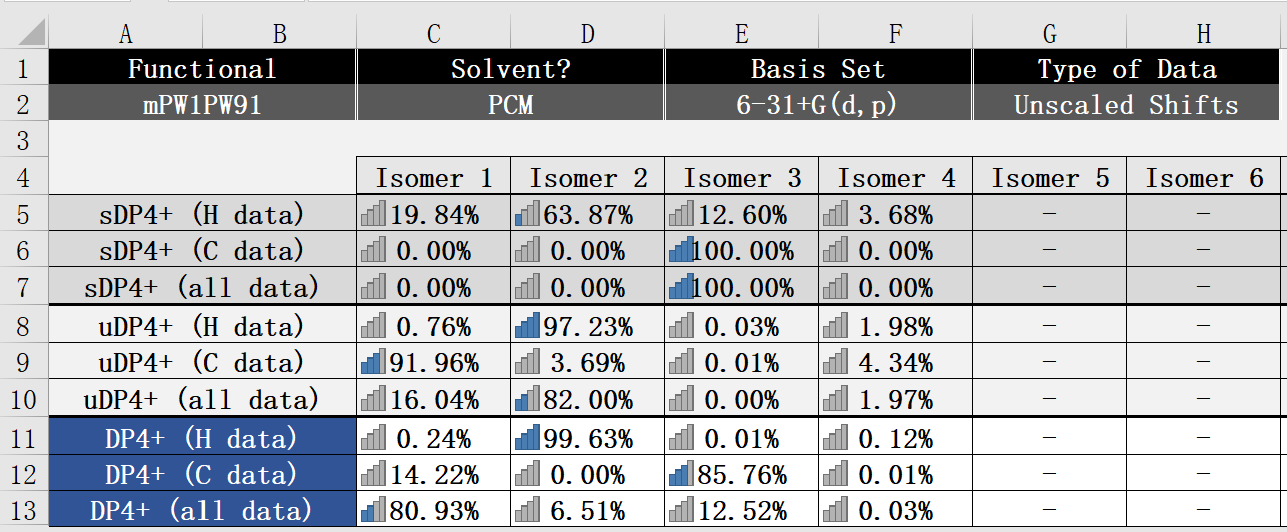


**Figure S49.** DP4+ probability analysis of **4a**–**4d**.


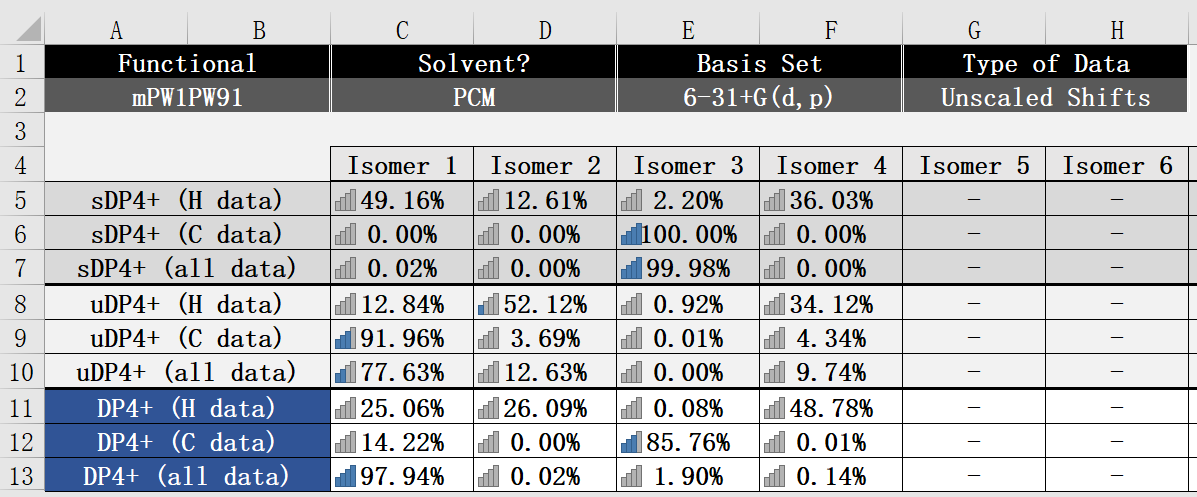


**Figure S50.** DP4+ probability analysis of **4a**–**4d** (proton chemical shifts assigned to methylene were not counted).

**Table S33.** Conformational analysis of the B3LYP-D3BJ/6-31G(d) optimized conformers of **4a** in the gas phase (T=298.15 K)

| Conformer | E (Hartree)*^a^* | C (Hartree)*^b^* | G (kcal/mol)*^c^* | *Δ*G (kcal/mol)*^d^* | Population*^e^* |
| --- | --- | --- | --- | --- | --- |
| **4a**-1 | -886.85851 | 0.324626 | -556300.011904 | 0.0 | 36.27% |
| **4a**-2 | -886.857854 | 0.324563 | -556299.640193 | 0.371711 | 19.36% |
| **4a**-3 | -886.857156 | 0.32431 | -556299.360914 | 0.65099 | 12.08% |
| **4a**-4 | -886.857709 | 0.325014 | -556299.266259 | 0.745644 | 10.30% |
| **4a**-5 | -886.856411 | 0.324034 | -556299.066362 | 0.945542 | 7.35% |
| **4a**-6 | -886.856549 | 0.324597 | -556298.799916 | 1.211987 | 4.68% |
| **4a**-7 | -886.856962 | 0.32514 | -556298.718372 | 1.293532 | 4.08% |
| **4a**-8 | -886.856326 | 0.324658 | -556298.621529 | 1.390375 | 3.47% |
| **4a**-9 | -886.856593 | 0.325266 | -556298.407639 | 1.604265 | 2.41% |

*^a^*Electronic energy obtained at M06-2X-D3/6-311+G(2d,p) level of theory; *^b^*Thermal correction to Gibbs free energy obtained at B3LYP-D3BJ/6-31G(d) level of theory; *^c^*Gibbs free energy (E + C); *^d^*The relative Gibbs free energy; *^e^*The Boltzmann distribution of each conformer.

**Table S34.** Atomic coordinates (Å) of **4a**-1 obtained at the B3LYP-D3BJ/6-31G(d) level of theory in the gas phase.

| C | 0.251918 | -0.984989 | -0.208021 | H | -0.009782 | -3.100844 | -0.293085 |
| --- | --- | --- | --- | --- | --- | --- | --- |
| C | -0.513113 | -2.238470 | 0.150183 | H | 0.018744 | 1.827405 | -1.729696 |
| C | -1.987646 | -2.201246 | -0.251752 | H | 2.321358 | 2.092709 | -1.487697 |
| C | -2.643272 | -0.874587 | 0.133854 | H | 2.346703 | 1.579871 | 0.181647 |
| C | -1.828742 | 0.260769 | -0.492931 | H | 2.008839 | -0.249273 | -2.237881 |
| C | -0.350565 | 0.210601 | -0.430530 | H | 3.439965 | -0.126649 | -1.196999 |
| C | 0.373419 | 1.512550 | -0.738522 | H | -1.133087 | 2.774175 | 0.111925 |
| C | 1.906129 | 1.354004 | -0.795086 | H | 1.690345 | 3.959453 | 0.022571 |
| C | 2.349679 | -0.043006 | -1.211934 | H | 0.200014 | 4.796707 | 0.467404 |
| C | 1.765717 | -1.119351 | -0.293333 | H | 0.427545 | 4.225390 | -1.193577 |
| C | -0.054151 | 2.649513 | 0.240376 | H | -0.293393 | 1.376355 | 2.005082 |
| C | 0.606584 | 3.980337 | -0.139957 | H | 1.272544 | 2.200201 | 1.931319 |
| C | 0.203108 | 2.307926 | 1.713515 | H | -0.178814 | 3.105114 | 2.360844 |
| C | 2.393450 | -1.091930 | 1.116528 | H | 2.044603 | -2.416372 | -1.748639 |
| C | -2.691286 | -0.656475 | 1.658797 | H | 2.214584 | -0.126652 | 1.594285 |
| O | -2.433515 | 1.184379 | -1.033874 | H | 1.906477 | -1.868221 | 1.726322 |
| O | -3.957293 | -0.837275 | -0.385116 | H | -4.043799 | 0.056883 | -0.772720 |
| O | 2.147001 | -2.422261 | -0.783856 | H | -1.699387 | -0.680178 | 2.121530 |
| O | 3.791166 | -1.285963 | 1.057120 | H | -3.150556 | 0.313090 | 1.875555 |
| H | -2.538528 | -3.029933 | 0.207059 | H | -3.310982 | -1.438477 | 2.108578 |
| H | -2.083034 | -2.313794 | -1.338595 | H | 3.909277 | -2.078534 | 0.505174 |
| H | -0.425879 | -2.383344 | 1.238279 | - | - | - | - |

**Table S35.** Atomic coordinates (Å) of **4a**-2 obtained at the B3LYP-D3BJ/6-31G(d) level of theory in the gas phase.

| C | 0.281732 | -0.951148 | -0.339619 | H | -0.153878 | -3.025939 | -0.617494 |
| --- | --- | --- | --- | --- | --- | --- | --- |
| C | -0.573401 | -2.169570 | -0.082284 | H | 0.240808 | 1.972414 | -1.660750 |
| C | -2.048175 | -1.989378 | -0.441173 | H | 2.553664 | 2.059567 | -1.431755 |
| C | -2.591320 | -0.648521 | 0.053411 | H | 2.553222 | 1.454547 | 0.206578 |
| C | -1.704864 | 0.457627 | -0.523525 | H | 2.071229 | -0.214254 | -2.307963 |
| C | -0.234803 | 0.296267 | -0.478925 | H | 3.520263 | -0.248146 | -1.299488 |
| C | 0.575694 | 1.565772 | -0.696578 | H | -0.842847 | 2.854704 | 0.254422 |
| C | 2.094625 | 1.311507 | -0.777705 | H | 0.613818 | 4.764454 | 0.732540 |
| C | 2.436566 | -0.087869 | -1.278331 | H | 0.788589 | 4.298551 | -0.967275 |
| C | 1.789800 | -1.172247 | -0.412045 | H | 2.045747 | 3.873339 | 0.209122 |
| C | 0.227470 | 2.657569 | 0.361234 | H | 1.541340 | 2.043921 | 2.010487 |
| C | 0.963410 | 3.969750 | 0.063985 | H | 0.125342 | 2.967112 | 2.508821 |
| C | 0.475639 | 2.203543 | 1.805461 | H | -0.052285 | 1.272100 | 2.034961 |
| C | 2.413160 | -1.225138 | 0.999616 | H | 1.793041 | -2.504977 | -1.863087 |
| C | -2.581087 | -0.535618 | 1.590337 | H | 3.508494 | -1.244029 | 0.879863 |
| O | -2.245117 | 1.453076 | -1.002590 | H | 2.140472 | -0.336485 | 1.571004 |
| O | -3.910937 | -0.474586 | -0.422077 | H | -3.931216 | 0.442907 | -0.761366 |
| O | 2.111205 | -2.469269 | -0.947349 | H | -1.583665 | -0.679392 | 2.017268 |
| O | 1.957914 | -2.341009 | 1.739606 | H | -2.948892 | 0.451397 | 1.888088 |
| H | -2.651497 | -2.804380 | -0.025767 | H | -3.255380 | -1.292262 | 2.003442 |
| H | -2.178601 | -2.016027 | -1.530134 | H | 2.103788 | -3.110605 | 1.163778 |
| H | -0.452747 | -2.419495 | 0.980827 | - | - | - | - |

**Table S36.** Atomic coordinates (Å) of **4a**-3 obtained at the B3LYP-D3BJ/6-31G(d) level of theory in the gas phase.

| C | 0.360372 | -0.844374 | -0.205606 | H | 0.123952 | -2.934488 | -0.554663 |
| --- | --- | --- | --- | --- | --- | --- | --- |
| C | -0.404172 | -2.138203 | -0.024287 | H | -0.159111 | 2.242185 | -1.325330 |
| C | -1.858355 | -2.072762 | -0.492361 | H | 1.578296 | 0.867625 | -2.323340 |
| C | -2.555321 | -0.803178 | -0.003029 | H | 2.370216 | 2.233445 | -1.556088 |
| C | -1.741639 | 0.390903 | -0.503944 | H | 3.567344 | 0.103902 | -0.969520 |
| C | -0.264217 | 0.344625 | -0.405065 | H | 2.869277 | 0.854334 | 0.455304 |
| C | 0.468597 | 1.648863 | -0.650871 | H | 1.259891 | 1.905071 | 1.350225 |
| C | 1.797647 | 1.325457 | -1.348617 | H | -0.537909 | 3.374731 | 2.245460 |
| C | 2.616954 | 0.364529 | -0.493147 | H | -1.174015 | 1.824306 | 1.690919 |
| C | 1.885422 | -0.960930 | -0.200082 | H | -1.400247 | 3.267973 | 0.696970 |
| C | 0.639358 | 2.485703 | 0.653513 | H | 1.409016 | 4.420131 | 1.285552 |
| C | -0.697582 | 2.751147 | 1.358590 | H | 2.373652 | 3.678313 | 0.007205 |
| C | 1.351430 | 3.816335 | 0.373143 | H | 0.800998 | 4.398600 | -0.376679 |
| C | 2.436972 | -1.520004 | 1.130832 | H | 1.899102 | -1.712633 | -2.025614 |
| C | -2.651211 | -0.735332 | 1.533256 | H | 3.529686 | -1.377552 | 1.111608 |
| O | -2.338452 | 1.354963 | -0.977148 | H | 2.034254 | -0.937833 | 1.965311 |
| O | -3.854626 | -0.739864 | -0.557087 | H | -3.949809 | 0.185771 | -0.858026 |
| O | 2.274634 | -1.964656 | -1.167348 | H | -3.156038 | 0.190038 | 1.827545 |
| O | 2.108871 | -2.871905 | 1.361885 | H | -3.250142 | -1.580237 | 1.887283 |
| H | -2.415831 | -2.950993 | -0.148067 | H | -1.671211 | -0.768198 | 2.019885 |
| H | -1.904005 | -2.072813 | -1.588670 | H | 2.320897 | -3.333269 | 0.532532 |
| H | -0.347203 | -2.423400 | 1.033833 | - | - | - | - |

**Table S37.** Atomic coordinates (Å) of **4a**-4 obtained at the B3LYP-D3BJ/6-31G(d) level of theory in the gas phase.

| C | 0.325757 | -0.875093 | -0.100192 | H | 0.247791 | -2.997812 | -0.264773 |
| --- | --- | --- | --- | --- | --- | --- | --- |
| C | -0.350010 | -2.198748 | 0.182876 | H | -0.369975 | 2.100239 | -1.403938 |
| C | -1.793829 | -2.272026 | -0.316165 | H | 1.465940 | 0.803831 | -2.307303 |
| C | -2.586701 | -1.023214 | 0.072338 | H | 2.162587 | 2.243034 | -1.589469 |
| C | -1.847625 | 0.196715 | -0.483345 | H | 3.491187 | 0.247980 | -0.875327 |
| C | -0.371720 | 0.256851 | -0.370279 | H | 2.692728 | 0.963366 | 0.503341 |
| C | 0.282545 | 1.587503 | -0.688187 | H | 1.037787 | 2.013641 | 1.296998 |
| C | 1.642393 | 1.312408 | -1.347916 | H | -1.711984 | 3.134659 | 0.557063 |
| C | 2.507026 | 0.440536 | -0.440341 | H | -0.871121 | 3.402879 | 2.097864 |
| C | 1.851124 | -0.920204 | -0.144902 | H | -1.385699 | 1.777715 | 1.641372 |
| C | 0.380936 | 2.507278 | 0.567498 | H | 1.005205 | 4.523312 | 1.093265 |
| C | -0.977265 | 2.714868 | 1.251645 | H | 2.027636 | 3.785940 | -0.140991 |
| C | 0.995993 | 3.868630 | 0.214818 | H | 0.408392 | 4.368155 | -0.565880 |
| C | 2.449247 | -1.529027 | 1.135733 | H | 2.028314 | -1.478058 | -2.029327 |
| C | -2.729444 | -0.860808 | 1.598514 | H | 2.230490 | -0.879545 | 1.988660 |
| O | -2.502801 | 1.089256 | -1.015044 | H | 1.995736 | -2.511823 | 1.324138 |
| O | -3.871411 | -1.089609 | -0.513791 | H | -4.029753 | -0.190604 | -0.864804 |
| O | 2.227208 | -1.878736 | -1.168034 | H | -3.317143 | 0.036563 | 1.814787 |
| O | 3.855620 | -1.631827 | 1.033451 | H | -3.265025 | -1.726984 | 1.999639 |
| H | -2.298184 | -3.161965 | 0.076449 | H | -1.763812 | -0.773277 | 2.106817 |
| H | -1.808819 | -2.348772 | -1.410138 | H | 4.014810 | -2.103336 | 0.197824 |
| H | -0.326577 | -2.375819 | 1.268524 | - | - | - | - |

**Table S38.** Atomic coordinates (Å) of **4a**-5 obtained at the B3LYP-D3BJ/6-31G(d) level of theory in the gas phase.

| C | 0.370830 | -0.817012 | -0.225851 | H | 0.235756 | -2.905087 | -0.625783 |
| --- | --- | --- | --- | --- | --- | --- | --- |
| C | -0.313692 | -2.155248 | -0.051467 | H | -0.259309 | 2.101161 | -1.555204 |
| C | -1.789258 | -2.164530 | -0.453316 | H | 1.788422 | 1.173511 | -2.167132 |
| C | -2.528275 | -0.935164 | 0.073128 | H | 2.266877 | 2.508719 | -1.140895 |
| C | -1.804318 | 0.298809 | -0.464057 | H | 3.559371 | 0.441251 | -0.585235 |
| C | -0.322147 | 0.332340 | -0.432167 | H | 2.570279 | 0.937846 | 0.781619 |
| C | 0.296766 | 1.689195 | -0.704105 | H | -1.081512 | 2.700510 | 0.552008 |
| C | 1.763127 | 1.537474 | -1.129598 | H | 1.525026 | 4.218225 | 0.030367 |
| C | 2.525562 | 0.557360 | -0.243078 | H | 0.080830 | 4.832421 | 0.836619 |
| C | 1.898041 | -0.841428 | -0.220636 | H | 0.018167 | 4.417132 | -0.884200 |
| C | 0.010092 | 2.692548 | 0.457586 | H | 0.367383 | 1.251941 | 2.071151 |
| C | 0.434434 | 4.118058 | 0.084593 | H | 1.663818 | 2.449173 | 1.878542 |
| C | 0.579728 | 2.298706 | 1.826893 | H | 0.133118 | 2.919823 | 2.611604 |
| C | 2.491986 | -1.601499 | 0.988502 | H | 1.922944 | -1.251472 | -2.151363 |
| C | -2.550741 | -0.864553 | 1.612393 | H | 3.559103 | -1.333504 | 1.047433 |
| O | -2.472722 | 1.237455 | -0.891198 | H | 2.009643 | -1.258450 | 1.909360 |
| O | -3.854793 | -0.941298 | -0.416444 | H | -4.019132 | -0.019894 | -0.699412 |
| O | 2.346378 | -1.615560 | -1.358470 | H | -3.077001 | 0.041521 | 1.928875 |
| O | 2.330279 | -3.000707 | 0.920299 | H | -3.096471 | -1.731509 | 1.997470 |
| H | -2.282834 | -3.072644 | -0.089949 | H | -1.547409 | -0.854116 | 2.049915 |
| H | -1.884485 | -2.163261 | -1.546393 | H | 2.578042 | -3.241777 | 0.011081 |
| H | -0.196572 | -2.470970 | 0.993105 | - | - | - | - |

**Table S39.** Atomic coordinates (Å) of **4a**-6 obtained at the B3LYP-D3BJ/6-31G(d) level of theory in the gas phase.

| C | 0.276888 | -0.882029 | -0.168252 | H | 0.152984 | -2.984206 | -0.467397 |
| --- | --- | --- | --- | --- | --- | --- | --- |
| C | -0.407577 | -2.210090 | 0.066674 | H | -0.343595 | 1.990816 | -1.589382 |
| C | -1.877547 | -2.237824 | -0.354120 | H | 1.690899 | 1.048115 | -2.196441 |
| C | -2.621016 | -0.992288 | 0.128207 | H | 2.191807 | 2.392564 | -1.198339 |
| C | -1.893605 | 0.231688 | -0.431058 | H | 3.480128 | 0.337572 | -0.595536 |
| C | -0.411944 | 0.262801 | -0.404271 | H | 2.496242 | 0.855347 | 0.754842 |
| C | 0.216942 | 1.603018 | -0.729887 | H | -1.139143 | 2.660149 | 0.514441 |
| C | 1.680024 | 1.426143 | -1.162502 | H | 0.033170 | 4.798273 | 0.703845 |
| C | 2.446396 | 0.459634 | -0.261980 | H | -0.066580 | 4.324015 | -1.000094 |
| C | 1.800464 | -0.930152 | -0.212850 | H | 1.459004 | 4.153260 | -0.110848 |
| C | -0.049329 | 2.647141 | 0.400320 | H | 0.122047 | 2.951594 | 2.541672 |
| C | 0.369667 | 4.057818 | -0.030538 | H | 0.333573 | 1.263447 | 2.056479 |
| C | 0.547174 | 2.299352 | 1.770335 | H | 1.633025 | 2.442869 | 1.791792 |
| C | 2.399920 | -1.739693 | 0.952328 | H | 1.964448 | -1.184504 | -2.162881 |
| C | -2.663520 | -0.877126 | 1.664669 | H | 2.185647 | -1.237355 | 1.900569 |
| O | -2.562441 | 1.162075 | -0.874738 | H | 1.949200 | -2.740447 | 0.981053 |
| O | -3.940946 | -1.014794 | -0.377516 | H | -4.107476 | -0.098846 | -0.676707 |
| O | 2.178977 | -1.710334 | -1.376113 | H | -3.204157 | 0.031953 | 1.945955 |
| O | 3.806353 | -1.826965 | 0.830413 | H | -3.203915 | -1.738100 | 2.070349 |
| H | -2.376060 | -3.134719 | 0.029821 | H | -1.666201 | -0.838430 | 2.114709 |
| H | -1.955064 | -2.268708 | -1.447694 | H | 3.961330 | -2.152450 | -0.072989 |
| H | -0.324475 | -2.462425 | 1.133953 | - | - | - | - |

**Table S40.** Atomic coordinates (Å) of **4a**-7 obtained at the B3LYP-D3BJ/6-31G(d) level of theory in the gas phase.

| C | 0.061275 | -1.030624 | -0.205492 | H | -0.752138 | -2.996047 | -0.328846 |
| --- | --- | --- | --- | --- | --- | --- | --- |
| C | -1.023810 | -2.035797 | 0.117786 | H | 0.414961 | 2.021880 | -1.480714 |
| C | -2.416949 | -1.601897 | -0.339701 | H | 1.576694 | 0.137029 | -2.492127 |
| C | -2.714506 | -0.153523 | 0.053205 | H | 2.788558 | 1.234057 | -1.852271 |
| C | -1.613791 | 0.734845 | -0.533444 | H | 3.334872 | -1.142061 | -1.213342 |
| C | -0.207289 | 0.272166 | -0.468202 | H | 3.014636 | -0.149009 | 0.201994 |
| C | 0.868821 | 1.281572 | -0.812398 | H | 1.762284 | 1.307627 | 1.157354 |
| C | 1.991292 | 0.545296 | -1.559394 | H | 0.590550 | 3.335369 | 2.025723 |
| C | 2.553432 | -0.578764 | -0.691825 | H | -0.549376 | 2.079080 | 1.538831 |
| C | 1.482997 | -1.598446 | -0.250866 | H | -0.293899 | 3.478382 | 0.492790 |
| C | 1.358274 | 2.047545 | 0.454425 | H | 2.751177 | 3.626384 | 0.998306 |
| C | 0.208489 | 2.774903 | 1.165241 | H | 3.378444 | 2.551476 | -0.252223 |
| C | 2.472431 | 3.045384 | 0.112173 | H | 2.139613 | 3.752466 | -0.658489 |
| C | 1.859728 | -2.177047 | 1.127734 | H | 1.335903 | -2.417337 | -2.024581 |
| C | -2.751188 | 0.052107 | 1.580694 | H | 1.198727 | -3.021752 | 1.363439 |
| O | -1.930455 | 1.805734 | -1.043200 | H | 2.883470 | -2.556836 | 1.075547 |
| O | -3.957982 | 0.229888 | -0.498475 | H | -3.808628 | 1.132913 | -0.842930 |
| O | 1.483549 | -2.744092 | -1.122492 | H | -2.972384 | 1.100599 | 1.802203 |
| O | 1.847980 | -1.192261 | 2.148857 | H | -3.549572 | -0.565369 | 2.003766 |
| H | -3.187797 | -2.259267 | 0.077532 | H | -1.806028 | -0.211439 | 2.067311 |
| H | -2.490823 | -1.671148 | -1.431785 | H | 0.952718 | -0.819179 | 2.190201 |
| H | -1.031922 | -2.208295 | 1.205155 | - | - | - | - |

**Table S41.** Atomic coordinates (Å) of **4a**-8 obtained at the B3LYP-D3BJ/6-31G(d) level of theory in the gas phase.

| C | 0.491342 | -0.885902 | -0.326319 | H | 0.401503 | -2.979800 | -0.685295 |
| --- | --- | --- | --- | --- | --- | --- | --- |
| C | -0.170255 | -2.227947 | -0.130929 | H | -0.186868 | 2.063042 | -1.584599 |
| C | -1.638891 | -2.255377 | -0.555023 | H | 1.804926 | 0.975844 | -2.345016 |
| C | -2.393140 | -1.044236 | -0.008437 | H | 2.346065 | 2.380481 | -1.438338 |
| C | -1.685737 | 0.224209 | -0.495542 | H | 3.675686 | 0.382808 | -0.720942 |
| C | -0.208198 | 0.257947 | -0.518027 | H | 2.642759 | 0.920687 | 0.608220 |
| C | 0.422953 | 1.607203 | -0.797699 | H | 1.443783 | 2.927436 | 0.576973 |
| C | 1.846275 | 1.410701 | -1.334683 | H | 0.056807 | 2.720176 | 2.560136 |
| C | 2.633654 | 0.494920 | -0.401059 | H | 0.514425 | 1.094600 | 2.017661 |
| C | 2.018160 | -0.908508 | -0.333527 | H | -1.119243 | 1.714055 | 1.712909 |
| C | 0.405089 | 2.601188 | 0.425627 | H | -0.044567 | 4.338991 | -0.817618 |
| C | -0.059345 | 1.987494 | 1.752671 | H | -1.466661 | 3.568736 | -0.094832 |
| C | -0.423310 | 3.846495 | 0.086320 | H | -0.393378 | 4.575684 | 0.904533 |
| C | 2.556736 | -1.655956 | 0.892830 | H | 2.192162 | -1.258291 | -2.252010 |
| C | -2.430758 | -1.020276 | 1.532894 | H | 2.303650 | -2.720254 | 0.807565 |
| O | -2.372735 | 1.190020 | -0.822507 | H | 3.652204 | -1.566347 | 0.893620 |
| O | -3.714589 | -1.054124 | -0.514762 | H | -3.908688 | -0.116370 | -0.711451 |
| O | 2.461679 | -1.716563 | -1.440265 | H | -1.433097 | -0.976269 | 1.980282 |
| O | 1.976837 | -1.068998 | 2.052406 | H | -2.998530 | -0.146542 | 1.867618 |
| H | -2.127387 | -3.175300 | -0.214820 | H | -2.944631 | -1.919684 | 1.887251 |
| H | -1.717039 | -2.233358 | -1.648834 | H | 2.387474 | -1.469318 | 2.832421 |
| H | -0.078347 | -2.495424 | 0.931009 | - | - | - | - |

**Table S42.** Atomic coordinates (Å) of **4a**-9 obtained at the B3LYP-D3BJ/6-31G(d) level of theory in the gas phase.

| C | 0.012390 | -0.984624 | -0.342656 | H | -0.882933 | -2.870721 | -0.740972 |
| --- | --- | --- | --- | --- | --- | --- | --- |
| C | -1.103858 | -1.981489 | -0.140721 | H | 0.485716 | 1.993119 | -1.601546 |
| C | -2.493279 | -1.439654 | -0.480125 | H | 2.011134 | 0.385024 | -2.315809 |
| C | -2.696534 | -0.037939 | 0.096257 | H | 2.989941 | 1.404789 | -1.284617 |
| C | -1.594459 | 0.859804 | -0.467536 | H | 3.424988 | -1.020429 | -0.825601 |
| C | -0.207541 | 0.342280 | -0.506829 | H | 2.752954 | -0.215437 | 0.585638 |
| C | 0.869222 | 1.372756 | -0.782039 | H | 0.009099 | 2.759616 | 0.572491 |
| C | 2.157714 | 0.694237 | -1.270397 | H | 1.568981 | 4.035984 | -0.882762 |
| C | 2.524833 | -0.531800 | -0.436404 | H | 2.955073 | 3.263833 | -0.089593 |
| C | 1.411786 | -1.588219 | -0.407623 | H | 1.883720 | 4.315672 | 0.837927 |
| C | 1.019262 | 2.366481 | 0.412693 | H | 0.938182 | 0.806721 | 1.951342 |
| C | 1.907777 | 3.560158 | 0.044505 | H | 2.551073 | 1.526662 | 1.736299 |
| C | 1.474655 | 1.737554 | 1.737439 | H | 1.288173 | 2.428811 | 2.566999 |
| C | 1.642457 | -2.556421 | 0.769501 | H | 1.374250 | -1.865887 | -2.348584 |
| C | -2.608808 | -0.010795 | 1.635464 | H | 1.001792 | -3.431376 | 0.648902 |
| O | -1.887053 | 1.992308 | -0.845122 | H | 2.685799 | -2.900894 | 0.738239 |
| O | -3.953381 | 0.460687 | -0.316986 | H | -3.777223 | 1.385008 | -0.582987 |
| O | 1.492170 | -2.434105 | -1.570603 | H | -1.650399 | -0.383305 | 2.010428 |
| O | 1.307613 | -1.985692 | 2.026500 | H | -2.748003 | 1.015555 | 1.989713 |
| H | -3.274882 | -2.107604 | -0.101514 | H | -3.415258 | -0.626918 | 2.045522 |
| H | -2.622783 | -1.376089 | -1.567536 | H | 1.956092 | -1.295215 | 2.230471 |
| H | -1.073987 | -2.306470 | 0.908075 | - | - | - | - |

**Table S43.** Conformational analysis of the B3LYP-D3BJ/6-31G(d) optimized conformers of **4b** in the gas phase (T=298.15 K)

| Conformer | E (Hartree) | C (Hartree) | G (kcal/mol) | ΔG (kcal/mol) | Population |
| --- | --- | --- | --- | --- | --- |
| **4b**-1 | -886.85782 | 0.325422 | -556299.079687 | 0.0 | 51.89% |
| **4b**-2 | -886.856276 | 0.325199 | -556298.250925 | 0.828762 | 12.80% |
| **4b**-3 | -886.856312 | 0.325282 | -556298.22152 | 0.858166 | 12.18% |
| **4b**-4 | -886.855362 | 0.324636 | -556298.030524 | 1.049162 | 8.82% |
| **4b**-5 | -886.855333 | 0.32548 | -556297.482692 | 1.596994 | 3.50% |
| **4b**-6 | -886.855061 | 0.325336 | -556297.402469 | 1.677218 | 3.05% |
| **4b**-7 | -886.855465 | 0.325795 | -556297.368045 | 1.711642 | 2.88% |
| **4b**-8 | -886.854131 | 0.324547 | -556297.313681 | 1.766006 | 2.63% |
| **4b**-9 | -886.854024 | 0.324584 | -556297.223321 | 1.856365 | 2.26% |

*^a^*Electronic energy obtained at M06-2X-D3/6-311+G(2d,p) level of theory; *^b^*Thermal correction to Gibbs free energy obtained at B3LYP-D3BJ/6-31G(d) level of theory; *^c^*Gibbs free energy (E + C); *^d^*The relative Gibbs free energy; *^e^*The Boltzmann distribution of each conformer.

**Table S44.** Atomic coordinates (Å) of **4b**-1 obtained at the B3LYP-D3BJ/6-31G(d) level of theory in the gas phase.

| C | 0.390025 | -0.865139 | 0.098028 | H | 0.462856 | -3.006066 | 0.220117 |
| --- | --- | --- | --- | --- | --- | --- | --- |
| C | -0.222999 | -2.193995 | 0.479473 | H | -0.155255 | 1.796208 | -1.696646 |
| C | -1.599644 | -2.437366 | -0.140010 | H | 2.087604 | 1.244267 | -1.712499 |
| C | -2.503941 | -1.213480 | 0.003906 | H | 2.119982 | 2.591858 | -0.603013 |
| C | -1.789444 | -0.009939 | -0.615746 | H | 3.479661 | 0.667552 | 0.235475 |
| C | -0.343340 | 0.180952 | -0.357853 | H | 2.149483 | 1.035954 | 1.338935 |
| C | 0.208563 | 1.555590 | -0.690528 | H | -1.513405 | 2.471256 | 0.181607 |
| C | 1.748775 | 1.569229 | -0.721792 | H | -0.732231 | 4.795529 | 0.276386 |
| C | 2.390298 | 0.667033 | 0.335001 | H | -0.520024 | 4.137688 | -1.354762 |
| C | 1.901249 | -0.777033 | 0.224267 | H | 0.876454 | 4.327118 | -0.278607 |
| C | -0.432936 | 2.641558 | 0.231368 | H | 1.031351 | 2.829736 | 1.848531 |
| C | -0.184628 | 4.052919 | -0.314994 | H | -0.623468 | 3.223665 | 2.314556 |
| C | -0.017458 | 2.545130 | 1.703701 | H | -0.155265 | 1.531094 | 2.096265 |
| C | 2.569313 | -1.496714 | -0.972985 | H | 2.231587 | -1.005778 | 2.151248 |
| C | -2.821241 | -0.876488 | 1.474861 | H | 2.138145 | -2.503555 | -1.072773 |
| O | -2.444501 | 0.772292 | -1.300728 | H | 2.368681 | -0.951161 | -1.897905 |
| O | -3.710779 | -1.443210 | -0.694938 | H | -3.889216 | -0.602412 | -1.161782 |
| O | 2.360365 | -1.545894 | 1.355998 | H | -3.471340 | 0.003108 | 1.514300 |
| O | 3.970565 | -1.555013 | -0.808647 | H | -3.354766 | -1.719284 | 1.925290 |
| H | -2.084740 | -3.307560 | 0.316291 | H | -1.922703 | -0.668082 | 2.064848 |
| H | -1.496434 | -2.649615 | -1.211000 | H | 4.101483 | -1.890913 | 0.095449 |
| H | -0.289171 | -2.229552 | 1.576013 | - | - | - | - |

**Table S45.** Atomic coordinates (Å) of **4b**-2 obtained at the B3LYP-D3BJ/6-31G(d) level of theory in the gas phase.

| C | 0.607430 | -0.741097 | -0.205103 | H | 0.981619 | -2.854210 | -0.351078 |
| --- | --- | --- | --- | --- | --- | --- | --- |
| C | 0.247319 | -2.180101 | 0.092793 | H | -0.524035 | 1.909458 | -1.669471 |
| C | -1.161785 | -2.578127 | -0.349709 | H | 1.594313 | 2.839177 | -1.498010 |
| C | -2.202213 | -1.527345 | 0.034375 | H | 1.795231 | 2.411271 | 0.182977 |
| C | -1.756403 | -0.192343 | -0.562393 | H | 2.088036 | 0.485855 | -2.165266 |
| C | -0.333296 | 0.207982 | -0.449775 | H | 3.369016 | 1.114097 | -1.118292 |
| C | -0.059099 | 1.682981 | -0.701342 | H | -1.867991 | 2.339648 | 0.243323 |
| C | 1.441911 | 2.027439 | -0.780139 | H | -1.256174 | 4.674590 | 0.679805 |
| C | 2.311015 | 0.831831 | -1.147173 | H | -0.927873 | 4.278805 | -1.015141 |
| C | 2.081174 | -0.332250 | -0.174951 | H | 0.405115 | 4.391484 | 0.148642 |
| C | -0.804019 | 2.574119 | 0.341209 | H | 0.654961 | 2.532460 | 1.983098 |
| C | -0.632484 | 4.063106 | 0.018118 | H | -0.995920 | 2.867048 | 2.488651 |
| C | -0.394843 | 2.275129 | 1.789971 | H | -0.546352 | 1.219334 | 2.043995 |
| C | 3.073612 | -1.464337 | -0.493221 | H | 1.701835 | 0.550131 | 1.554934 |
| C | -2.344630 | -1.361950 | 1.560264 | H | 2.779276 | -1.976873 | -1.414667 |
| O | -2.595369 | 0.517096 | -1.112824 | H | 4.057035 | -1.001210 | -0.667705 |
| O | -3.454598 | -1.883560 | -0.515105 | H | -3.801199 | -1.054616 | -0.901424 |
| O | 2.441228 | 0.050350 | 1.176180 | H | -2.703971 | -2.304657 | 1.984218 |
| O | 3.151788 | -2.430716 | 0.533816 | H | -1.401133 | -1.093623 | 2.046383 |
| H | -1.441778 | -3.546044 | 0.080719 | H | -3.082502 | -0.583472 | 1.778465 |
| H | -1.193274 | -2.690422 | -1.440392 | H | 3.164743 | -1.910550 | 1.356763 |
| H | 0.364502 | -2.333263 | 1.175383 | - | - | - | - |

**Table S46.** Atomic coordinates (Å) of **4b**-3 obtained at the B3LYP-D3BJ/6-31G(d) level of theory in the gas phase.

| C | 0.531827 | -0.783402 | 0.147127 | H | 0.842569 | -2.905012 | 0.299055 |
| --- | --- | --- | --- | --- | --- | --- | --- |
| C | 0.056184 | -2.174311 | 0.505477 | H | -0.419188 | 1.899305 | -1.514633 |
| C | -1.240732 | -2.573723 | -0.198785 | H | 1.862537 | 1.243931 | -1.810210 |
| C | -2.301410 | -1.479801 | -0.077496 | H | 1.961213 | 2.724147 | -0.879872 |
| C | -1.713548 | -0.168065 | -0.609423 | H | 3.471886 | 1.028740 | 0.128016 |
| C | -0.311022 | 0.187464 | -0.288740 | H | 2.145554 | 1.376786 | 1.240757 |
| C | 0.106608 | 1.616484 | -0.595988 | H | 0.438443 | 2.615373 | 1.292475 |
| C | 1.624755 | 1.684057 | -0.834459 | H | -1.692186 | 1.404245 | 1.668283 |
| C | 2.390281 | 0.940554 | 0.262225 | H | -2.488825 | 2.394538 | 0.435252 |
| C | 2.027687 | -0.544951 | 0.282529 | H | -1.878055 | 3.141760 | 1.922391 |
| C | -0.321762 | 2.650826 | 0.498571 | H | 0.626696 | 4.332289 | -0.560945 |
| C | -1.674684 | 2.375849 | 1.165274 | H | -1.110750 | 4.157529 | -0.854677 |
| C | -0.326944 | 4.068073 | -0.092624 | H | -0.530723 | 4.812473 | 0.685365 |
| C | 2.792477 | -1.293115 | -0.839361 | H | 2.298886 | -0.603328 | 2.231540 |
| C | -2.755560 | -1.259428 | 1.379442 | H | 2.457040 | -2.338791 | -0.877021 |
| O | -2.428094 | 0.551755 | -1.302792 | H | 2.577707 | -0.835042 | -1.807637 |
| O | -3.421185 | -1.832268 | -0.865494 | H | -3.685666 | -0.999560 | -1.305028 |
| O | 2.526033 | -1.175355 | 1.481868 | H | -3.212471 | -2.182896 | 1.748542 |
| O | 4.187507 | -1.215161 | -0.633008 | H | -1.930064 | -0.982938 | 2.043248 |
| H | -1.634292 | -3.512364 | 0.207397 | H | -3.506948 | -0.464929 | 1.412650 |
| H | -1.051299 | -2.737268 | -1.266514 | H | 4.316321 | -1.466075 | 0.298575 |
| H | -0.077411 | -2.213732 | 1.595798 | - | - | - | - |

**Table S47.** Atomic coordinates (Å) of **4b**-4 obtained at the B3LYP-D3BJ/6-31G(d) level of theory in the gas phase.

| C | 0.679415 | -0.637942 | 0.103506 | H | 1.346341 | -2.655067 | 0.123093 |
| --- | --- | --- | --- | --- | --- | --- | --- |
| C | 0.476629 | -2.091341 | 0.467530 | H | -0.594968 | 1.718840 | -1.738278 |
| C | -0.806534 | -2.703414 | -0.096148 | H | 1.716038 | 1.814443 | -1.740075 |
| C | -2.005671 | -1.772163 | 0.072212 | H | 1.358990 | 3.156672 | -0.678792 |
| C | -1.659974 | -0.436349 | -0.588437 | H | 3.173895 | 1.752311 | 0.285224 |
| C | -0.322749 | 0.155136 | -0.354376 | H | 1.752670 | 1.698298 | 1.319515 |
| C | -0.184401 | 1.621777 | -0.725892 | H | -2.102902 | 2.051302 | 0.103231 |
| C | 1.289287 | 2.067596 | -0.761930 | H | -0.309955 | 4.487236 | -0.362354 |
| C | 2.133370 | 1.412431 | 0.333165 | H | -2.002282 | 4.496593 | 0.136722 |
| C | 2.105458 | -0.113384 | 0.237144 | H | -1.562009 | 3.893175 | -1.469468 |
| C | -1.111934 | 2.512789 | 0.160629 | H | 0.208973 | 3.140490 | 1.792284 |
| C | -1.250127 | 3.925715 | -0.419476 | H | -1.498699 | 3.065915 | 2.224373 |
| C | -0.714909 | 2.569971 | 1.640725 | H | -0.572505 | 1.567644 | 2.060775 |
| C | 2.989698 | -0.595163 | -0.944408 | H | 2.304525 | -0.385422 | 2.178481 |
| C | -2.353665 | -1.507134 | 1.550371 | H | 2.480261 | -0.419422 | -1.894396 |
| O | -2.512175 | 0.116068 | -1.281050 | H | 3.912819 | 0.006246 | -0.928171 |
| O | -3.128748 | -2.331766 | -0.578976 | H | -3.527895 | -1.584705 | -1.068289 |
| O | 2.763250 | -0.690614 | 1.380529 | H | -3.209024 | -0.826636 | 1.608686 |
| O | 3.289644 | -1.974563 | -0.885815 | H | -2.634700 | -2.453353 | 2.023189 |
| H | -1.019742 | -3.666734 | 0.380711 | H | -1.520757 | -1.065077 | 2.106773 |
| H | -0.685060 | -2.895984 | -1.168900 | H | 3.619508 | -2.127127 | 0.016339 |
| H | 0.487350 | -2.173781 | 1.564139 | - | - | - | - |

**Table S48.** Atomic coordinates (Å) of **4b**-5 obtained at the B3LYP-D3BJ/6-31G(d) level of theory in the gas phase.

| C | 0.608077 | -0.722309 | 0.054932 | H | 1.053813 | -2.821690 | 0.143544 |
| --- | --- | --- | --- | --- | --- | --- | --- |
| C | 0.231941 | -2.146416 | 0.397649 | H | -0.505471 | 1.868501 | -1.617570 |
| C | -1.060699 | -2.608390 | -0.275848 | H | 1.813234 | 1.372053 | -1.873226 |
| C | -2.182307 | -1.590688 | -0.081715 | H | 1.770482 | 2.879950 | -0.974940 |
| C | -1.707840 | -0.221969 | -0.582951 | H | 3.391169 | 1.327521 | 0.121879 |
| C | -0.303458 | 0.188439 | -0.368394 | H | 1.995473 | 1.573033 | 1.179365 |
| C | 0.018070 | 1.637038 | -0.684636 | H | 0.358661 | 3.250716 | 0.712482 |
| C | 1.525402 | 1.813827 | -0.911394 | H | -1.316926 | 2.808884 | 2.404364 |
| C | 2.316443 | 1.150297 | 0.215893 | H | -0.388262 | 1.324712 | 2.137927 |
| C | 2.076969 | -0.362679 | 0.230688 | H | -2.018904 | 1.487107 | 1.468781 |
| C | -0.511205 | 2.656888 | 0.398089 | H | -1.090141 | 4.144049 | -1.089110 |
| C | -1.088531 | 2.026527 | 1.671656 | H | -2.402114 | 3.067298 | -0.582707 |
| C | -1.525787 | 3.620192 | -0.229961 | H | -1.855991 | 4.375878 | 0.492522 |
| C | 2.945892 | -1.050258 | -0.851491 | H | 2.277226 | -0.384327 | 2.189026 |
| C | -2.595558 | -1.444425 | 1.397229 | H | 2.705061 | -2.121776 | -0.886435 |
| O | -2.526444 | 0.510178 | -1.135544 | H | 2.729808 | -0.623561 | -1.833731 |
| O | -3.304439 | -1.992515 | -0.844350 | H | -3.685995 | -1.157733 | -1.180230 |
| O | 2.576593 | -0.943418 | 1.455079 | H | -3.415819 | -0.724166 | 1.474751 |
| O | 4.320261 | -0.848934 | -0.594790 | H | -2.951529 | -2.412914 | 1.762138 |
| H | -1.377400 | -3.582459 | 0.113437 | H | -1.776007 | -1.101602 | 2.036411 |
| H | -0.898960 | -2.726697 | -1.353988 | H | 4.434926 | -1.079729 | 0.343781 |
| H | 0.141441 | -2.221984 | 1.489908 | - | - | - | - |

**Table S49.** Atomic coordinates (Å) of **4b**-6 obtained at the B3LYP-D3BJ/6-31G(d) level of theory in the gas phase.

| C | 0.420139 | -0.895713 | -0.138926 | H | 0.486129 | -3.032883 | -0.327250 |
| --- | --- | --- | --- | --- | --- | --- | --- |
| C | -0.134106 | -2.270965 | 0.155582 | H | -0.277001 | 1.829775 | -1.721119 |
| C | -1.588974 | -2.476701 | -0.272632 | H | 1.944175 | 2.447935 | -1.514960 |
| C | -2.465693 | -1.271446 | 0.065863 | H | 2.022492 | 2.113131 | 0.190918 |
| C | -1.830344 | -0.041929 | -0.585552 | H | 2.140211 | 0.025370 | -2.034700 |
| C | -0.370343 | 0.163158 | -0.446319 | H | 3.454275 | 0.506833 | -0.959314 |
| C | 0.117418 | 1.576804 | -0.728417 | H | -1.616677 | 2.491015 | 0.137204 |
| C | 1.656174 | 1.709098 | -0.760887 | H | -0.431488 | 4.241570 | -1.181024 |
| C | 2.369720 | 0.388572 | -1.024640 | H | 0.891734 | 4.244514 | -0.000883 |
| C | 1.927922 | -0.687390 | -0.023798 | H | -0.727750 | 4.754109 | 0.488624 |
| C | -0.534377 | 2.600288 | 0.252603 | H | 0.863356 | 2.513964 | 1.945841 |
| C | -0.177040 | 4.040220 | -0.134141 | H | -0.770545 | 2.990928 | 2.379345 |
| C | -0.196193 | 2.326745 | 1.724539 | H | -0.433013 | 1.296464 | 2.013439 |
| C | 2.768608 | -1.960975 | -0.208077 | H | 1.779614 | 0.519246 | 1.539773 |
| C | -2.579657 | -1.028235 | 1.583218 | H | 2.434947 | -2.725716 | 0.506214 |
| O | -2.554255 | 0.746771 | -1.188802 | H | 2.647915 | -2.355084 | -1.221994 |
| O | -3.756112 | -1.465663 | -0.475494 | H | -3.969908 | -0.619083 | -0.916743 |
| O | 2.240797 | -0.309581 | 1.339386 | H | -1.605172 | -0.894826 | 2.063675 |
| O | 4.144878 | -1.683301 | -0.029544 | H | -3.185249 | -0.135611 | 1.769204 |
| H | -2.006169 | -3.376658 | 0.192674 | H | -3.084269 | -1.885648 | 2.039225 |
| H | -1.639523 | -2.624199 | -1.358089 | H | 4.198254 | -1.212078 | 0.819825 |
| H | -0.018329 | -2.448075 | 1.235247 | - | - | - | - |

**Table S50.** Atomic coordinates (Å) of **4b**-7 obtained at the B3LYP-D3BJ/6-31G(d) level of theory in the gas phase.

| C | 0.552594 | -0.753787 | 0.171457 | H | 0.925175 | -2.861581 | 0.369702 |
| --- | --- | --- | --- | --- | --- | --- | --- |
| C | 0.114045 | -2.150376 | 0.546978 | H | -0.466083 | 1.894327 | -1.513118 |
| C | -1.159095 | -2.600140 | -0.170311 | H | 1.832938 | 1.323323 | -1.797782 |
| C | -2.254029 | -1.537551 | -0.081508 | H | 1.879047 | 2.799210 | -0.854423 |
| C | -1.699343 | -0.211642 | -0.614836 | H | 3.435045 | 1.170280 | 0.172846 |
| C | -0.312356 | 0.187677 | -0.283517 | H | 2.094137 | 1.424153 | 1.268984 |
| C | 0.061764 | 1.628646 | -0.590559 | H | 0.353650 | 2.640189 | 1.296630 |
| C | 1.578496 | 1.747417 | -0.817995 | H | -1.730950 | 1.358840 | 1.671849 |
| C | 2.352049 | 1.021178 | 0.284840 | H | -2.561360 | 2.315908 | 0.433330 |
| C | 2.039615 | -0.480558 | 0.308447 | H | -1.980411 | 3.088780 | 1.919416 |
| C | -0.404825 | 2.649352 | 0.501233 | H | 0.484736 | 4.361130 | -0.562357 |
| C | -1.748688 | 2.328142 | 1.165221 | H | -1.245872 | 4.125755 | -0.854587 |
| C | -0.458947 | 4.064367 | -0.092895 | H | -0.687697 | 4.802620 | 0.683993 |
| C | 2.789741 | -1.205761 | -0.845710 | H | 3.417716 | -1.300316 | 1.367182 |
| C | -2.741538 | -1.318394 | 1.364616 | H | 2.438311 | -2.234629 | -0.938650 |
| O | -2.429547 | 0.483733 | -1.317728 | H | 2.624152 | -0.697622 | -1.802375 |
| O | -3.347051 | -1.931974 | -0.888218 | H | -3.636732 | -1.108394 | -1.328932 |
| O | 2.485593 | -1.062787 | 1.531831 | H | -3.180888 | -2.251406 | 1.731049 |
| O | 4.180769 | -1.310388 | -0.538936 | H | -1.936289 | -1.016384 | 2.041801 |
| H | -1.530179 | -3.544080 | 0.244690 | H | -3.514122 | -0.543861 | 1.377715 |
| H | -0.949606 | -2.774056 | -1.232897 | H | 4.589835 | -0.445557 | -0.698238 |
| H | -0.029569 | -2.166722 | 1.635823 | - | - | - | - |

**Table S51.** Atomic coordinates (Å) of **4b**-8 obtained at the B3LYP-D3BJ/6-31G(d) level of theory in the gas phase.

| C | 0.181676 | -1.001601 | 0.246299 | H | -0.453423 | -3.039571 | 0.459675 |
| --- | --- | --- | --- | --- | --- | --- | --- |
| C | -0.838706 | -2.035563 | 0.662336 | H | 0.594105 | 1.612529 | -1.672772 |
| C | -2.210666 | -1.850604 | 0.012301 | H | 2.568487 | 0.424556 | -1.542307 |
| C | -2.659235 | -0.388651 | 0.042795 | H | 2.939149 | 1.724151 | -0.420394 |
| C | -1.576937 | 0.457693 | -0.629587 | H | 3.597772 | -0.517597 | 0.462973 |
| C | -0.153539 | 0.191689 | -0.305980 | H | 2.404512 | 0.254376 | 1.507469 |
| C | 0.816855 | 1.311226 | -0.642269 | H | -0.585651 | 2.728999 | 0.139066 |
| C | 2.288650 | 0.858109 | -0.579928 | H | 0.853102 | 4.718451 | 0.198598 |
| C | 2.553043 | -0.180019 | 0.515027 | H | 0.884980 | 3.972292 | -1.408132 |
| C | 1.633551 | -1.400503 | 0.423773 | H | 2.256602 | 3.771982 | -0.302310 |
| C | 0.495350 | 2.573611 | 0.221228 | H | 1.912006 | 2.378460 | 1.877595 |
| C | 1.162926 | 3.826748 | -0.358236 | H | 0.442590 | 3.268923 | 2.277078 |
| C | 0.829566 | 2.415287 | 1.709197 | H | 0.388876 | 1.505544 | 2.131876 |
| C | 2.022904 | -2.358687 | -0.739521 | H | 2.643999 | -2.364312 | 1.790549 |
| C | -2.858746 | 0.140857 | 1.477051 | H | 3.081850 | -2.619481 | -0.628131 |
| O | -1.919274 | 1.345763 | -1.405840 | H | 1.439018 | -3.283610 | -0.627320 |
| O | -3.865193 | -0.258140 | -0.682265 | H | -3.745271 | 0.550860 | -1.218672 |
| O | 1.706438 | -2.166654 | 1.635750 | H | -3.175800 | 1.187724 | 1.438300 |
| O | 1.892218 | -1.812286 | -2.038103 | H | -3.648981 | -0.440250 | 1.962401 |
| H | -2.963564 | -2.474326 | 0.506918 | H | -1.947451 | 0.076243 | 2.079871 |
| H | -2.175531 | -2.163952 | -1.038735 | H | 0.973497 | -1.510770 | -2.133522 |
| H | -0.918534 | -1.988224 | 1.756681 | - | - | - | - |

**Table S52.** Atomic coordinates (Å) of **4b**-9 obtained at the B3LYP-D3BJ/6-31G(d) level of theory in the gas phase.

| C | 0.546153 | -0.822910 | 0.053355 | H | 0.728505 | -2.945190 | -0.024552 |
| --- | --- | --- | --- | --- | --- | --- | --- |
| C | 0.036502 | -2.205158 | 0.385187 | H | -0.108231 | 1.736195 | -1.785853 |
| C | -1.372807 | -2.504123 | -0.132550 | H | 2.047167 | 2.501427 | -1.398726 |
| C | -2.323972 | -1.323272 | 0.058493 | H | 1.973585 | 2.252090 | 0.326756 |
| C | -1.692756 | -0.109792 | -0.626220 | H | 2.389760 | 0.062456 | -1.749546 |
| C | -0.260075 | 0.175651 | -0.387431 | H | 3.578210 | 0.686851 | -0.586426 |
| C | 0.192663 | 1.585369 | -0.740692 | H | -1.650215 | 2.502329 | -0.148966 |
| C | 1.719053 | 1.790832 | -0.633993 | H | -0.359515 | 4.190354 | -1.455387 |
| C | 2.510398 | 0.493072 | -0.746344 | H | 0.809441 | 4.329103 | -0.129304 |
| C | 2.028147 | -0.533338 | 0.289752 | H | -0.871054 | 4.812738 | 0.121644 |
| C | -0.591752 | 2.656132 | 0.079338 | H | 0.610215 | 2.707670 | 1.919043 |
| C | -0.228843 | 4.075447 | -0.373041 | H | -1.061073 | 3.209859 | 2.129453 |
| C | -0.418923 | 2.500666 | 1.596003 | H | -0.692957 | 1.494677 | 1.933767 |
| C | 2.925582 | -1.779949 | 0.261699 | H | 1.543642 | 0.648955 | 1.785389 |
| C | -2.564336 | -0.990657 | 1.543420 | H | 3.948731 | -1.457827 | 0.500673 |
| O | -2.398804 | 0.599486 | -1.340055 | H | 2.613868 | -2.475628 | 1.042963 |
| O | -3.561101 | -1.609962 | -0.561584 | H | -3.770753 | -0.806531 | -1.078966 |
| O | 2.205551 | -0.042663 | 1.631877 | H | -1.634746 | -0.789739 | 2.085517 |
| O | 2.865673 | -2.492127 | -0.965114 | H | -3.213778 | -0.113277 | 1.626503 |
| H | -1.786505 | -3.391710 | 0.358979 | H | -3.071733 | -1.838043 | 2.014830 |
| H | -1.332293 | -2.722741 | -1.206306 | H | 3.306981 | -1.959536 | -1.643262 |
| H | 0.081979 | -2.320180 | 1.478938 | - | - | - | - |

**Table S53.** Conformational analysis of the B3LYP-D3BJ/6-31G(d) optimized conformers of **4c** in the gas phase (T=298.15 K)

| Conformer | E (Hartree) | C (Hartree) | G (kcal/mol) | ΔG (kcal/mol) | Population |
| --- | --- | --- | --- | --- | --- |
| **4c**-1 | -886.857357 | 0.324798 | -556299.180567 | 0.0 | 48.73% |
| **4c**-2 | -886.857015 | 0.324715 | -556299.018077 | 0.162489 | 37.04% |
| **4c**-3 | -886.855666 | 0.325326 | -556297.788254 | 1.392313 | 4.64% |
| **4c**-4 | -886.854169 | 0.323964 | -556297.703626 | 1.476941 | 4.02% |
| **4c**-5 | -886.854106 | 0.324061 | -556297.603106 | 1.577461 | 3.39% |
| **4c**-6 | -886.853482 | 0.323859 | -556297.338232 | 1.842334 | 2.17% |

*^a^*Electronic energy obtained at M06-2X-D3/6-311+G(2d,p) level of theory; *^b^*Thermal correction to Gibbs free energy obtained at B3LYP-D3BJ/6-31G(d) level of theory; *^c^*Gibbs free energy (E + C); *^d^*The relative Gibbs free energy; *^e^*The Boltzmann distribution of each conformer.

**Table S54.** Atomic coordinates (Å) of **4c**-1 obtained at the B3LYP-D3BJ/6-31G(d) level of theory in the gas phase.

| C | -0.089183 | -0.950988 | -0.010096 | H | 0.244638 | -2.618926 | 1.274300 |
| --- | --- | --- | --- | --- | --- | --- | --- |
| C | 0.798937 | -2.050986 | 0.518701 | H | -0.073955 | 1.838958 | -1.651557 |
| C | 2.117335 | -1.534230 | 1.090081 | H | -2.619479 | 1.253830 | -0.161734 |
| C | 2.784188 | -0.568394 | 0.117649 | H | -2.371604 | 1.642801 | -1.845058 |
| C | 1.812192 | 0.574502 | -0.211823 | H | -3.184027 | -0.706655 | -1.495936 |
| C | 0.359279 | 0.302035 | -0.279067 | H | -1.604289 | -0.668968 | -2.302609 |
| C | -0.523246 | 1.457748 | -0.724109 | H | 0.585873 | 2.951580 | 0.329311 |
| C | -1.980272 | 1.035424 | -1.023085 | H | -0.738717 | 3.113977 | 2.385672 |
| C | -2.130731 | -0.444143 | -1.362506 | H | -0.329452 | 1.419032 | 2.087317 |
| C | -1.537767 | -1.329680 | -0.263840 | H | -1.965611 | 2.017083 | 1.755723 |
| C | -0.463138 | 2.650071 | 0.283476 | H | -0.946812 | 4.127906 | -1.251029 |
| C | -0.899997 | 2.271752 | 1.703693 | H | -2.340723 | 3.641406 | -0.268658 |
| C | -1.264670 | 3.848793 | -0.239311 | H | -1.116827 | 4.719390 | 0.409595 |
| C | -2.365298 | -1.280877 | 1.037676 | H | -1.397788 | -2.782326 | -1.586541 |
| C | 3.191377 | -1.241395 | -1.208935 | H | -1.863434 | -1.905876 | 1.792110 |
| O | 2.296993 | 1.671947 | -0.480786 | H | -2.411711 | -0.261861 | 1.424002 |
| O | 3.932375 | -0.017224 | 0.737512 | H | 4.004622 | 0.882570 | 0.364201 |
| O | -1.633210 | -2.718082 | -0.647637 | H | 2.337297 | -1.657497 | -1.753987 |
| O | -3.691521 | -1.712309 | 0.812403 | H | 3.678976 | -0.502103 | -1.851844 |
| H | 2.798123 | -2.361876 | 1.317339 | H | 3.904410 | -2.044724 | -0.998654 |
| H | 1.935420 | -0.994993 | 2.027671 | H | -3.602277 | -2.559293 | 0.341799 |
| H | 0.986235 | -2.775881 | -0.284387 | - | - | - | - |

**Table S55.** Atomic coordinates (Å) of **4c**-2 obtained at the B3LYP-D3BJ/6-31G(d) level of theory in the gas phase.

| C | -0.079910 | -0.951491 | -0.018531 | H | 0.252989 | -2.641279 | 1.237112 |
| --- | --- | --- | --- | --- | --- | --- | --- |
| C | 0.806388 | -2.059703 | 0.491050 | H | -0.036049 | 1.859965 | -1.624084 |
| C | 2.127352 | -1.556759 | 1.068342 | H | -2.631332 | 1.270527 | -0.240412 |
| C | 2.789398 | -0.566705 | 0.117305 | H | -2.312959 | 1.641204 | -1.914455 |
| C | 1.815318 | 0.583574 | -0.176535 | H | -3.139905 | -0.707762 | -1.580426 |
| C | 0.364388 | 0.307007 | -0.264662 | H | -1.536730 | -0.688415 | -2.322995 |
| C | -0.513145 | 1.463958 | -0.717199 | H | 0.560993 | 2.937310 | 0.397485 |
| C | -1.957647 | 1.038585 | -1.072343 | H | -0.826143 | 3.081363 | 2.405140 |
| C | -2.090965 | -0.443719 | -1.409702 | H | -0.446301 | 1.381358 | 2.093083 |
| C | -1.522165 | -1.333789 | -0.295041 | H | -2.055346 | 2.024931 | 1.713389 |
| C | -0.487193 | 2.643234 | 0.306220 | H | -0.895275 | 4.145332 | -1.225387 |
| C | -0.982381 | 2.253888 | 1.704049 | H | -2.334157 | 3.649003 | -0.316434 |
| C | -1.260126 | 3.853011 | -0.233620 | H | -1.140249 | 4.713856 | 0.434013 |
| C | -2.350744 | -1.249770 | 1.000631 | H | -2.451703 | -2.988017 | -0.583954 |
| C | 3.185314 | -1.206546 | -1.228819 | H | -1.843753 | -1.828776 | 1.784026 |
| O | 2.297472 | 1.691912 | -0.404018 | H | -2.447051 | -0.214109 | 1.342412 |
| O | 3.941741 | -0.031112 | 0.744013 | H | 4.005916 | 0.880206 | 0.397943 |
| O | -1.530498 | -2.700275 | -0.714528 | H | 3.899156 | -2.015325 | -1.043354 |
| O | -3.627097 | -1.818262 | 0.695245 | H | 2.326499 | -1.608352 | -1.776352 |
| H | 2.809865 | -2.389669 | 1.270218 | H | 3.669698 | -0.452204 | -1.856622 |
| H | 1.952498 | -1.040930 | 2.020682 | H | -4.076000 | -2.030059 | 1.526264 |
| H | 0.979089 | -2.766900 | -0.329856 | - | - | - | - |

**Table S56.** Atomic coordinates (Å) of **4c**-3 obtained at the B3LYP-D3BJ/6-31G(d) level of theory in the gas phase.

| C | -0.071498 | -0.920405 | -0.119554 | H | 0.393682 | -2.526243 | 1.166661 |
| --- | --- | --- | --- | --- | --- | --- | --- |
| C | 0.917299 | -1.928425 | 0.414071 | H | -0.366933 | 1.956695 | -1.586494 |
| C | 2.171626 | -1.290112 | 1.006739 | H | -2.821935 | 0.957457 | -0.158189 |
| C | 2.732907 | -0.224403 | 0.074476 | H | -2.634883 | 1.523858 | -1.800119 |
| C | 1.642944 | 0.822049 | -0.198536 | H | -3.117139 | -0.931862 | -1.733528 |
| C | 0.232488 | 0.390988 | -0.304826 | H | -1.526628 | -0.616680 | -2.435005 |
| C | -0.771585 | 1.466142 | -0.690466 | H | 0.151903 | 3.002302 | 0.473163 |
| C | -2.166329 | 0.898819 | -1.033669 | H | -2.277285 | 1.675683 | 1.800794 |
| C | -2.115225 | -0.550053 | -1.508249 | H | -1.198125 | 2.868669 | 2.519494 |
| C | -1.457183 | -1.452937 | -0.462234 | H | -0.583686 | 1.259273 | 2.116600 |
| C | -0.854780 | 2.585348 | 0.395268 | H | -1.509324 | 4.093956 | -1.043379 |
| C | -1.250106 | 2.060919 | 1.781082 | H | -2.835261 | 3.390348 | -0.098975 |
| C | -1.789313 | 3.715990 | -0.052791 | H | -1.739448 | 4.554512 | 0.650955 |
| C | -2.352685 | -1.614121 | 0.789566 | H | -0.881829 | -2.781882 | -1.800377 |
| C | 3.196827 | -0.797613 | -1.279949 | H | -2.339101 | -0.702967 | 1.389331 |
| O | 2.002307 | 1.981415 | -0.397699 | H | -3.383353 | -1.787922 | 0.439636 |
| O | 3.823050 | 0.417845 | 0.710945 | H | 3.783699 | 1.340660 | 0.392459 |
| O | -1.371449 | -2.800646 | -0.963485 | H | 2.387068 | -1.286476 | -1.832215 |
| O | -1.918689 | -2.664369 | 1.630455 | H | 3.592618 | 0.013418 | -1.899147 |
| H | 2.936781 | -2.047298 | 1.210352 | H | 3.997170 | -1.523534 | -1.105565 |
| H | 1.930664 | -0.807755 | 1.961850 | H | -1.818412 | -3.435882 | 1.046945 |
| H | 1.185691 | -2.635415 | -0.382535 | - | - | - | - |

**Table S57.** Atomic coordinates (Å) of **4c**-4 obtained at the B3LYP-D3BJ/6-31G(d) level of theory in the gas phase.

| C | 0.215733 | -0.827107 | -0.023388 | H | -0.183264 | -2.514484 | -1.261909 |
| --- | --- | --- | --- | --- | --- | --- | --- |
| C | -0.694775 | -1.945351 | -0.481031 | H | 0.041720 | 2.261332 | 1.249499 |
| C | -2.059332 | -1.461836 | -0.968933 | H | 2.474651 | 1.810067 | 1.812222 |
| C | -2.680908 | -0.477144 | 0.013225 | H | 1.375468 | 0.575993 | 2.400710 |
| C | -1.707212 | 0.686908 | 0.226862 | H | 3.030737 | 0.426906 | -0.174472 |
| C | -0.250837 | 0.421400 | 0.241638 | H | 3.372209 | -0.478759 | 1.289659 |
| C | 0.643461 | 1.579134 | 0.638888 | H | 1.758848 | 1.735579 | -1.211019 |
| C | 1.795855 | 1.017364 | 1.485826 | H | 2.270726 | 4.194845 | -1.040882 |
| C | 2.560735 | -0.042178 | 0.698296 | H | 2.898730 | 3.306563 | 0.348901 |
| C | 1.676477 | -1.210070 | 0.223771 | H | 1.409349 | 4.254301 | 0.503975 |
| C | 1.130347 | 2.396217 | -0.597923 | H | -0.588106 | 2.024769 | -1.905027 |
| C | 1.979461 | 3.600413 | -0.167898 | H | -0.728907 | 3.492180 | -0.934710 |
| C | -0.024974 | 2.866574 | -1.491413 | H | 0.366700 | 3.449359 | -2.332924 |
| C | 2.375566 | -1.864065 | -0.990902 | H | 1.215710 | -1.938997 | 2.001729 |
| C | -2.974264 | -1.108504 | 1.389233 | H | 2.195788 | -1.259644 | -1.885476 |
| O | -2.176894 | 1.799327 | 0.454056 | H | 3.457849 | -1.853450 | -0.785535 |
| O | -3.882317 | 0.031162 | -0.538232 | H | -3.928715 | 0.953470 | -0.219863 |
| O | 1.678040 | -2.267878 | 1.214937 | H | -3.429843 | -0.357088 | 2.041568 |
| O | 1.937653 | -3.177251 | -1.268387 | H | -3.682125 | -1.933208 | 1.260743 |
| H | -2.737082 | -2.308289 | -1.124748 | H | -2.072136 | -1.488365 | 1.880616 |
| H | -1.953940 | -0.950515 | -1.933718 | H | 1.885847 | -3.617878 | -0.402228 |
| H | -0.814066 | -2.658116 | 0.346402 | - | - | - | - |

**Table S58.** Atomic coordinates (Å) of **4c**-5 obtained at the B3LYP-D3BJ/6-31G(d) level of theory in the gas phase.

| C | 0.085358 | -0.896280 | -0.005237 | H | -0.301433 | -2.657143 | -1.169140 |
| --- | --- | --- | --- | --- | --- | --- | --- |
| C | -0.817834 | -2.030370 | -0.435719 | H | -0.116020 | 2.180510 | 1.274964 |
| C | -2.156640 | -1.552536 | -0.998244 | H | 2.191655 | 1.608581 | 2.118191 |
| C | -2.809225 | -0.528190 | -0.074850 | H | 0.997901 | 0.380944 | 2.508299 |
| C | -1.834555 | 0.632372 | 0.155211 | H | 3.064766 | 0.310765 | 0.247056 |
| C | -0.386668 | 0.349725 | 0.248490 | H | 3.042758 | -0.729697 | 1.639061 |
| C | 0.511530 | 1.473651 | 0.720527 | H | 1.803604 | 1.581407 | -1.012804 |
| C | 1.541968 | 0.847219 | 1.675806 | H | 1.426352 | 4.105132 | 0.685239 |
| C | 2.389430 | -0.192554 | 0.944071 | H | 2.427508 | 4.002924 | -0.770886 |
| C | 1.558654 | -1.249446 | 0.164051 | H | 2.867191 | 3.073007 | 0.664432 |
| C | 1.153759 | 2.268301 | -0.453597 | H | -0.462299 | 1.982478 | -1.901591 |
| C | 2.021008 | 3.422687 | 0.064909 | H | -0.618134 | 3.449427 | -0.933157 |
| C | 0.101844 | 2.796967 | -1.436854 | H | 0.585898 | 3.367625 | -2.237471 |
| C | 2.225163 | -1.518343 | -1.202341 | H | 1.441445 | -2.407565 | 1.756937 |
| C | -3.173637 | -1.115784 | 1.303972 | H | 1.646812 | -2.271823 | -1.755342 |
| O | -2.299660 | 1.758253 | 0.313551 | H | 2.241055 | -0.597578 | -1.791677 |
| O | -3.977057 | -0.026493 | -0.698179 | H | -4.022717 | 0.908687 | -0.419103 |
| O | 1.645140 | -2.540309 | 0.817906 | H | -2.299559 | -1.485829 | 1.850353 |
| O | 3.563970 | -1.934261 | -1.029227 | H | -3.653864 | -0.341388 | 1.910007 |
| H | -2.837826 | -2.396637 | -1.152181 | H | -3.881762 | -1.938893 | 1.166690 |
| H | -2.005112 | -1.075123 | -1.973981 | H | 3.520913 | -2.660266 | -0.383219 |
| H | -0.979219 | -2.691589 | 0.425740 | - | - | - | - |

**Table S59.** Atomic coordinates (Å) of **4c**-6 obtained at the B3LYP-D3BJ/6-31G(d) level of theory in the gas phase.

| C | 0.067092 | -0.836213 | -0.003902 | H | -0.545758 | -2.503656 | -1.194642 |
| --- | --- | --- | --- | --- | --- | --- | --- |
| C | -0.980944 | -1.833552 | -0.449744 | H | 0.258723 | 2.136017 | 1.422932 |
| C | -2.259887 | -1.190824 | -0.983536 | H | 2.772535 | 1.594527 | 1.516669 |
| C | -2.757792 | -0.088390 | -0.056811 | H | 1.670476 | 0.512072 | 2.339644 |
| C | -1.634934 | 0.936393 | 0.120578 | H | 2.912859 | 0.030684 | -0.413973 |
| C | -0.233995 | 0.467102 | 0.238753 | H | 3.343605 | -0.805531 | 1.071154 |
| C | 0.766885 | 1.521998 | 0.669211 | H | 0.093238 | 2.916174 | -0.775737 |
| C | 1.977218 | 0.862947 | 1.343161 | H | 1.639026 | 2.630619 | -2.579712 |
| C | 2.516302 | -0.319213 | 0.543153 | H | 1.195861 | 1.000194 | -2.058904 |
| C | 1.462444 | -1.399016 | 0.270192 | H | 2.769411 | 1.691555 | -1.609689 |
| C | 1.074906 | 2.525704 | -0.489391 | H | 1.446577 | 4.175570 | 0.895427 |
| C | 1.706450 | 1.918608 | -1.749342 | H | 2.926375 | 3.418612 | 0.275404 |
| C | 1.903715 | 3.714784 | 0.012000 | H | 1.979229 | 4.484002 | -0.764906 |
| C | 2.011002 | -2.312513 | -0.850217 | H | 0.935070 | -1.800172 | 2.131356 |
| C | -3.151160 | -0.613261 | 1.339294 | H | 1.868121 | -1.826950 | -1.821060 |
| O | -1.941541 | 2.122132 | 0.224931 | H | 3.094341 | -2.418727 | -0.683033 |
| O | -3.872255 | 0.551036 | -0.649979 | H | -3.772969 | 1.493127 | -0.410447 |
| O | 1.348450 | -2.293305 | 1.405791 | H | -3.969002 | -1.332562 | 1.231583 |
| O | 1.393525 | -3.580789 | -0.910751 | H | -2.318695 | -1.098628 | 1.859726 |
| H | -3.044047 | -1.944038 | -1.118086 | H | -3.502757 | 0.221127 | 1.954151 |
| H | -2.072202 | -0.741597 | -1.966569 | H | 1.299690 | -3.863236 | 0.015821 |
| H | -1.208967 | -2.489106 | 0.402543 | - | - | - | - |

**Table S60.** Conformational analysis of the B3LYP-D3BJ/6-31G(d) optimized conformers of **4d** in the gas phase (T=298.15 K)

| Conformer | E (Hartree) | C (Hartree) | G (kcal/mol) | ΔG (kcal/mol) | Population |
| --- | --- | --- | --- | --- | --- |
| **4d**-1 | -886.85604 | 0.324616 | -556298.46854 | 0.0 | 34.17% |
| **4d**-2 | -886.854671 | 0.323966 | -556298.017649 | 0.450891 | 15.96% |
| **4d**-3 | -886.855411 | 0.325059 | -556297.795677 | 0.672863 | 10.97% |
| **4d**-4 | -886.855116 | 0.325148 | -556297.554875 | 0.913665 | 7.30% |
| **4d**-5 | -886.855383 | 0.325463 | -556297.524538 | 0.944002 | 6.94% |
| **4d**-6 | -886.855087 | 0.32517 | -556297.522842 | 0.945698 | 6.92% |
| **4d**-7 | -886.855101 | 0.325223 | -556297.498327 | 0.970213 | 6.64% |
| **4d**-8 | -886.854096 | 0.324672 | -556297.213342 | 1.255198 | 4.10% |
| **4d**-9 | -886.853729 | 0.324394 | -556297.157857 | 1.310683 | 3.74% |
| **4d**-10 | -886.854093 | 0.324886 | -556297.077429 | 1.391111 | 3.26% |

*^a^*Electronic energy obtained at M06-2X-D3/6-311+G(2d,p) level of theory; *^b^*Thermal correction to Gibbs free energy obtained at B3LYP-D3BJ/6-31G(d) level of theory; *^c^*Gibbs free energy (E + C); *^d^*The relative Gibbs free energy; *^e^*The Boltzmann distribution of each conformer.

**Table S61.** Atomic coordinates (Å) of **4d**-1 obtained at the B3LYP-D3BJ/6-31G(d) level of theory in the gas phase.

| C | -0.216336 | -0.820430 | 0.287107 | H | 0.071728 | -2.333190 | 1.750266 |
| --- | --- | --- | --- | --- | --- | --- | --- |
| C | 0.586597 | -1.966970 | 0.857282 | H | 0.065819 | 1.819861 | -1.645353 |
| C | 2.035755 | -1.601355 | 1.169974 | H | -2.464038 | 2.113822 | -1.054825 |
| C | 2.676847 | -0.867418 | -0.002957 | H | -1.948808 | 0.709460 | -1.958427 |
| C | 1.836135 | 0.374030 | -0.324999 | H | -2.528465 | 0.778494 | 1.043430 |
| C | 0.361272 | 0.304265 | -0.209288 | H | -3.531844 | 0.022798 | -0.194653 |
| C | -0.399441 | 1.525045 | -0.696580 | H | 0.925734 | 2.824101 | 0.347095 |
| C | -1.875867 | 1.194148 | -0.977080 | H | -0.465966 | 1.640422 | 2.111041 |
| C | -2.501139 | 0.273555 | 0.071885 | H | -1.836967 | 2.677972 | 1.658625 |
| C | -1.723150 | -1.033779 | 0.232486 | H | -0.363834 | 3.395106 | 2.308145 |
| C | -0.160777 | 2.748421 | 0.247871 | H | -1.730992 | 4.080951 | -0.512424 |
| C | -0.743513 | 2.598985 | 1.657790 | H | -0.353194 | 4.914034 | 0.210494 |
| C | -0.640216 | 4.052176 | -0.402642 | H | -0.196637 | 4.185414 | -1.395926 |
| C | -2.058498 | -2.018052 | -0.917113 | H | -2.284605 | -1.096303 | 2.118168 |
| C | 2.762108 | -1.732547 | -1.276527 | H | -1.814027 | -1.564752 | -1.880869 |
| O | 2.421355 | 1.375192 | -0.733201 | H | -1.447910 | -2.924727 | -0.799180 |
| O | 3.976743 | -0.450137 | 0.371560 | H | 4.099995 | 0.406967 | -0.081158 |
| O | -2.194413 | -1.741079 | 1.398965 | H | 3.242496 | -1.154872 | -2.072240 |
| O | -3.434600 | -2.331595 | -0.930390 | H | 3.376441 | -2.614346 | -1.069045 |
| H | 2.620194 | -2.495749 | 1.412235 | H | 1.780090 | -2.058226 | -1.635105 |
| H | 2.078868 | -0.939616 | 2.043738 | H | -3.642519 | -2.579042 | -0.012310 |
| H | 0.557101 | -2.808062 | 0.149553 | - | - | - | - |

**Table S62.** Atomic coordinates (Å) of **4d**-2 obtained at the B3LYP-D3BJ/6-31G(d) level of theory in the gas phase.

| C | -0.273856 | 0.809243 | 0.317950 | H | -1.332670 | 1.576677 | 1.979555 |
| --- | --- | --- | --- | --- | --- | --- | --- |
| C | -1.574117 | 1.073598 | 1.034138 | H | 1.280371 | -1.168704 | -1.748505 |
| C | -2.397210 | -0.186542 | 1.296519 | H | 3.015449 | 0.442074 | 0.121207 |
| C | -2.449545 | -1.058565 | 0.046445 | H | 3.107085 | 0.268249 | -1.612145 |
| C | -1.012627 | -1.423690 | -0.346148 | H | 2.415143 | 2.572452 | -0.930004 |
| C | 0.046702 | -0.399497 | -0.210275 | H | 1.135189 | 1.778048 | -1.868743 |
| C | 1.422961 | -0.781225 | -0.730852 | H | 1.311264 | -2.796045 | -0.015457 |
| C | 2.402278 | 0.409600 | -0.787252 | H | 2.867533 | -0.865455 | 1.796034 |
| C | 1.691243 | 1.749220 | -0.921534 | H | 2.483356 | -2.547551 | 2.136273 |
| C | 0.708035 | 1.972946 | 0.234065 | H | 1.176199 | -1.363102 | 2.025303 |
| C | 2.016120 | -1.968734 | 0.095204 | H | 3.704186 | -3.335945 | 0.018710 |
| C | 2.137782 | -1.661156 | 1.593320 | H | 3.274448 | -2.646816 | -1.554937 |
| C | 3.360459 | -2.425350 | -0.484643 | H | 4.141963 | -1.666869 | -0.358807 |
| C | -0.016482 | 3.319346 | 0.049979 | H | 1.763914 | 1.263476 | 1.727954 |
| C | -3.111278 | -0.345648 | -1.150737 | H | -0.626936 | 3.531360 | 0.928860 |
| O | -0.811338 | -2.531188 | -0.841088 | H | 0.745035 | 4.108477 | -0.019984 |
| O | -3.153922 | -2.250179 | 0.342546 | H | -2.699996 | -2.940219 | -0.179383 |
| O | 1.396141 | 2.128119 | 1.486951 | H | -4.147201 | -0.104289 | -0.892201 |
| O | -0.896825 | 3.328605 | -1.065449 | H | -2.590777 | 0.574399 | -1.435405 |
| H | -3.414252 | 0.069943 | 1.612148 | H | -3.119334 | -1.021298 | -2.011954 |
| H | -1.946871 | -0.778847 | 2.102713 | H | -0.361128 | 3.349992 | -1.872096 |
| H | -2.157193 | 1.795860 | 0.450108 | - | - | - | - |

**Table S63.** Atomic coordinates (Å) of **4d**-3 obtained at the B3LYP-D3BJ/6-31G(d) level of theory in the gas phase.

| C | -0.443371 | -0.740030 | 0.297858 | H | -0.446189 | -2.296573 | 1.740513 |
| --- | --- | --- | --- | --- | --- | --- | --- |
| C | 0.114320 | -2.037394 | 0.836856 | H | 0.452663 | 1.900388 | -1.501563 |
| C | 1.614719 | -1.980422 | 1.116656 | H | -2.046553 | 2.490688 | -1.259520 |
| C | 2.365658 | -1.398007 | -0.076043 | H | -1.699114 | 0.939902 | -2.001269 |
| C | 1.793984 | -0.010650 | -0.393117 | H | -2.400619 | 1.371044 | 0.952143 |
| C | 0.349625 | 0.244653 | -0.197881 | H | -3.513978 | 0.743090 | -0.260705 |
| C | -0.153668 | 1.606864 | -0.637652 | H | -0.833785 | 2.676246 | 1.113057 |
| C | -1.628501 | 1.498834 | -1.059832 | H | -0.863528 | 4.259426 | -0.860033 |
| C | -2.457945 | 0.797327 | 0.017220 | H | 0.907520 | 4.221244 | -0.875850 |
| C | -1.962913 | -0.626539 | 0.290051 | H | 0.045667 | 4.907582 | 0.511106 |
| C | 0.033450 | 2.728482 | 0.439628 | H | 1.292882 | 3.397532 | 2.067953 |
| C | 0.023847 | 4.106562 | -0.236482 | H | 1.303592 | 1.641735 | 1.857276 |
| C | 1.282162 | 2.596192 | 1.320253 | H | 2.196704 | 2.667707 | 0.726364 |
| C | -2.548730 | -1.606434 | -0.759377 | H | -2.415583 | -0.424625 | 2.194965 |
| C | 2.242591 | -2.266737 | -1.343978 | H | -2.257398 | -1.292170 | -1.764855 |
| O | 2.554419 | 0.831663 | -0.864860 | H | -2.141680 | -2.611223 | -0.580811 |
| O | 3.732173 | -1.254516 | 0.268098 | H | 4.025128 | -0.458521 | -0.216390 |
| O | -2.525538 | -1.117806 | 1.525384 | H | 2.816767 | -1.804478 | -2.152793 |
| O | -3.959515 | -1.617309 | -0.709186 | H | 2.662161 | -3.257299 | -1.142123 |
| H | 2.006683 | -2.975949 | 1.351687 | H | 1.207120 | -2.380122 | -1.681752 |
| H | 1.813486 | -1.339747 | 1.984460 | H | -4.175430 | -1.740498 | 0.231805 |
| H | -0.100198 | -2.847849 | 0.125883 | - | - | - | - |

**Table S64.** Atomic coordinates (Å) of **4d**-4 obtained at the B3LYP-D3BJ/6-31G(d) level of theory in the gas phase.

| C | -0.150690 | -0.857720 | 0.009331 | H | 0.293147 | -2.394720 | 1.408546 |
| --- | --- | --- | --- | --- | --- | --- | --- |
| C | 0.799409 | -1.878848 | 0.588944 | H | -0.278286 | 1.988365 | -1.595572 |
| C | 2.124771 | -1.284604 | 1.061317 | H | -2.795054 | 1.231578 | -0.144784 |
| C | 2.706114 | -0.337452 | 0.018893 | H | -2.556870 | 1.650056 | -1.822691 |
| C | 1.674122 | 0.758582 | -0.269053 | H | -3.256901 | -0.730727 | -1.482345 |
| C | 0.233855 | 0.410586 | -0.291236 | H | -1.677086 | -0.644328 | -2.274408 |
| C | -0.705738 | 1.535277 | -0.692147 | H | 0.333022 | 3.013378 | 0.459049 |
| C | -2.139735 | 1.055072 | -1.004623 | H | -1.034658 | 2.987817 | 2.510585 |
| C | -2.213718 | -0.428544 | -1.340577 | H | -0.493039 | 1.352607 | 2.124580 |
| C | -1.601176 | -1.278737 | -0.221473 | H | -2.173420 | 1.852521 | 1.800416 |
| C | -0.700985 | 2.667457 | 0.386485 | H | -2.621320 | 3.599927 | -0.114789 |
| C | -1.124469 | 2.178860 | 1.777658 | H | -1.454272 | 4.691639 | 0.639050 |
| C | -1.557005 | 3.857175 | -0.063817 | H | -1.247991 | 4.213525 | -1.053402 |
| C | -1.775269 | -2.772400 | -0.572874 | H | -2.064584 | -0.322172 | 1.446746 |
| C | 3.046433 | -1.042478 | -1.309412 | H | -2.786677 | -2.894132 | -0.991352 |
| O | 2.087346 | 1.883259 | -0.543788 | H | -1.058611 | -3.065037 | -1.346444 |
| O | 3.873003 | 0.267717 | 0.545835 | H | 3.874391 | 1.169619 | 0.170624 |
| O | -2.354922 | -1.135171 | 1.005688 | H | 2.172487 | -1.505605 | -1.779139 |
| O | -1.588457 | -3.629492 | 0.533616 | H | 3.465495 | -0.312326 | -2.008821 |
| H | 2.847174 | -2.076314 | 1.287368 | H | 3.799413 | -1.813968 | -1.120622 |
| H | 1.973818 | -0.711925 | 1.985044 | H | -2.068260 | -3.195633 | 1.261437 |
| H | 0.976803 | -2.668002 | -0.154378 | - | - | - | - |

**Table S65.** Atomic coordinates (Å) of **4d**-5 obtained at the B3LYP-D3BJ/6-31G(d) level of theory in the gas phase.

| C | -0.529792 | -0.684170 | 0.203363 | H | -0.727624 | -2.270922 | 1.604164 |
| --- | --- | --- | --- | --- | --- | --- | --- |
| C | -0.107857 | -2.035408 | 0.733995 | H | 0.603135 | 1.813639 | -1.639686 |
| C | 1.378013 | -2.113258 | 1.081852 | H | -1.798037 | 2.648542 | -1.431093 |
| C | 2.237889 | -1.561267 | -0.053285 | H | -1.626300 | 1.030197 | -2.096759 |
| C | 1.795640 | -0.123409 | -0.340119 | H | -2.205740 | 1.656814 | 0.845055 |
| C | 0.359517 | 0.217641 | -0.283627 | H | -3.431884 | 1.125199 | -0.307747 |
| C | -0.016928 | 1.607114 | -0.761236 | H | -0.652389 | 3.311057 | 0.405122 |
| C | -1.491612 | 1.625536 | -1.184730 | H | 1.504766 | 4.550851 | 0.434503 |
| C | -2.367379 | 1.063851 | -0.066478 | H | 1.018916 | 4.142378 | -1.220281 |
| C | -2.028632 | -0.401358 | 0.229756 | H | 2.285819 | 3.197518 | -0.417323 |
| C | 0.284287 | 2.747772 | 0.287375 | H | 1.647759 | 1.746050 | 1.672197 |
| C | 1.337266 | 3.719151 | -0.259936 | H | 0.771215 | 3.114990 | 2.367251 |
| C | 0.681635 | 2.260429 | 1.687010 | H | -0.057349 | 1.571299 | 2.110947 |
| C | -2.761658 | -1.338329 | -0.761570 | H | -2.375055 | -0.094155 | 2.144013 |
| C | 2.105538 | -2.375781 | -1.355963 | H | -2.472739 | -1.096283 | -1.787707 |
| O | 2.655771 | 0.702327 | -0.640745 | H | -2.466594 | -2.376923 | -0.557063 |
| O | 3.591238 | -1.548916 | 0.359876 | H | 3.958522 | -0.727598 | -0.021818 |
| O | -2.592018 | -0.788807 | 1.502505 | H | 2.429465 | -3.404430 | -1.168950 |
| O | -4.161761 | -1.182718 | -0.666348 | H | 1.082471 | -2.389268 | -1.746034 |
| H | 1.674896 | -3.144847 | 1.300458 | H | 2.757977 | -1.941639 | -2.119879 |
| H | 1.586372 | -1.517937 | 1.979153 | H | -4.356820 | -1.247347 | 0.284983 |
| H | -0.352187 | -2.803364 | -0.014259 | - | - | - | - |

**Table S66.** Atomic coordinates (Å) of **4d**-6 obtained at the B3LYP-D3BJ/6-31G(d) level of theory in the gas phase.

| C | -0.297901 | -0.823882 | 0.132640 | H | -0.208454 | -2.277159 | 1.670103 |
| --- | --- | --- | --- | --- | --- | --- | --- |
| C | 0.371768 | -2.012821 | 0.777572 | H | 0.197041 | 1.841177 | -1.684651 |
| C | 1.831032 | -1.763657 | 1.155810 | H | -2.282184 | 1.889182 | 0.013173 |
| C | 2.582792 | -1.094613 | 0.010651 | H | -2.101987 | 2.145745 | -1.698831 |
| C | 1.867136 | 0.216040 | -0.342794 | H | -3.364771 | 0.034389 | -1.182104 |
| C | 0.389065 | 0.272381 | -0.280384 | H | -1.878287 | -0.311795 | -2.065543 |
| C | -0.248687 | 1.582418 | -0.715533 | H | 1.247743 | 2.784677 | 0.236302 |
| C | -1.782146 | 1.494284 | -0.879991 | H | 0.088924 | 1.517193 | 2.077095 |
| C | -2.274631 | 0.073178 | -1.116895 | H | -1.361152 | 2.518070 | 1.832918 |
| C | -1.815059 | -0.867334 | 0.003113 | H | 0.139329 | 3.260036 | 2.366414 |
| C | 0.156454 | 2.738124 | 0.257303 | H | 0.007566 | 4.904149 | 0.378709 |
| C | -0.269772 | 2.484897 | 1.709481 | H | -0.058476 | 4.276539 | -1.276017 |
| C | -0.374123 | 4.086811 | -0.243506 | H | -1.468939 | 4.133293 | -0.210836 |
| C | -2.367716 | -2.282663 | -0.246322 | H | -2.131869 | 0.388462 | 1.495196 |
| C | 2.649454 | -1.969077 | -1.257259 | H | -2.021598 | -2.656410 | -1.214991 |
| O | 2.552542 | 1.157573 | -0.736233 | H | -2.011258 | -2.965001 | 0.535999 |
| O | 3.895994 | -0.788386 | 0.442455 | H | 4.116546 | 0.047293 | -0.013002 |
| O | -2.406739 | -0.515572 | 1.276396 | H | 3.206871 | -1.435731 | -2.033533 |
| O | -3.782170 | -2.266562 | -0.285556 | H | 3.181699 | -2.896539 | -1.023908 |
| H | 2.329213 | -2.699440 | 1.431205 | H | 1.659627 | -2.215770 | -1.655377 |
| H | 1.885999 | -1.098747 | 2.026480 | H | -4.052630 | -1.800907 | 0.524580 |
| H | 0.298033 | -2.883663 | 0.110814 | - | - | - | - |

**Table S67.** Atomic coordinates (Å) of **4d**-7 obtained at the B3LYP-D3BJ/6-31G(d) level of theory in the gas phase.

| C | -0.314379 | 0.767652 | 0.307191 | H | -1.540065 | 1.595413 | 1.836218 |
| --- | --- | --- | --- | --- | --- | --- | --- |
| C | -1.670756 | 0.992569 | 0.931310 | H | 1.319998 | -1.302287 | -1.656016 |
| C | -2.424147 | -0.296489 | 1.249195 | H | 3.331237 | 0.290459 | -1.102060 |
| C | -2.380023 | -1.256022 | 0.065322 | H | 1.967548 | 0.896644 | -2.012418 |
| C | -0.912356 | -1.535855 | -0.277918 | H | 2.422487 | 1.299636 | 0.988572 |
| C | 0.074147 | -0.436603 | -0.188710 | H | 2.616171 | 2.526457 | -0.253079 |
| C | 1.462923 | -0.756087 | -0.715691 | H | 1.479330 | -2.597588 | 0.352450 |
| C | 2.263994 | 0.521008 | -1.025280 | H | 2.869400 | -2.061436 | 2.259637 |
| C | 2.050946 | 1.625696 | 0.011860 | H | 1.682744 | -0.761622 | 2.090167 |
| C | 0.575910 | 2.000211 | 0.169703 | H | 3.365925 | -0.515918 | 1.573155 |
| C | 2.190707 | -1.777937 | 0.218923 | H | 3.177440 | -2.791749 | -1.444042 |
| C | 2.543938 | -1.240644 | 1.610544 | H | 4.218556 | -1.622897 | -0.609787 |
| C | 3.429449 | -2.370570 | -0.464204 | H | 3.854576 | -3.174987 | 0.146719 |
| C | 0.106879 | 2.858449 | -1.033725 | H | 0.735725 | 2.456961 | 2.078455 |
| C | -3.069685 | -0.685615 | -1.190366 | H | 0.870139 | 3.636562 | -1.192903 |
| O | -0.619322 | -2.657639 | -0.688631 | H | 0.043482 | 2.243947 | -1.934272 |
| O | -2.998560 | -2.474878 | 0.434844 | H | -2.479940 | -3.162420 | -0.026900 |
| O | 0.417982 | 2.903769 | 1.278229 | H | -2.620523 | 0.252528 | -1.531672 |
| O | -1.168081 | 3.434256 | -0.837837 | H | -3.003577 | -1.416691 | -2.002259 |
| H | -3.465489 | -0.082586 | 1.513791 | H | -4.127013 | -0.511910 | -0.966832 |
| H | -1.968791 | -0.803639 | 2.109027 | H | -1.123680 | 3.870811 | 0.029992 |
| H | -2.247114 | 1.633671 | 0.252301 | - | - | - | - |

**Table S68.** Atomic coordinates (Å) of **4d**-8 obtained at the B3LYP-D3BJ/6-31G(d) level of theory in the gas phase.

| C | 0.851473 | 0.086115 | 0.311741 | H | 1.914026 | 1.153074 | 1.810104 |
| --- | --- | --- | --- | --- | --- | --- | --- |
| C | 1.363910 | 1.382921 | 0.890863 | H | -1.648475 | -1.113663 | -1.514403 |
| C | 0.264918 | 2.405445 | 1.167097 | H | -0.331838 | -3.326952 | -1.253400 |
| C | -0.663442 | 2.540622 | -0.034487 | H | 0.523176 | -2.002759 | -2.023821 |
| C | -1.247033 | 1.161043 | -0.360417 | H | 0.731692 | -2.742156 | 0.947239 |
| C | -0.407857 | -0.043756 | -0.182917 | H | 1.937591 | -3.155758 | -0.258881 |
| C | -1.021660 | -1.352176 | -0.648423 | H | -1.325591 | -2.640367 | 1.060666 |
| C | 0.085572 | -2.330825 | -1.072961 | H | -3.552101 | -3.515390 | 0.434787 |
| C | 1.175087 | -2.412638 | -0.001341 | H | -2.461960 | -3.674441 | -0.948969 |
| C | 1.856465 | -1.061928 | 0.236089 | H | -3.653764 | -2.364536 | -0.908353 |
| C | -1.962785 | -2.021786 | 0.412131 | H | -3.408480 | -0.425160 | 0.767598 |
| C | -2.959686 | -2.950856 | -0.294201 | H | -3.297437 | -1.626063 | 2.069396 |
| C | -2.718770 | -1.056677 | 1.332834 | H | -2.035528 | -0.400679 | 1.882616 |
| C | 2.893687 | -0.792221 | -0.886715 | H | 2.105918 | -1.301229 | 2.174861 |
| C | 0.058871 | 3.068359 | -1.290049 | H | 3.478478 | -1.716908 | -1.012315 |
| O | -2.382033 | 1.110212 | -0.830642 | H | 2.383467 | -0.574968 | -1.827843 |
| O | -1.728672 | 3.412192 | 0.301432 | H | -2.497643 | 3.057601 | -0.185534 |
| O | 2.681841 | -1.126810 | 1.414006 | H | 0.880985 | 2.420747 | -1.611895 |
| O | 3.740920 | 0.303587 | -0.612677 | H | -0.659477 | 3.147499 | -2.112024 |
| H | 0.695876 | 3.381956 | 1.413683 | H | 0.455857 | 4.066294 | -1.079048 |
| H | -0.342232 | 2.092891 | 2.026010 | H | 4.075510 | 0.152313 | 0.287847 |
| H | 2.123005 | 1.785359 | 0.209255 | - | - | - | - |

**Table S69.** Atomic coordinates (Å) of **4d**-9 obtained at the B3LYP-D3BJ/6-31G(d) level of theory in the gas phase.

| C | -0.415053 | -0.753347 | 0.160833 | H | -0.477392 | -2.187400 | 1.711992 |
| --- | --- | --- | --- | --- | --- | --- | --- |
| C | 0.124137 | -2.003783 | 0.813188 | H | 0.233923 | 1.793407 | -1.730907 |
| C | 1.604928 | -1.910292 | 1.173884 | H | -1.963725 | 2.030606 | 0.349541 |
| C | 2.407621 | -1.372458 | -0.004805 | H | -1.969298 | 2.522895 | -1.339983 |
| C | 1.841677 | -0.000639 | -0.399698 | H | -3.407013 | 0.548509 | -0.979774 |
| C | 0.386261 | 0.247969 | -0.283303 | H | -2.026859 | 0.072737 | -1.985505 |
| C | -0.103930 | 1.625559 | -0.698268 | H | 1.636711 | 2.706996 | -0.068608 |
| C | -1.637860 | 1.738103 | -0.653354 | H | 0.885760 | 1.577329 | 1.988206 |
| C | -2.317127 | 0.418508 | -0.983183 | H | -0.638775 | 2.465273 | 1.977988 |
| C | -1.930467 | -0.649859 | 0.052014 | H | 0.884060 | 3.329645 | 2.230039 |
| C | 0.570180 | 2.743375 | 0.163916 | H | -0.988506 | 4.284435 | 0.045549 |
| C | 0.412645 | 2.512637 | 1.672208 | H | 0.649324 | 4.911646 | 0.252509 |
| C | 0.058249 | 4.131794 | -0.240718 | H | 0.138438 | 4.286912 | -1.323648 |
| C | -2.569195 | -2.004150 | -0.332768 | H | -3.354191 | -0.595806 | 1.348170 |
| C | 2.352260 | -2.292299 | -1.240696 | H | -2.264914 | -2.310309 | -1.343240 |
| O | 2.623254 | 0.823555 | -0.869192 | H | -2.270624 | -2.780842 | 0.371605 |
| O | 3.755320 | -1.203300 | 0.396079 | H | 4.064447 | -0.424164 | -0.105829 |
| O | -2.423283 | -0.302407 | 1.351550 | H | 1.334886 | -2.428007 | -1.622428 |
| O | -3.989630 | -1.926820 | -0.209952 | H | 2.961267 | -1.857286 | -2.039108 |
| H | 1.996751 | -2.887037 | 1.477837 | H | 2.769189 | -3.270117 | -0.979543 |
| H | 1.744411 | -1.225172 | 2.018641 | H | -4.327034 | -1.407496 | -0.956047 |
| H | -0.042079 | -2.871678 | 0.159173 | - | - | - | - |

**Table S70.** Atomic coordinates (Å) of **4d**-10 obtained at the B3LYP-D3BJ/6-31G(d) level of theory in the gas phase.

| C | -0.789838 | -0.337335 | 0.234523 | H | -1.497344 | -1.693940 | 1.712781 |
| --- | --- | --- | --- | --- | --- | --- | --- |
| C | -0.870683 | -1.729102 | 0.815592 | H | 1.175415 | 1.552309 | -1.639386 |
| C | 0.491277 | -2.340354 | 1.139935 | H | -0.747215 | 3.229650 | -1.423802 |
| C | 1.469820 | -2.155290 | -0.016788 | H | -1.174056 | 1.669302 | -2.112071 |
| C | 1.580417 | -0.657523 | -0.311434 | H | -1.492571 | 2.407330 | 0.847575 |
| C | 0.365683 | 0.180160 | -0.256977 | H | -2.822519 | 2.421989 | -0.302204 |
| C | 0.523926 | 1.602618 | -0.761165 | H | 0.543580 | 3.423678 | 0.404794 |
| C | -0.838236 | 2.162501 | -1.191177 | H | 2.353754 | 3.616926 | -1.276593 |
| C | -1.862611 | 1.944746 | -0.077625 | H | 3.237872 | 2.298441 | -0.486971 |
| C | -2.098328 | 0.453116 | 0.185918 | H | 2.996367 | 3.845323 | 0.359176 |
| C | 1.225233 | 2.571566 | 0.270285 | H | 1.867565 | 2.757870 | 2.330867 |
| C | 2.532551 | 3.117260 | -0.316733 | H | 0.546090 | 1.599816 | 2.117678 |
| C | 1.467740 | 1.983582 | 1.666240 | H | 2.193716 | 1.165421 | 1.635771 |
| C | -3.047871 | -0.129069 | -0.893047 | H | -2.329620 | 0.627809 | 2.134045 |
| C | 1.016240 | -2.874481 | -1.302711 | H | -3.887984 | 0.575399 | -0.997879 |
| O | 2.679312 | -0.202541 | -0.626219 | H | -2.531902 | -0.196614 | -1.853472 |
| O | 2.742198 | -2.638772 | 0.371698 | H | 3.375882 | -2.005888 | -0.019565 |
| O | -2.858062 | 0.282169 | 1.397517 | H | 0.045123 | -2.522947 | -1.665956 |
| O | -3.513573 | -1.425266 | -0.582562 | H | 1.759380 | -2.711631 | -2.089633 |
| H | 0.392843 | -3.406821 | 1.370581 | H | 0.953008 | -3.949041 | -1.104779 |
| H | 0.925529 | -1.856688 | 2.023792 | H | -3.842734 | -1.369573 | 0.330753 |
| H | -1.431102 | -2.357202 | 0.112017 | - | - | - | - |

**References**

[1] JIA Y Z, YANG Y P, CHENG S W. Phytochemistry. 184, 112678 (2021).

[2] SHANG S Z, HAN Y S, SHI Y M. Nat Prod Bioprospect. 3, 56−60 (2013).

[3] XIAO W L, LI R T, HUANG S X. Nat Prod Rep. 25, 871−891 (2008).

[4] SHI Y M, XIAO W L, PU J X. Nat Prod Rep, 32, 367−410 (2015).

[5] XU H C, HU K, SUN H D. Nat Prod Bioprospect. 9, 165−173 (2019).

[6] WANG B, HU K, LI X N. Bioorg Chem. 110, 104785 (2021).

[7] CHEN Y N, ZHOU H Y, LIU X H. Biochem. Syst. Ecol. 63, 17−19 (2015).

[8] LIU M, HU Z X, LUO Y Q. Nat Prod Bioprospect. 7, 257−262 (2017).

[9] ZHANG X J, YANG G Y, WANG R R. Chem. Biodiversity. 7, 2692−2701 (2010).

[10] LIU J, PANDEY P, WANG X. J Nat Prod. 82, 2842−2851 (2019).

[11] KUO Y H, LI S Y, HUANG R L. J. Nat. Prod. 64, 1608−1608 (2001).

[12] LIU Y, YU H Y, WANG Y M. J Nat Prod. 80, 1117−1124 (2017).

[13] YANG Y P, JIAN Y Q, LIU Y B. Front Chem. 9, 808870 (2021).

[14] MA W H, HE J C, LI L. Helv. Chim. Acta. 92, 2086−2091 (2009).

[15] HUANG R M, HUANG H J, ZHANG N L. J Asian Nat Prod Res. 14, 1116−1121 (2012).

[16] CHEN Y N, LI N, ZHU Y H. Nat. Prod. Commun. 8, 1121−1122 (2013).

[17] PU J X, GAO X M, LEI C. Nat. Prod. Commun. 56, 1143−1146 (2008).

[18] LI C, LI N, YUE J. Nat Prod Res. 31, 1598−1603 (2017).

[19] WILLOUGHBY P H, JANSMA M J, HOYE T R. Nat. Protoc. 9, 643−660 (2014).

[20] GRIMBLAT N, ZANARDI M M, SAROTTI A M. J Org Chem. 80, 12526−12534 (2015).

[21] KIEM P V, MINH C V, NHIEM N X. Magn. Reson. Chem. 52, 51−56 (2014).

[22] CHYU C F, KE M R, CHANG Y S. Helv. Chim. Acta. 90, 1514−1521 (2007).

[23] SARKANEN K V, WALLIS A F A. J. Heterocycl. Chem. 10, 1025−1027 (1973).

[24] KILIDHAR S B, PARTHASARATHY M R, SHARMA P. Phytochemistry. 21, 796−797 (1982).

[25] TSUKAMOTO H, HISADA S, NISHIBE S. Chem. Pharm. Bull. 32, 4482−4489 (1984).

[26] WEI X-L, CHEN Y, CHEN X-Y. Chem. Nat. Compd. 51, 819−821 (2015).

[27] SHEN Z B, THEANDER O. Phytochemistry. 24, 364−365 (1985).

[28] LEE D Y, SEO K H, JEONG R H. Molecules. 18, 41−49 (2012).

[29] JIANG B P, LIU Y M, LE L. Cell Physiol Biochem. 34, 1015−1026 (2014).
